# Grambank reveals the importance of genealogical constraints on linguistic diversity and highlights the impact of language loss

**DOI:** 10.1126/sciadv.adg6175

**Published:** 2023-04-19

**Authors:** Hedvig Skirgård, Hannah J. Haynie, Damián E. Blasi, Harald Hammarström, Jeremy Collins, Jay J. Latarche, Jakob Lesage, Tobias Weber, Alena Witzlack-Makarevich, Sam Passmore, Angela Chira, Luke Maurits, Russell Dinnage, Michael Dunn, Ger Reesink, Ruth Singer, Claire Bowern, Patience Epps, Jane Hill, Outi Vesakoski, Martine Robbeets, Noor Karolin Abbas, Daniel Auer, Nancy A. Bakker, Giulia Barbos, Robert D. Borges, Swintha Danielsen, Luise Dorenbusch, Ella Dorn, John Elliott, Giada Falcone, Jana Fischer, Yustinus Ghanggo Ate, Hannah Gibson, Hans-Philipp Göbel, Jemima A. Goodall, Victoria Gruner, Andrew Harvey, Rebekah Hayes, Leonard Heer, Roberto E. Herrera Miranda, Nataliia Hübler, Biu Huntington-Rainey, Jessica K. Ivani, Marilen Johns, Erika Just, Eri Kashima, Carolina Kipf, Janina V. Klingenberg, Nikita König, Aikaterina Koti, Richard G. A. Kowalik, Olga Krasnoukhova, Nora L. M. Lindvall, Mandy Lorenzen, Hannah Lutzenberger, Tânia R. A. Martins, Celia Mata German, Suzanne van der Meer, Jaime Montoya Samamé, Michael Müller, Saliha Muradoglu, Kelsey Neely, Johanna Nickel, Miina Norvik, Cheryl Akinyi Oluoch, Jesse Peacock, India O. C. Pearey, Naomi Peck, Stephanie Petit, Sören Pieper, Mariana Poblete, Daniel Prestipino, Linda Raabe, Amna Raja, Janis Reimringer, Sydney C. Rey, Julia Rizaew, Eloisa Ruppert, Kim K. Salmon, Jill Sammet, Rhiannon Schembri, Lars Schlabbach, Frederick W. P. Schmidt, Amalia Skilton, Wikaliler Daniel Smith, Hilário de Sousa, Kristin Sverredal, Daniel Valle, Javier Vera, Judith Voß, Tim Witte, Henry Wu, Stephanie Yam, Jingting Ye, Maisie Yong, Tessa Yuditha, Roberto Zariquiey, Robert Forkel, Nicholas Evans, Stephen C. Levinson, Martin Haspelmath, Simon J. Greenhill, Quentin D. Atkinson, Russell D. Gray

**Affiliations:** ^1^ Department of Linguistic and Cultural Evolution, Max Planck Institute for Evolutionary Anthropology, Leipzig, Germany.; ^2^ ARC Centre of Excellence for the Dynamics of Language, College of Asia and the Pacific, Australian National University, Canberra, Australia.; ^3^ Department of Linguistics, School of Culture, History and Language, College of Asia and the Pacific, Australian National University, Canberra, Australia.; ^4^ Department of Language and Cognition, Max Planck Institute for Psycholinguistics, Nijmegen, Netherlands.; ^5^ Department of Linguistics, University of Colorado Boulder, Boulder, CO, USA.; ^6^ Department of Human Evolutionary Biology, Harvard University, Cambridge, MA, USA.; ^7^ Human Relation Area Files, Yale University, New Haven, CT, USA.; ^8^ Department of Linguistics and Philology, Uppsala University, Uppsala, Sweden.; ^9^ Department of Linguistics, Faculty of Arts, Radboud University, Nijmegen, Netherlands.; ^10^ Department of Linguistics, School of Languages, Cultures and Linguistics, School of Oriental and African Studies (SOAS), University of London, London, UK.; ^11^ Langage, Langues et Cultures d'Afrique (LLACAN), Centre National de la Recherche Scientifique (CNRS), Villejuif, France.; ^12^ Institut National des Langues et Civilisations Orientales (INALCO), Paris, France.; ^13^ Department of Asian and African Studies, Humboldt-Universität zu Berlin, Berlin, Germany.; ^14^ Frisian and General Linguistics, Department of General Linguistics, Institute for Scandinavian Studies, Christian-Albrechts-Universität zu Kiel, Kiel, Germany.; ^15^ Department of Linguistics, Faculty of Humanities, The Hebrew University of Jerusalem, Jerusalem, Israel.; ^16^ Evolution of Cultural Diversity Initiative, School of Culture, History and Language, College of Asia and the Pacific, The Australian National University, Canberra, ACT, Australia.; ^17^ Faculty of Environment and Information Studies, Keio University SFC (Shonan Fujisawa Campus), Tokyo, Japan.; ^18^ Department of Anthropology and Archaeology, Faculty of Arts, University of Bristol, Bristol, UK.; ^19^ Department of Comparative Cultural Psychology, Max Planck Institute for Evolutionary Anthropology, Leipzig, Germany.; ^20^ Department of Biological Sciences, Institute of Environment, Florida International University, Miami, FL, USA.; ^21^ Research Unit for Indigenous Language, School of Languages and Linguistics, University of Melbourne, Melbourne, Australia.; ^22^ Department of Linguistics, Yale University, New Haven, CT, USA.; ^23^ Department of Linguistics, University of Texas at Austin, Austin, TX, USA.; ^24^ School of Anthropology, University of Arizona, Tucson, AZ, USA.; ^25^ Department of Biology, Turku University, Turku, Finland.; ^26^ Department of Finnish and Finno-Ugric languages, University of Turku, Turku, Finland.; ^27^ Department of Archaeology, Max Planck Institute for the Science of Human History, Jena, Germany.; ^28^ Institute of Slavic Studies, Polish Academy of Sciences, Warsaw, Poland.; ^29^ Zentrum für Kleine und Regionale Sprachen, Friesisches Seminar, Europa-Universität Flensburg, Flensburg, Germany.; ^30^ Centro de Investigaciones Históricas y Antropológicas (CIHA), Santa Cruz de la Sierra, Bolivia.; ^31^ Europa-Universität Flensburg (EUF), Flensburg, Germany.; ^32^ Institute of Linguistics, Leipzig University, Leipzig, Germany.; ^33^ Department of Linguistics, University of Hawaiʻi at Mānoa, Honolulu, HI, USA.; ^34^ Universitas Katolik Weetebula, Sumba Island, Indonesia.; ^35^ Department of Languages and Linguistics, University of Essex, Essex, UK.; ^36^ Department of Linguistics, University of Cologne, Cologne, Germany.; ^37^ Faculty of Languages and Literatures, University of Bayreuth, Bayreuth, Germany.; ^38^ Structure et Dynamique des Langues (SeDyl), Centre National de la Recherche Scientifique (CNRS), Villejuif, France.; ^39^ Sprachwissenschaftliches Seminar, Georg-August-Universität Göttingen, Göttingen, Germany.; ^40^ Division of Psychology and Language Sciences, Faculty of Brain Sciences, University College London (UCL), University of London, London, UK.; ^41^ Institutt for Filosofi, ide- og Kunsthistorie og Klassiske Språk (IFIKK), Det Humanistisk Fakultet, Universitet i Oslo, Oslo, Norway.; ^42^ Department of Comparative Linguistics, University of Zürich, Zürich, Switzerland.; ^43^ Department of Linguistics, European University Viadrina, Frankfur an der Oder, Germany.; ^44^ Department of Linguistics, Stockholm University, Stockholm, Sweden.; ^45^ Centre for Linguistics, Leiden University, Leiden, Netherlands.; ^46^ Department of Linguistics, University of Antwerpen, Antwerpen, Belgium.; ^47^ Department of English Language and Linguistics, University of Birmingham, Birmingham, UK.; ^48^ Facultad de Letras y Ciencias Humanas, Pontificia Universidad Católica del Perú, Lima, Perú.; ^49^ Institute of Estonian and General Linguistics, University of Tartu, Tartu, Estonia.; ^50^ Department of Modern Languages, Uppsala University, Uppsala, Sweden.; ^51^ University of Freiburg, Freiburg, Germany.; ^52^ Universidad de Chile, Santiago, Chile.; ^53^ The Language Conservancy, Bloomington, IN, USA.; ^54^ Department of Linguistics, Quantitative Lexicology and Variational Linguistics (QLVL), KU Leuven, Leuven, Belgium.; ^55^ Division of Ecology and Evolution, Research School of Biology, Australian National University, Canberra, ACT, Australia.; ^56^ Department of Social Anthropology, University of Cambridge, Cambridge, UK.; ^57^ Department of Linguistics, Cornell University, Ithaca, NY, USA.; ^58^ Centre de Recherches Linguistiques sur l'Asie Orientale (CRLAO), École des Hautes Études en Sciences Sociales (EHESS), Aubervilliers, France.; ^59^ Department of Modern Languages, University of Mississippi, Oxford, MS, USA.; ^60^ International College for Postgraduate Buddhist Studies, Tokyo, Japan.; ^61^ Institute for General Linguistics, Westfälische Wilhelms-Universität Münster, Münster, Germany.; ^62^ Department of Chinese Language and Literature, Fudan University, Shanghai, China.; ^63^ Department of Spanish, Linguistics, and Theory of Literature (Linguistics), Faculty of Philology, University of Seville, Seville, Spain.; ^64^ School of Psychology, University of Auckland, Auckland, New Zealand.

## Abstract

While global patterns of human genetic diversity are increasingly well characterized, the diversity of human languages remains less systematically described. Here, we outline the Grambank database. With over 400,000 data points and 2400 languages, Grambank is the largest comparative grammatical database available. The comprehensiveness of Grambank allows us to quantify the relative effects of genealogical inheritance and geographic proximity on the structural diversity of the world’s languages, evaluate constraints on linguistic diversity, and identify the world’s most unusual languages. An analysis of the consequences of language loss reveals that the reduction in diversity will be strikingly uneven across the major linguistic regions of the world. Without sustained efforts to document and revitalize endangered languages, our linguistic window into human history, cognition, and culture will be seriously fragmented.

## Teaser

Grambank reveals global patterns in linguistic diversity and highlights the impact of language loss.

## INTRODUCTION

There are approximately 7000 spoken languages in the world (*1*). These languages vary widely in their structural properties. They vary by the order in which they arrange words and the constructions they use to combine segments in higher-order units. They can also differ markedly in how information is grammatically expressed. Some languages always mark categories such as gender, number, case, and tense, while some never or only optionally mark these categories. Furthermore, sentences that consist of many words in some languages can be translated by a single word in other languages, while the preferred word order varies widely. This linguistic diversity is not randomly distributed. We expect it to be shaped by human cognition (*2*, *3*), geographical proximity (*4*, *5*), and genealogical descent (*6*, *7*). However, an accurate understanding of the actual structural diversity of languages, the factors that shape that variation, and what is at stake when the world loses languages has been hampered by the lack of accessible, systematically sampled, global data. For example, the World Atlas of Language Structures (WALS) (*8*) has incomplete genealogical coverage (*9*) and 84% missing data (see fig. S1).

Here, we introduce Grambank—a systematic sample of the structural diversity of the world’s languages. Grambank is designed to be used to investigate the global distribution of features, language universals, functional dependencies, language prehistory, and interactions between language, cognition, culture, and environment. The Grambank database currently covers 2467 language varieties, capturing a wide range of grammatical phenomena in 195 features, from word order to verbal tense, nominal plurals, and many other well-studied comparative linguistic variables. The dataset includes both varieties classified as “languages” and “dialects” [70 dialects representing 46 languages, resulting in a total of 2430 unique languages; (*1*)]. The coverage spans 215 different language families and 101 isolates from all inhabited continents and geographic regions (see figs. S2 and S3).

Languages are important to cultural identity, health, the preservation of traditional knowledge and institutions, and as a unique window into human history, culture, and cognition (*10*–*12*). However, languages are vanishing at a rate that rivals our biodiversity crisis (*13*, *14*). It is estimated that without intervention approximately one language will be lost every month in the next 40 years (*15*). This tragic situation and its detrimental consequences have prompted the United Nations (UN) to recently announce the UN Decade of Indigenous Languages (*16*). The Grambank dataset is uniquely positioned to showcase the diversity of the world’s languages and the knowledge that we are currently in danger of losing.

Here, we use the Grambank data to answer four long-standing questions about global linguistic diversity that have previously been difficult to answer in a rigorous quantitative manner. What are the relative roles of genealogical inheritance and geographical diffusion in shaping grammatical diversity? How constrained is grammatical evolution? What are the world’s most unusual languages, and what will the consequences of language loss be on our understanding of linguistic diversity?

## RESULTS

### Genealogy versus geography

One of the oldest debates in the field of linguistics concerns the relative roles of genealogical inheritance and geographical diffusion in shaping patterns of linguistic diversity. Proponents of the tree model of linguistic relationships dating back to at least Schleicher in the 1800s have claimed that nested patterns of inherited linguistic features show that genealogy trumps geographic diffusion (*17*). In contrast, defenders of the “wave model” developed by Schmidt (*18*) have argued that cross-cutting patterns of features reflect waves of linguistic diffusion. Considerable dispute still exists today about the relative importance of genealogy versus geography for explaining variation in the grammatical features of the world’s languages (*19*). Nichols (*20*) has claimed that while features such as a distinction between inclusive and exclusive pronouns are genealogically stable, others such as word order are consistent with primarily geographic influences. Campbell (*21*) has questioned whether genealogical signals can be reliably identified in the structural characteristics of languages, given the potential influences of geographic diffusion, homoplasy, and cognitive constraints on these features. Another dimension of this debate focuses on the temporal depth of genealogical and geographic signals in grammar. Dunn *et al.* (*22*) proposed that structural features of language may bear the signals of deep genealogical relationships in Island Melanesia. Matsumae *et al.* (*23*) found an association between the variation in grammatical structures and genetic variation in Northeast Asia that further supports the idea that structural features reflect deep relationships between populations. Ultimately, the dynamics of grammatical feature evolution may be complex, with a small set of features showing stability on language phylogenies and a large number evolving rapidly and showing bursts of contact-related change (*24*).

To go beyond qualitative impressions and a priori commitments to either genealogical inheritance or geographic diffusion as the primary factor shaping grammatical diversity, we estimated the magnitude of spatiophylogenetic effects jointly using approximate Bayesian inference for latent Gaussian models (*25*). We used a maximum clade credibility tree from a recent Bayesian phylogenetic analysis of all extant languages (*26*) to represent language history. Spatial relations were derived from the language locations documented in Glottolog (*1*). While the effect of phylogeny varies markedly between Grambank features, ranging from very strong (0.98) to almost nonexistent (<0.01), overall it is consistently greater than that of space (mean phylogeny = 0.72, standard deviation = 0.26 versus mean space = 0.03, standard deviation = 0.06; see table S1). Figures S4 to S6 illustrate the features with the strongest phylogenetic signal plotted on the global phylogeny with ancestral state reconstruction and figs. S7 to S9 are maps showing the features with the strongest spatial signal. The feature with the strongest phylogenetic signal (0.98) was GB133: “Is a pragmatically unmarked constituent order verb-final for transitive clauses?” The feature with the lowest phylogenetic signal (<0.01) was GB129: “Is there a notably small number, i.e. about 100 or less, of verb roots in the language?” We note that the strong phylogenetic effects should be interpreted with the caveat that it can be difficult to estimate the independent effects of space and phylogeny because language diversification is itself a spatial process [and the global phylogeny (*26*) was informed by language location]. However, only the global phylogeny captures information on established ancestral relationships between languages. The fact that the phylogeny so consistently and decisively outperforms space as a predictor suggests that the modern patterns of linguistic diversity are shaped by genealogical inheritance more than geographical diffusion.

**Fig. 1.**
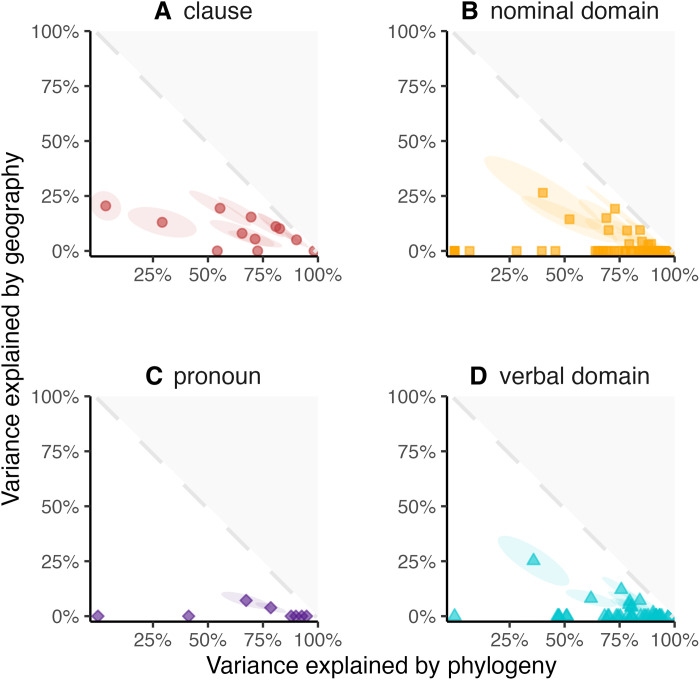
Variance explained by phylogeny and geography. Each point is a Grambank feature. The panels represent different domains of grammar that the features are associated with: (**A**) clausal, (**B**) nominal domain, (**C**) pronominal domain, and (**D**) verbal domain. A high value indicates that a large part of the variance is explained by either space (*y* axis) or phylogeny (*x* axis). The ellipses represent the standard deviation of the joint posterior, tilted for the covariance.

The relative influences of genealogy and geography may not be uniform across different elements of grammar. Linguists (*27*, *28*) have suggested that language contact may have different outcomes for the verbal, pronominal, and nominal domains of grammar in contact languages. Grambank features cover many different domains of grammar (e.g., clausal, nominal, pronominal, and verbal) and thus enable us to test the generality of this claim. We do not find statistical differences across domains in terms of spatial or phylogenetic effects (see Fig. 1 and table S2). Nichols (*20*) makes more specific claims about the areal diffusibility versus phylogenetic inheritance of specific grammatical features in language change in noncontact languages. We matched her predictions with features in Grambank and their respective spatial and phylogenetic effects. We do find support for several features she predicted to show strong phylogenetic effects; however, the same is not true for those predicted to be areal (see fig. S10).

### Constraints on grammar

The Grambank dataset focuses on 195 core grammatical features (see table S4). Even this basic set of features represents an astronomical number (>10^34^) of possible grammars—the possible “design space” (sensu Dennett) (*29*). How constrained is the distribution of the world’s actual realized grammars within this total design space and what are the most important axes of variation? Some have claimed that languages are tightly constrained systems—“un système où tout se tient” [a system where everything fits together; (*30*)]. Many generative linguists assert that human cognition imposes strong constraints on grammatical variation such that only a small number of underlying factors are required to explain the observed diversity (*31*–*33*). In contrast, others have argued that distinct components of language can vary individually, “All parts of a language appear in principle to be independently mobile” (*34*). Grambank’s broad suite of logically independent traits (see the Supplementary Materials, SM1:1), systematically coded across a global sample of languages (see Materials and Methods), makes it an ideal resource for exploring these claims.

We use principal components analysis (PCA) to reduce the dimensions of the Grambank data to a set of orthogonal variables representing the underlying patterns of variation among the grammatical features we consider (see Materials and Methods). A nongraphical Cattel’s Scree test (*35*) showed that the optimal number of components is 19, which explain 49% of the variation among grammars. The first three components returned by the PCA capture only 21% of the variation (9, 7, and 5%, respectively). These results can be compared to similar studies of musical and genetic variation. A recent analysis of cross-cultural musical behavior found that only three components optimally described the variation (*36*). In contrast, an analysis of human genetic variation across Europe in the form of single-nucleotide polymorphisms found that the first and second principal components explained under 1% of the variation (0.3 and 0.15%, respectively) (*37*). This indicates that language structures have greater combinatorial flexibility than musical behavior, but far less than genetic evolution. Grammatical systems are thus neither tightly constrained nor entirely free to vary.

Having eliminated nearly all strict logical dependencies from our dataset (see Supplementary Materials, SM1:1), the sizable fraction of grammatical variation that is explained by a limited set of dimensions could reflect functional or historical constraints on grammar. However, even the broader set of 19 principal components still leaves more than half of the variation unexplained, suggesting that there is a high degree of flexibility in grammatical structures, rather than tight constraints determined by a small number of underlying factors.

It is possible that the principal components we infer are simply clusters of traits that are associated because of shared phylogenetic history, rather than functional constraints on these linguistic systems. To establish whether they correspond to meaningful aspects of design space, we compared these data-driven dimensions to metrics we developed to capture factors linguists have commonly used to describe grammatical variation. The metrics were word order (*38*, *39*), locus of marking [the degree to which a language mainly features head or dependent marking, as described by (*40*)], morphological typology [expression by phonologically fused versus freestanding morphemes, which we call “fusion” (*41*)—not to be confused with Sapir’s notion of “fusional” languages (*42*)], and flexivity [degree of allomorphic variation, as described by (*41*)]. In addition, we calculated an index for use of noun class/gender to further probe this important component of the flexivity score (Materials and Methods and table S6). We found that PC1 correlated most strongly with features capturing fusion, while PC2 correlated most strongly with noun class/gender features (see fig. S15). PC3 did not show a clear association with any of the metrics (see table S3). Hence, while much of the variation in our data falls outside of these metrics, our analysis indicates that at least the first two dimensions of variation in the world’s grammars do have a clear linguistic interpretation, corresponding to the extent to which languages combine elements through “fusion” and use noun class/gender.

**Fig. 2.**
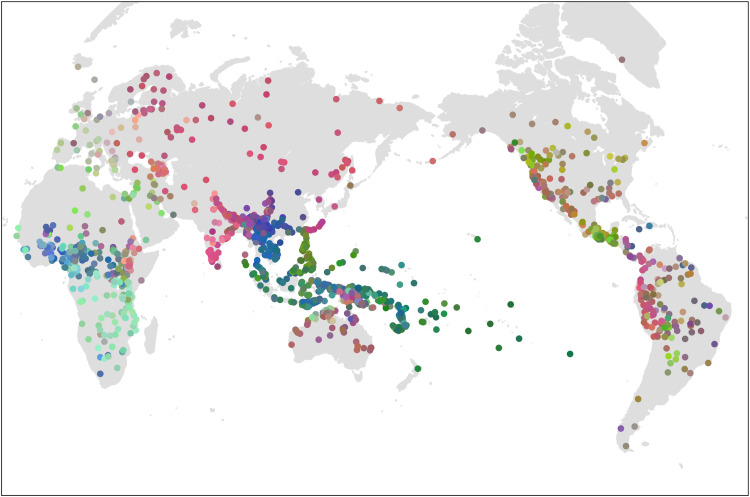
Grammatical similarity in the Grambank sample of languages. The color coding represents the distribution of languages according to the first three principal components (PCs) mapped onto RGB color space (PC1, red; PC2, green; PC3, blue). Similarity in color indicates similarity in grammatical structure on the first three dimensions. See fig. S15 for loading of Grambank features on the first two components and fig. S16 for correlation with theoretical metrics.

**Fig. 3.**
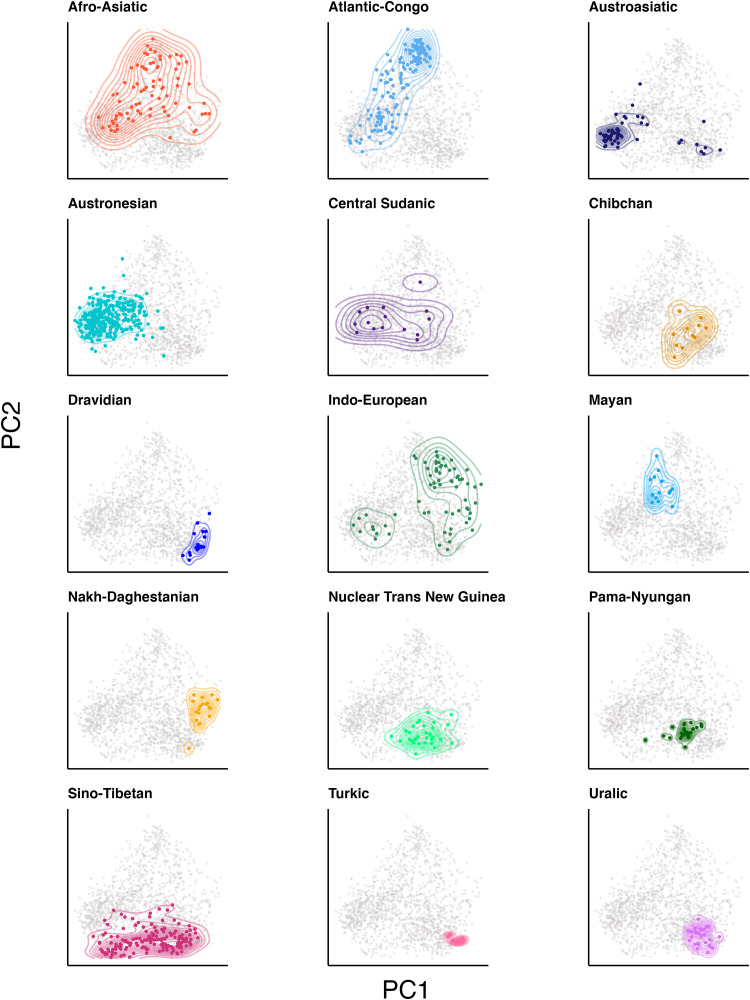
Distribution of the 12 largest families in our dataset in Grambank design space. The *x* axis represents the first principal component (PC1), and the *y* axis represents the second principal component (PC2). All languages are plotted, and for each facet, one family is highlighted in a different color. Austronesian languages, which are known for lacking gender and having little morphology, are found on the far left.

Next, we use these dimensions to examine how history constrains the evolution of languages through this design space. Figure 2 plots the location of the languages in our sample, colored according to the first three principal components. Consistent with our spatiophylogenetic analysis above, this figure reveals macroscale spatial patterns around the globe that appear to mirror the distribution of some major language families. For example, most Austronesian languages in the Pacific are colored dark green, while the Bantu languages in sub-Saharan Africa share a bright turquoise. To examine the connection between history and design space more closely, we map the 15 largest language families in the world onto plots of design space defined by the first two principal components (Fig. 3). Language families such as Austronesian, Nuclear Trans-New Guinea, and Dravidian are tightly packed together, suggesting strong phylogenetic inertia in this part of the design space. However, other families like Afro-Asiatic or Indo-European are more spread out in the Grambank design space, demonstrating high within-family diversity in these dimensions. Within Indo-European, for example, there are two clusters largely corresponding to contact languages and noncontact languages (see fig. S17). The Austroasiatic language family also shows two distinct clusters: languages of the Munda sub-family and the rest of the family (see fig. S18). Language families, then, can be both distinct and diverse samples from the design space.

This mix of both distinctness and diversity within families raises the question: “Is the evolution of the world’s languages through this grammatical design space determined by a set of universal and enduring design constraints, or is the process historically contingent, canalized by culturally evolved, inherently unpredictable and lineage-specific basins of attraction?” For example, we find few languages overall in the upper left corner of Fig. 3, where we would expect (given the loadings on the PCA) languages with little morphology but robust noun class/gender systems.

This question about constraints parallels S. J. Gould’s work exploring the role of historical contingency in biological evolution (*43*). Gould asks, if we were to “replay the tape of life” over and over again, what patterns of current diversity would reliably recur (reflecting universal constraints) versus never evolve again (reflecting historical contingency)? While Gould laments that no such experiment exists in the natural world, the evolution of the world’s languages does contain a natural experiment of this kind. The current linguistic diversity of the Americas has emerged over the past 15 to 30 thousand years, essentially “replaying the tape” of language evolution from a small number of founder lineages.

To answer Gould’s question, we computed pairwise cultural fixation scores (*44*) based on the Grambank data for languages of the world divided into 24 linguistic areas (8 in the Americas and 16 elsewhere) (*45*). Cultural fixation scores are preferable to raw distances because they take into account feature prevalence and inter- and intragroup variation. A low cultural fixation score indicates a close affinity, and a high score indicates greater differentiation. We use a network to visualize these pairwise fixation scores (fig. S20), and use a modularity score to assess the relative independence of network components (see table S7). The low fixation scores between some areas in the Americas reflect shared history, but the negative modularity of the American component of this network (−0.061) indicates that the Americas do not form a separate community cluster from the rest of the world (see fig. S21). These findings suggest that while phylogenetic history clearly matters a lot for explaining global language diversity, there nevertheless appear to be some enduring constraints that shape the cultural evolution of languages over many thousands of years toward predictable regions of grammatical design space.

### Unusual languages

Our understanding of how languages work as systems is strongly informed by the cross-linguistic frequency of grammatical features and their combinations. Prolific language groups (e.g., the Austronesian or Atlantic-Congo families), as well as functional pressures (e.g., the tendency toward harmonic word orders), drive the overall prevalence of certain features and combinations of features. Languages with uncommon features or combinations of features are informative for the study of language because they show the limits of what is possible and can also be rare survivors of deep linguistic lineages.

We investigate unusual combinations of grammatical features by introducing a metric—“unusualness”—that generalizes the notion of cross-linguistic frequency from individual features or combinations of features to entire grammars (see Materials and Methods). According to our metric, a language is more unusual than another if (i) some of its features and/or (ii) some of its combinations of features are more infrequent, comparatively speaking. It should be stressed that this operationalization of unusualness is necessarily restricted to the features present in Grambank; in other words, we make no claims about the unusualness of languages with respect to linguistic features not covered in the database.

The global distribution of unusualness is richly structured (fig. S23). The most unusual languages are most often not members of the largest language families, or if they are, they are found at the geographic periphery of their expansion. In particular, several of the most unusual languages are isolates, i.e. languages with no known connection to any established language family [e.g., Movima (movi1243), Kuot (kuot1243), Hadza (hadz1240), and Yélî Dnye (yele1255)]. Isolates represent 4% of Grambank’s languages in total, but they make up 19% of the most unusual languages. In addition, the distribution of grammatical unusualness displays areal patterning beyond language families, with cultural and historical regions revealing consistent values of unusualness from low (Southeast Asia), mid (southern Africa), to high (northern Africa and Europe); see fig. S23.

To assess the accuracy of these inferences, we built a model to predict unusualness based on language families and cultural regions (see fig. S24 and table S8). The model performs well (Bayesian *R*^2^ = 0.75, see table S8), which suggests that language families and regions strongly predict a given language’s unusualness. In other words, historical factors that have driven regional patterns of lineage loss, such as the expansion of language families and colonial empires, are likely to have been more important in structuring patterns of unusualness than general constraints on grammar.

The existence of unusual languages should not overshadow the fact that all languages in our sample are typically very different from each other. Very few pairs of languages share the same Grambank description (only five; see Manhattan distances in fig. S26). Given that these descriptions are centered on core grammatical features, this entails that each and every language enshrines a unique and irreplaceable source of linguistic knowledge. Thus, in addition to the social and humanitarian consequences (*10*, *11*), each endangered language poses a threat to the understanding of language generally.

### Language loss

**Fig. 4.**
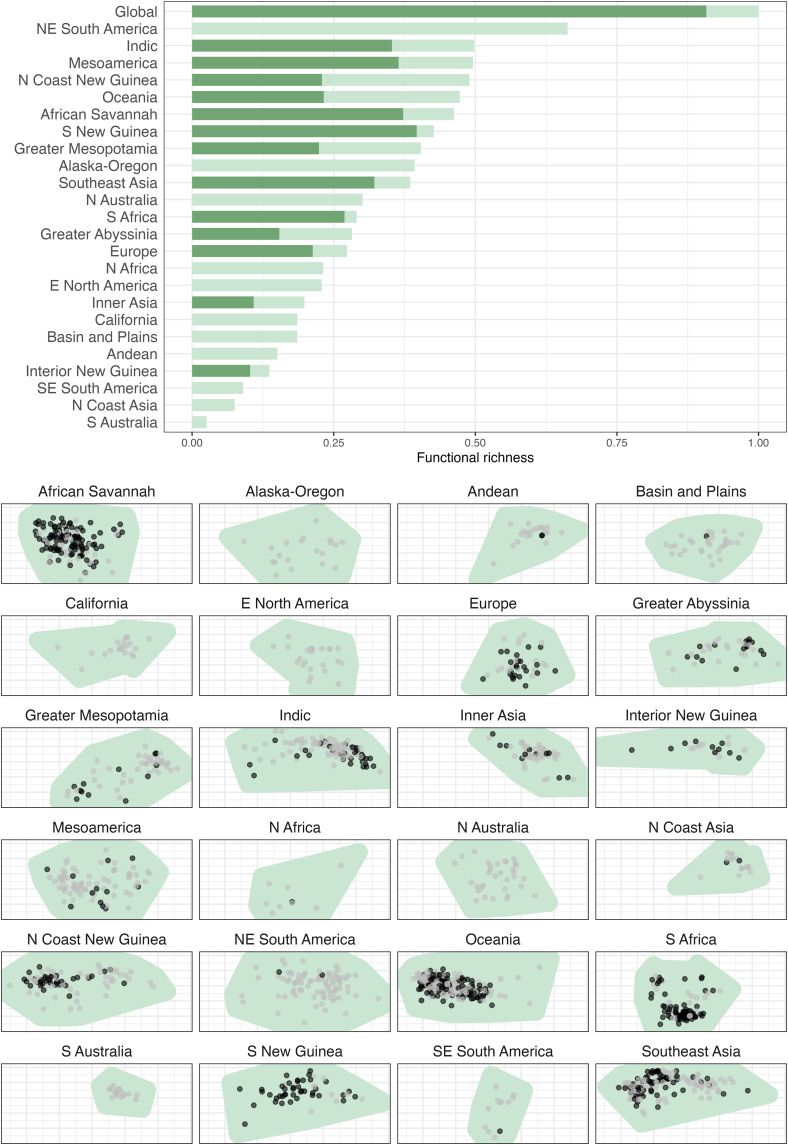
Decline of functional richness associated with language loss. Top: Bars representing functional richness relative to the current diversity of the world’s languages are shown in light green, and functional richness of nonthreatened languages in the same areas are shown in dark green. Functional richness declines in all areas, with some regions showing dramatic decreases. Bottom: Threatened (gray) and nonthreatened (black) languages are plotted over a convex hull (green) that represents the overall area of functional space [*x* and *y*, representing two dimensions of a principal coordinate analysis on the Grambank feature set] occupied by languages of the area.

We investigate the potential loss of linguistic knowledge using contemporary estimates of language endangerment and a new way of quantifying language diversity. Our goal is to provide a bird’s-eye view at both global and regional levels. With this in mind, we applied a metric that is used in ecology termed “functional richness” (*46*, *47*). This metric quantifies the area occupied by a species (languages in our study) in an abstract multidimensional space defined by a set of features and estimates the diversity the data represent. By computing this metric with all languages, and then only with those that are not endangered, we can estimate the potential loss in structural diversity (*48*). We calculated functional richness globally and for each region (see Materials and Methods) (*45*). This allows us to estimate what we will lose collectively if these languages disappear. We found that, although functional richness declines only moderately on a global scale with the loss of languages that are under threat, the consequences of language loss vary markedly across regions (Fig. 4). Regions like Northeast South America, Alaska-Oregon, and northern Australia will be markedly affected because all indigenous languages there are under threat, and so the functional richness that would remain is 0. The pronounced reduction of nearly half the functional space occupied by languages, even in regions with many nonthreatened languages (e.g., Oceania, North Coast New Guinea, Greater Abyssinia, and Greater Mesopotamia), will undermine our ability to investigate the basic structures of language and the diverse expressions used to encode them.

## DISCUSSION

The Grambank data open up the possibility of quantitative cross-linguistic comparison on a scale that was not previously possible. This paper set out to answer four questions about global patterns of grammatical diversity with the Grambank data. What are the relative roles of genealogical inheritance and geographical diffusion in shaping grammatical diversity? How constrained is grammatical evolution? What are the world’s most unusual languages, and what will the consequences of language loss be on our understanding of linguistic diversity? Our analyses revealed that, contrary to widespread claims, genealogy had a dominant role in shaping patterns of grammatical diversity and the distribution of unusual languages. We found that the world’s most unusual languages are often members of small language families or even isolates. Our PCA revealed that grammatical diversity is not captured by a small number of dimensions, as might be expected if there were a tight set of design constraints. Instead, there are substantial degrees of freedom for phylogenetic history to explore different combinations of grammatical features. However, despite the importance of phylogeny for explaining global patterns of grammatical diversity, the fact that the languages of the Americas do not form a separate community cluster from the rest of the world points to the existence of enduring constraints that give rise to the convergent cultural evolution of grammatical features. Last, our analyses show that the impact of language loss will be strikingly uneven across the major linguistic regions of the world, highlighting the need for increased work on language documentation and revitalization in regions such as Northeast South America, Alaska-Oregon, and northern Australia.

We anticipate that future work will use Grambank data to test more nuanced claims about language universals, linguistic areas, and the factors that drive the evolution of linguistic disparity. Grambank will also facilitate the exploration of links between linguistic diversity and a broad array of other cultural and biological traits, ranging from religious beliefs to economic behavior, musical traditions, and genetic lineages. We hope that these links with other facets of human behavior will help make Grambank a key resource not only in linguistics but also in the multidisciplinary endeavor of understanding human diversity.

## MATERIALS AND METHODS

### Data

Grambank is a large-scale database of language structures. The dataset is built on a questionnaire of 195 features. The Grambank dataset version 1.0 contains 2467 language varieties, spanning all continents and major language families. For the analysis in this paper, we chose to remove all but one dialect per language, which leaves us with 2430 languages. The Grambank data gathering procedure progresses per language, i.e., the entire questionnaire is filled in as much as possible for one language at a time based on published grammatical descriptions [typically sources classified as “grammar” or “grammar sketch” in Glottolog; (*1*)]. This procedure leads to high data coverage per language compared to, for example, the WALS (*8*), which is more akin to an anthology of features. Grambank contains 24% missing data, which can be compared to 84% in WALS (fig. S1). The Grambank dataset features great coverage per continent and language family, and fig. S2 shows the Grambank coverage per Glottolog macroarea. For more information and background on the Grambank data project, see the Supplementary Materials, SM1:5. For the analyses in this paper, it was necessary to merge dialects, binarize features with multistate values, crop features and languages with large amounts of missing data, and/or impute the remainder of the missing data.

There are 2467 language varieties in Grambank, including 70 dialects. To maximize the overlap with other data sources used in the analysis [e.g., WALS (*8*), AUTOTYP (*45*), and the global tree (*26*)], we chose to drop all but one dialect per language. If two datasets have entries for two different dialects of the same language, they would not be matched to each other unless they were both reduced to their language level ID. The dialect that was retainedwas the one with the least amount of missing data. The remaining language variety is assigned the glottocode of its parent language variety that is classified as “language” in Glottolog (i.e., not “dialect”). For the comparison of coverage between WALS (*8*) and Grambank (see fig. S1), we also reduce dialects in WALS by keeping the one with the least amount of missing data in the same fashion. This leaves 2430 languages in Grambank and 2435 languages in WALS. There were 35 languoids in WALS that were not mapped to a glottocode and therefore not possible to include in the comparison at all.

There are six features in the Grambank dataset that have multistate values; all others are binary. Multistate features all concern word order: “what is the order of elements X and Y?” with the alternatives “XY,” “YX,” or “both.” They were all split into two features each, of the format “Is the order XY?” and “Is the order YX?” with the “both” values triggering a 1 (yes) for both features. This process gives 201 binary features of the original 195. 

The full dataset contains 24% missing data. To avoid problems of excessive imputation, we first crop the dataset such that we remove features and languages with more than 25% missing data, leaving 1509 languages and 113 binarized features. There remains 4% missing data in the cropped dataset. These missing data are imputed using a random forest trained on the observed values, as implemented in the R package “missForest” v. 1.4 (*49*, *50*). The out-of-bag error rate is estimated at 14%. The random forest technique is entirely naive as to language genealogy or geography; it imputes missing data based on languages with a similar profile regardless of relatedness or spatial distance. For each analysis, we specify if the imputed dataset was used.

We used a Maximum Clade Credibility tree from recent global language phylogeny (*26*), which contains over 6000 languages. As with the Grambank and WALS datasets, we reduced dialects to languages to increase overlap. Tips that are mapped to the same language were reduced to one by choosing one tip in the set at random. We also dropped tips in the global tree that did not correspond to languages in our pruned and imputed dataset. This left 1404 tips in the global tree and languages in the Grambank dataset for the spatiophylogenetic analysis. Information on descriptive status, endangerment status, dialect membership, longitude/latitude, language family, and macroareas were taken from Glottolog (*1*). To group languages into cultural areas for analysis and visualization, we used the classification provided by AUTOTYP (*45*).

All the code and data associated with this paper are published alongside the paper, including data processing, all analysis and scripts generating each plot in this paper. The code and data are found publicly on Zenodo (Grambank v1.0: https://doi.org/10.5281/zenodo.7740140; scripts associated with this paper: https://doi.org/10.5281/zenodo.7740822).

### Analysis: Spatiophylogenetic modeling

The estimation of spatial and phylogenetic effects for each feature of Grambank was calculated using a binomial spatiophylogenetic model following the procedure laid out in (*25*). This model is implemented using Integrated Nested Laplace Approximations (INLAs) of a Bayesian model using the R package INLA v20.03.17 (*51*). We used the binarized, dialect-merged, and cropped version of the Grambank dataset, but not the imputed values.

The model contains two structured random effects: one representing the phylogenetic relationships between languages, and one representing the spatial distances. A key departure from the procedure laid out in prior research (*25*) is that the spatial relationships are represented as spatial coordinates, unlike in the procedural paper where spatial relationships are represented within a spatial mesh. We use coordinates to ensure that spatial and phylogenetic variation is compared on an equal footing, with one phylogenetic taxon and one location per language.

Phylogenetic relationships are drawn from a recently released Bayesian posterior distribution of phylogenetic trees capturing genealogical relationships between the world’s languages (*26*). We use the maximum clade credibility tree derived from this posterior distribution, which incorporates prior information on established genealogical classifications within families (*1*), conservative confidence intervals on the timing of internal diversification and origin of families, a phylogeographic model of language diversification in space, and archaeological and genetic evidence of human expansion around the globe.

Spatial relationships are built from the latitude and longitude of language metadata, collected by Glottolog (*1*). We can only include languages from Grambank that are also represented in the phylogeny. There are 1404 languages that appear in both the dialect-dropped, cropped, and binarized Grambank dataset (see the previous section) and the phylogeny. To maximize overlap, the global tree was also dropped for dialects (dropping all but one tip at random out of sets of tips that are dialects of the same language). The dataset used in this analysis contains 4% missing values, and we did not impute them. We followed the same principles for cropping for missing data as outlined in the previous section, leaving us with 113 features.

The spatiophylogenetic model uses precision matrices to represent the phylogenetic and spatial relationships, which are calculated from covariance matrices. Phylogenetic covariance is estimated through a model of Brownian motion, and spatial covariance is determined through a Matérn covariance function. The phylogenetic covariance matrix is built using the vcv.phylo function from the R package ape v5.4-1 (*52*), and the spatial covariance matrix is built from the varcov.spatial function in the package geoR v1.8-1 (*53*), using the Matérn covariance function with the parameters: sigma = 1.15 and kappa = 2. Covariance matrices are standardized to have a variance of approximately 1 by dividing the matrix by its typical variances, before being inverted to become precision matrices. Penalizing-complexity priors are set for each random effect, which offers a 10% chance of variance being >1, although prior choice has little influence on the results (see below).

#### Spatial parameterization

In addition to the Matérn parameters described above, we test two additional Matérn parameters (kappa and sigma), which iteratively expand the influence of spatial relationships (fig. S11). Increasing the reach of spatial relationships had little influence on our general conclusions (fig. S12).

#### Prior choice

Following earlier research (*25*), priors for both the phylogenetic and spatial effects used the penalized complexity prior (pcprior) distribution with parameters 1 and 0.1, which correspond to an exponential distribution with ~10% of its probability above 1. To test the sensitivity of the results to these priors, we range the probability above 1 to vary from 1 (very strict), 10, 50, and 99% (effectively uniform). The choice of prior had negligible effects on parameter estimates and did not change the mode comparison results (see fig. S13). We used 10% (pcprior = 0.1) for the main analysis.

#### Simulations

To ensure that the spatiophylogenetic model returned statistically valid results, we ran a series of simulations using the phylogenetic and geographic location of the Grambank sample. Simulated binary variables varied across two conditions: the amount of phylogenetic signal (Pagel’s λ of 0.01, 0.3, 0.6, and 0.9) and the proportion at which traits occur (0.1, 0.25, and 0.4)—a total of 12 conditions. Variables were simulated using the geiger v2.0.9 (*54*) function sim.char(). Variables were simulated 15 times per condition. Phylogenetic signal was varied using geiger and the function rescale(), which rescales the phylogeny branch lengths according to the desired parameter. The proportions were gathered by randomly generating the *Q* matrix and repeating the simulation until the desired proportion and signal were retrieved. Figure S14 shows the results of the simulations across three models: spatiophylogenetic (dual process), phylogeny only, and spatial only. In all conditions considered, both the phylogenetic only and dual process models correctly identify the increase of the simulated phylogenetic signal. Our simulation results demonstrate a level of error that is comparable to previous simulation studies (*55*). As the trait distribution approaches parity, there is a decline in the accuracy of the phylogenetic estimate, although estimates still occur in the correct rank order of the phylogenetic signal, and there is no confusion between phylogenetic signal and spatial relationships.

#### Ancestral state reconstruction

To illustrate more clearly the structure of the phylogenetic signal in the three features with the strongest phylogenetic signal, we used the INLA approach to reconstruct ancestral states of protolanguages for each feature, respectively. The analysis is the same as for the main spatiophylogenetic analysis (kappa = 2, sigma = 1.15, dual model with both phylogeny and spatial precision matrices). The key difference lies in the phylogenetic precision matrix, which in this analysis also includes positions for the ancestral language—internal nodes in the tree. These nodes are not associated with feature values; those values are missing. The INLA model estimates predictions for missing values, based on the fitted posterior distribution, thus producing predicted feature values of the ancestral states. Note that these internal node positions are not associated with any spatial information, i.e., we have not inferred any longitude or latitude of proto-languages. Spatial information is, however, included in the overall model as information about the tip values (this means that predictions for internal nodes are made with the spatial field set to zero, that is, with spatial variation estimated from the tips “removed”). See figs. S4 to S6 for tree plots of the result of this analysis. These figures show the three features with the strongest spatial signal out of the whole set and their distribution across the world.

#### Testing the association between domain and spatial and phylogenetic effect

The features of Grambank can be divided into four different domains: clause, verbal, nominal, and pronominal. Fig. 1 shows the mean phylogenetic and spatial effects per feature as grouped by these domains. To test whether domain membership predicts phylogenetic and spatial effects, we used BRMS (Bayesian regression models using “Stan”) models with and without the domains as a predictor and compared their model fit scores. We used a beta distribution since the values are bound between 0 and 1 and compared Watanabe–Akaike Information Criterion (WAIC) scores. The response variable is the mean spatial and phylogenetic effect per feature, respectively, with the default INLA model parameters (kappa = 2, sigma = 1.15, and pc prior = 0.1). Specifically, we ran four BRMS models for the 113 Grambank features: (i) a null model where the intercept predicts the spatial effect for features, (ii) a model where the domain predicts the spatial effect for features, (iii) a null model where the intercept predicts the phylogenetic effect for features, and (iv) a model where the domain predicts the phylogenetic effect for features. The difference in WAIC values between the null and domain models for the effects was smaller than the standard error of this difference, from which we conclude that there is no improvement in predictive accuracy from taking feature domain into account.

### Analysis: PCA

We carried out a traditional nonweighted PCA to derive the dimensions along which data primarily varied. We used the function prcomp in R v4.1.0 (*56*). The data were dialect-merged, binarized, cropped, and imputed for the PCA. It is necessary that the data are binarized because the PCA relies on the mean of each variable, which is not meaningful for multistate features. It is also necessary to remove and/or impute missing data as PCA requires a complete dataset. The variables were scaled to have unit variance and centered. We examined the rotations/loadings of the components for each feature (fig. S15). To evaluate what phenomena most contributed to each component, we also examined the rotated data per language and compared to other aggregate scores capturing known linguistic theoretic concepts.

We compared the rotated data to concepts used in linguistic typology to characterize language variation. For each of these concepts, we created an index that measures, for each language, the occurrence of Grambank feature values that might be expected in a language that perfectly exemplifies the relevant theoretical properties. The concepts we encoded with typological indices are as follows:

1) Word order [the degree to which a language uses structures hypothesized to correlate with verb-object or object-verb word order in (*38*, *39*)].

2) Locus of marking [the degree to which a language mainly features head or dependent marking, as described by (*40*)].

3) Fusion [degree to which a language encodes meanings and functions with bound morphology as opposed to phonologically free-standing markers (*41*)].

4) Flexivity [degree of allomorphic variation (*41*)].

The nature of the questions in the Grambank questionnaire prevents us from exploring other typological concepts like Bickel and Nichols’ “exponence,” which expresses the degree to which individual morphemes encode multiple functions/meanings.

Each of the above metrics was calculated by assigning values (0, 0.5, and 1) to each Grambank feature that expresses information about the phenomenon captured by that metric, according to the extent to which the feature is consistent or inconsistent with the typological phenomenon. For word order, our feature-wise metric values reflect consistency with proposed verb-initial word order patterns. For locus of marking, the feature-wise metric values reflect consistency with proposed head marking patterns. We used these values to calculate per-feature indices of consistency with the metric’s theoretical concept and then expressed a language’s overall score for any metric as the mean of that language’s consistency indices. A value of 0 assigned to a feature indicates that the feature contradicts the pattern or phenomenon measured by a metric. For these features that oppose the patterns captured by the theoretical metrics, we reverse the values of language-specific coding in the consistency index, i.e., 0 becomes 1 and 1 becomes 0. For example, for the word order metric, features related to verb-final orders such as GB022: “Are there prenominal articles?” and GB133: “Is a pragmatically unmarked constituent order verb-final for transitive clauses?” have a “word order point” value of 0. Features associated with verb-initial order such as GB023: “Are there postnominal articles” and GB262: “Is there a clause-initial polar interrogative particle?” are awarded a “word order point” value of 1. If the language value is 1, and the word order point value of that feature is 1, the word order metric consistency index for that feature in the language is 1. Each language will thus be assigned a consistency index of either 0 or 1 for each feature. The assignment of per-feature word order consistency indices based on the interaction of “word order point” feature values and language-specific feature coding and the calculation of mean word order score per language are illustrated in table S9 for four features and three languages.

For our fusion metric, we assigned a value of 0.5 to features that are consistent with the typological pattern of expressing information through phonologically bound morphs but which do not necessarily indicate that grammatical information is expressed by phonologically fused elements. For example, GB075: “Are there postpositions?” encodes whether languages use an element that follows a noun to express adpositional meanings. Both postposition words (which are phonologically independent) and postpositional enclitics (which are phonologically fused) can trigger a 1 value for this feature. Because a 1 value for this feature is not inconsistent with the concept of typological fusion but does not necessarily mean that the language uses phonologically fused enclitics for this function, we assign a value of 0.5 for this feature. This value is multiplied by a language’s feature value to obtain a feature-level index of consistency with fusion (i.e., in languages where this feature is coded 1, the feature index for fusion will be 0.5, while in languages where the feature is coded 0, the feature index for fusion will be 0).

A high score for our word order metric indicates that a language has relatively more order features that have been hypothesized to correlate with verb-initial order than features associated with verb-final order. A high score for the locus of marking metric reflects greater use of head-marking strategies than dependent-marking strategies. A high score for the fusion metric indicates that a language tends to express grammatical meanings through phonologically bound morphemes (e.g., affixes) rather than freestanding words. Last, a high score for the flexivity metric indicates that a language has lexically conditioned allomorphy in multiple grammatical or lexical categories (e.g., noun classes and suppletion in lexical forms).

To test whether the patterns captured by component loadings were best described by these specific typological concepts, rather than broader or more narrowly defined phenomena, we created two additional metrics: (i) noun class/gender and (ii) informativity. The first of these additional metrics encodes noun class/gender (i.e., the degree to which a language categorizes nouns into classes/genders, excluding classifiers). The noun class/gender metric allows us to assess the degree to which any latent pattern we observe is driven by flexivity in general versus the more specific phenomenon of noun class/gender, which makes up a large proportion of the features that contribute to the flexivity score. As expected, we find that flexivity is highly correlated with noun class/gender (*r* = 0.77, *P* < 0.05). We find that noun class/gender is more strongly associated with PC2 (*r* = 0.73, *P* < 0.05) than the more general flexivity metric (*r* = 0.64, *P* < 0.05), suggesting that the pattern captured by that component relates to the more specific concept of noun class/gender. The noun class/gender score was calculated in the same manner as word order, locus of marking, fusion, and flexivity scores.

The second of these measures is informativity, or the degree to which basic grammatical meanings/functions are obligatorily encoded in the grammar (regardless of how, exactly, these meanings are encoded). This captures how much information needs to be specified when making an utterance in a language. For example, does the language have a rule that tense needs to be marked (regardless of how it is marked)? The informativity score allows us to ascertain whether our fusion metric is actually capturing a more general tendency for languages to require more types of information to be obligatorily encoded in grammar. The informativity score was computed in a different way compared to the other metrics. It was calculated by grouping features that pertain to the same grammatical function (reflexive, passive voice, singular number, etc.) and counting that function as present if a language has a positive value for any member of that set. An average was then taken across all available sets for a language, indicating how many of these functions are expressed, by either bound marking or free marking. A language with a low score for this index encodes fewer types of information obligatorily in grammar and may express these meanings optionally or lexically. A language with a high informativity score requires nonoptional expression of many different grammatical functions.

Wordhood (i.e., what constitutes a word) is a concept that is difficult to converge on globally, and there may be biases among grammar writers that create unnecessary connections between grammaticality and phonological fusion of morphemes in some grammars. To evaluate whether our fusion index truly measures the phonological dependence/independence of grammatical material, rather than a more general tendency to express many types of grammatical meanings, we compared our fusion index to the informativity index. The weak correlation between the informativity score and the fusion score (*r* = 0.40, *P* < 0.05) suggests that the fusion index is not merely a measure of informativity but is actually capturing something interesting about the structure of language (i.e., not the bias of authors).

We take all six theoretical scores and compare the score per language to the PCA positions (see fig. S16). PC1 is strongly correlated with the fusion score, PC2 is strongly correlated to noun class/gender, and PC3 is not correlated strongly with any score. To test this more robustly, we also ran an analysis that controls for phylogeny (see table S3). We then ran a phylogenetic generalized least squares analysis (*57*) on each of the first three principal components and each theoretical score. This allows us to assess the correlation of each pairing while controlling for shared ancestry as represented by the global language tree (*26*), which is not the case with the simple Pearson correlation matrix in fig. S16. The principal components and theoretical scores were each divided by their standard deviations to make the coefficients easier to compare. Table S3 shows the results. PC1 correlates most strongly with the fusion score. PC2 correlates most strongly with gender/noun class. PC3 is not strongly correlated with any theoretical score.

### Analysis: Cultural fixation scores

Fixation scores (often abbreviated *F*_ST_) are a way of measuring similarity between groups of data in a dataset. It is commonly used in genetics to study how close different groups of individuals are and how the structure compares to what would have happened if everyone reproduced randomly. The outcome of the analysis is a score for each pairing of groups in your data. A low score indicates that members of those two groups are similar, whereas a high score indicates that they are dissimilar. The value is dependent on both the between-group and within-group variation in the data, as well as the overall frequency of the variable in the entire dataset.

There are several different approaches to fixation scores in the literature. For this study, we used the method proposed by (*58*), which is developed specifically for cultural data. For more on the details of the cultural fixation score and how it differs from other fixation scores, see (*58*).

For the Grambank dataset, we use the groups from the AUTOTYP project (25 cultural areas like “Andean” and “Indic”) and the macroareas from Glottolog 4.0. Each language is associated with one of these regions, and the pairwise cultural fixation scores indicate how likely it is that two areas should be merged or kept separate. This analysis uses the dialect merged, cropped, and binarized dataset (i.e., 1509 languages and 113 features)—but not imputed data. To illustrate the scores, fig. S19 shows a barplot of cultural fixation scores over macroareas and fig. S20 shows the cultural fixation scores over AUTOTYP areas.

To investigate whether the AUTOTYP areas that are found in the Americas do form a distinct cluster, we rendered a network based on the cultural fixation scores and computed the modularity score if we group the nodes into Americas versus not Americas (see fig. S21). We used the function modularity from the R package igraph (*59*), and the score was −0.061. This indicates that a division Americas versus not Americas is not a supported way of dividing up the relationship between languages of AUTOTYP areas given their pairwise cultural fixation scores.

### Analysis: Unusualness

We define unusualness based on the information-theoretic notion of surprisal. According to this measure, a language is considered to be more unusual the rarer its features and/or combinations of features are cross-linguistically. Concretely, we compute the surprisal associated with each language *i*

$$U_{i}=\log(P_{i})$$ (1)

where

$$P_{i}=\mathrm{Pr}(X^{1}=x_{i}^{1},X^{2}=x_{i}^{2},\ldots ,X^{113}=x_{i}^{113})$$ (2)

is the probability of the Grambank description of language *i*. Estimating *P_i_* is complicated by the fact that our sample size is much smaller than the number of possible grammars (i.e., what is referred to as a n<<p scenario in machine learning). We overcome this obstacle by constructing a model-based estimator based on different assumptions about the structure of grammars. We used the dialect-merged, binarized, cropped, and imputed version of the Grambank dataset for the unusualness analysis.

### Probability density estimation

For this analysis, we used the dialect-merged, cropped, imputed, and binarized dataset (see the Supplementary Materials), which contains 1509 languages over 113 features. The possibility space (the number of possible distinct languages in the Grambank description) is 2^113^. However, our goal is to approximate the probability distribution of the Grambank description of the languages that exist today—and not some theoretical distribution of “possible” or “frequent” languages independent from the finite sample we were able to observe. In this regard, our sample is not negligible, specifically when contrasted with the number of languages for which a comprehensive grammar exists (~4000). Nevertheless, a direct estimation of the probability distribution is unfeasible as all Grambank descriptions are unique (and we do not want to assume that all languages not described in Grambank have to be identical to some other Grambank language). To overcome this limitation, we use our understanding of linguistic diversity to develop two estimators for this target probability distribution.

### Bayesian latent class analysis

The first estimation model is based on the idea that some of the strongest regularities in grammar are likely to be confined within bundles of features (e.g., word order of the nominal phrase and locus of marking). The probability of the Grambank description of an unobserved language will thus depend on whether it displays patterns and traits that are regularly found in other languages. Rather than using prebuilt categories for the features, we induce hierarchical clustering. The gap statistic indicates an optimal choice of nine clusters. For each of those clusters, we can then identify a discrete and small number of latent classes that more efficiently capture the variation in the data. We implement this through Bayesian latent class analysis (LCA). For each bundle of features, we find the optimal number of clusters (between 1 and 6) based on the Bayesian Information Criterion (BIC). For all nine bundles, a single cluster turns out to be privileged, which reveals how skewed the representation of different language types is.

### Local kernel density estimation

As an alternative to the method developed above, we implemented a method based on locally smoothing the space of attested grammars. The motivation is that a high density of similar Grambank descriptions points to what is probably a smooth high probability density region—so that Grambank descriptions of unattested languages that are close to many attested ones will get a high probability. We parametrize this approach by constructing an approximation to the probability distribution with an exponential kernel based on Gower’s distance (i.e., the fraction of overall differences between two Grambank descriptions) so that the probability of any specific description is

$$P_{i}^{k}\alpha \sum_{l}\exp(-k\times d_{il})$$ (3)

where the summation is carried over all languages of Grambank (parametrized with *l*), *k* is the kernel parameter, and *d_il_* is Gower’s distance between the target Grambank description *i* and language *l*. It should be noted that we do not calculate the exact probabilities in this case (as this would require estimating this probability on all possible Grambank descriptions), but just a number that is proportional to it—which is sufficient for the purpose of our analyses.

We studied *k* = 1, 5, 10, 15, 20, 25, 30, and 40, covering widely differing scales of locality. To gain an intuition of the effect of this parameter choice, it is instructive to consider how much the presence of a specific Grambank description contributes to the probability distribution near it. To start with, consider that observing one specific Grambank description contributes to its probability a number proportional to exp(0) = 1. We use this contribution as the scale of measurement in these following examples. In the broader case (*k* = 1), observing a Grambank description makes even distant languages substantially more likely: Languages that are 10, 20, and even 50% different get a boost of 0.9, 0.8, and 0.6, respectively. Therefore, even languages that are as similar as they are different from a given language will still receive a large boost from them. On the other hand, the most local case (*k* = 40) contributes to languages that are 10, 20, and 50% different (0.02, 0.0003, and 0.000000002 correspondingly). In this scenario, only very similar languages are taken into account when determining the probability of any Grambank description.

### Comparison between methods

We compare the Bayesian LCA and the kernel approaches (see fig. S22). The Bayesian LCA approach yields almost identical results to those of the least local kernel approaches, suggesting that our derived latent classes are not particularly effective at capturing the complexities of the probability distribution at a small scale. The distributions reflect clearly the scale of smoothing: Models that learn locally (i.e., have large kernel values) result in a heavy concentration around the highest value of the metric such that most languages are unusual. The opposite pattern holds for the LCA and the models with small kernel values: Most languages are concentrated on the lower values of unusualness. Given these findings, for further analyses, we pick the estimator yielding the distribution with the least skewness—in other words, the one that does not concentrate languages in either extreme of the scale (which is kernel 15). Figure S23 shows the distribution of unusualness scores (kernel 15) per language in the world, and fig. S24 shows it as grouped by AUTOTYP areas.

### Unusualness model

We deploy a Bayesian regression model of unusualness. The spatial and phylogenetic effects are both variance covariance (VCV) matrices based on a Brownian motion approach. The spatial data are taken from Glottolog (*1*), and the phylogeny is the global language tree (*26*). This is the same method of generating the VCVs as the INLA modeling, with the same kappa and sigma values (2 and 1.15, respectively) for the spatial VCV. The rest of the analysis is different in that it uses BRMS rather than Bayesian inference for latent Gaussian models (INLA). We use default (uninformative) priors for all coefficients as implemented in the Stan wrapper brms R package (*60*). We ran four independent chains for 6000 iterations, and all parameters of the model showed convergence quickly into the run of each chain. A summary of the model parameters can be found in table S8. The Bayesian *R*^2^ of this model is 0.75 (estimated error = 0.02) (*61*). The posterior predictive distributions of this model (arranged according to cultural areas) can be found in fig. S25.

### Analysis: Manhattan distances

Manhattan distances show the sum total of the number of differences between two records of data, in our case, between pairs of languages. For this metric, we used the binarized version of the dataset, i.e., each language for each feature had a value of 0 or 1 (or missing). If there are 10 binary features, then a Manhattan distance of 4 for a pair of languages would mean that, for four features, they had different values (0 when the other had 1 or vice versa). This measurement is not relative to how many complete pairs of data points there are. If for one feature and one language pair, there is at least one missing data point, that feature is ignored. A Manhattan distance of 0 means for all features the language pair has exactly the same values.

For the calculation in our dataset, we used the dialect-merged and cropped, but not imputed, data. There are 113 features in the dataset that is cropped for missing data, meaning that the maximal possible Manhattan distance between any two languages is 113. The highest value found was 74; the pair consisted of the Sino-Tibetan language Wambule [wamb1257] and the Atlantic-Congo language Bobangi [bang1354]. There were six language pairs with a distance of 0. In each of these cases, the two languages were from the same language family (see table S10). The mean distance was 39. A plot of the distribution is found in fig. S26.

### Analysis: Functional richness

We followed the approach used in ecology where functional richness analyses are commonly based on principal coordinates analysis (also known as classical metric multidimensional scaling), as this maximizes the amount of the total variation in the dataset that can be captured in two dimensions (here, 33%). We calculate this using the R package fundiversity (*62*). The analysis is carried out groupwise within each language area and at a global level. Functional richness is calculated twice, once with all languages in the region and once with endangered languages removed. We use the Agglomerated Endangerment Scale (AES) (*63*) and categorize languages as either nonthreatened or threatened (the latter of which includes all AES categories associated with endangerment or recent dormancy). Of the languages in Grambank, seven languages had no AES value recorded. To avoid overestimating the effects of endangerment, we excluded these languages from the analysis. We used the dialect-merged, binarized, cropped, and imputed dataset for the functional richness analysis.

## Supplementary Materials

### This PDF file includes:

Further Grambank database details Figs. S1 to S26 Tables S1 to S10 References
