# Supplementary material for: Grambank reveals the importance of genealogical constraints on linguistic diversity and highlights the impact of language loss
Source: Sci Adv. 2023 Apr 19;9(16):eadg6175. doi: 10.1126/sciadv.adg6175 (PMC10115409; doi:10.1126/sciadv.adg6175)
Supplement: Supplementary file 1 — Further Grambank database details Figs. S1 to S26 Tables S1 to S10 References [file sciadv.adg6175_sm.pdf]

Science Advances
AAAS

Supplementary Materials for

Grambank reveals the importance of genealogical constraints on linguistic diversity and highlights the impact of language loss

Hedvig Skirgård *et al.*

Corresponding author: Hedvig Skirgård, hedvig_skirgard@eva.mpg.de; Russell D. Gray, russell_gray@eva.mpg.de

Sci. Adv. **9**, eadg6175 (2023)
DOI: 10.1126/sciadv.adg6175

This PDF file includes:

Further Grambank database details
Figs. S1 to S26
Tables S1 to S10
References

SM1 Further Grambank database details

SM1:1 Grambank structure and design

Over 80 contributors have participated in the coding of the Grambank features, and a team of seven feature experts has supported their work. Extensive descriptive and procedural documentation for each feature was used to ensure reliable coding. Formal testing of inter-coder reliability demonstrates a high degree of consistency across coders. Care was taken to remove strict logical dependencies between features to eliminate the problem of non-independent data-points. As is the nature of languages, other kinds of dependencies may remain and are possible to explore with the dataset and to control for given the extensive documentation.

Grambank is available in the Cross-Linguistic Linked Data framework via the Cross-Linguistic Data Format (*64*). The dataset uses Glottolog language codes to identify languages (*1*), ensuring clear identification of languages and compatibility with other linguistic and cultural datasets, such as D-PLACE (*65*).

Institutional history

The Grambank project began as a joint project in 2015 between departments in two Max Planck Institutes (MPI): the Language and Cognition department (L&C) of the MPI of Psycholinguistics in Nijmegen, Netherlands – led by Stephen C. Levinson – and the Department of Linguistic and Cultural Evolution (DLCE) now at the MPI for Evolutionary Anthropology (MPI-EVA) in Leipzig, Germany – led by Russell Gray. This collaboration took place within the larger international research consortium named Glottobank, which also involves the Centre of Excellence for the Dynamics of Language in Canberra, Australia, and the University of Auckland, New Zealand. The Australian National University, University of Kiel, Uppsala University and the School of Oriental and African Studies also take part in the organization of Grambank.

The Grambank database builds on the work by the Nijmegen Typological Survey from the L&C department at MPI-Nijmegen led by Stephen C. Levinson and Harald Hammarström, as well as on the works of the Pioneers of Island Melanesia project and the Sahul survey, led by Ger Reesink and Michael Dunn. Grambank has inherited features (see next section) from these surveys as well as data points. Coders who have contributed to these preceding databases are also attributed as coders in the Grambank dataset. In acknowledgment of the work that went into the Sahul survey design we would like to thank Angela Terrill, Eva Lindström, Gunter Senft, Nicholas Evans, Sjef Barbiers, Mily Crevels, Rob Goedemans, Pieter Muysken, Leon Stassen and Hein van der Voort for their contribution to that questionnaire.

Grambank contains some data points that were originally published elsewhere: Hunter-Gatherer Language Database, SAILS and the aforementioned NTS & Sahul surveys. The database contains imported data points from the typological section of the Hunter-Gatherer database (HG), led by Claire Bowern, Patience Epps and Jane Hill. The HG database does not contain a one-to-one match between its features and features in Grambank. Data-points for import were matched carefully by Harald Hammarström, Thiago Chacon, Hedvig Skirgård, Hannah Haynie, Judith Voss and Jakob Lesage. Grambank also contains imported data-points from the work of Swintha Danielsen on Arawakan languages (*66*). Danielsen's work was based on the Sahul

survey which also serves as the base of Grambank, therefore import was straightforward. Imported datapoints are attributed to the appropriate coders in the Grambank dataset.

Grambank feature selection

The set of features included in Grambank reflect a balance between several design principles and practical pressures. The principles guiding the construction of this database included obtaining maximal coverage of the sorts of typological information contained in source materials that describe the world's languages, constructing a simple data structure with clear and interpretable feature values, and preserving compatibility with legacy data. The Grambank questionnaire was created by a team of linguists in the Glottobank consortium, drawing on experience primarily from the Nijmegen Typological Survey (NTS, *67*), which in turn builds on the Sahul survey. The NTS constituted a core questionnaire upon which Grambank was built, with additional inspiration from the data and experiences of the Pioneers of Island Melanesia project (*22*). The influential typological database WALS (*8*) also inspired features of both the NTS and Grambank. 103 of the 195 features in Grambank are inherited from the questionnaire of the Pioneers of Island Melanesia and 40 from the NTS, making these features well tested and documented.

The questions describe a wide variety of morphosyntactic and lexical features likely to be discussed in a grammatical description, such as word order, the existence of prefixes and suffixes with particular functions, marking of grammatical categories, and agreement rules. Each feature can be coded using grammars and grammar sketches without necessarily requiring the coder to have a comprehensive knowledge of the entire language.

Each feature in the questionnaire is structured in the form of a brief feature name, a feature description, feature ID, and a set of possible feature values. Feature names take the form of a question that typically probes the presence or absence of an individual grammatical element. Feature summaries provide a succinct description of the targeted phenomenon and the criteria that should be used to identify it. A source field is used to cite the resource and page number where the coded information was found. The comments field allows the coder to enter any additional information that may be useful for understanding their response.

Dependencies

Typological surveys that cover a large range of grammatical topics often contain data points that are not logically independent from one another. For example, in a database that has features for the number of case categories and the position of case marking, any language that is coded as having suffixed case marking will also necessarily be coded as having case categories. Such dependencies might complicate the analysis of comparative data. For this reason, the Grambank dataset largely eliminates strict logical dependencies between the features.

It is worth noting that the following Grambank features participate in a near strict logical loop.

- GB020 Are there definite or specific articles?
- GB021: Do indefinite/non-specific nominals commonly have indefinite/non-specific articles?
- GB022: Are there prenominal articles?

- GB023 Are there postnominal articles?

A "Yes" for GB020 and/or GB021 would seem to suggest a "Yes" for GB022 and/or GB023 and vice versa (the existence of articles presupposed they have a position, and if there are articles that have a position, it would suggest they are either definite/specific or indefinite). However, this is not a strict loop because there are articles that do not trigger a "Yes" for GB021 that can trigger a "Yes" for GB022 and/or GB023.

There are also two sets, outlined below, where it is impossible for a language to be coded as 0 for all features. It is not possible to have no word order whatsoever, and to not have at least one alignment system. For more on the specifics of this, see the feature documentation accompanying the dataset. Note that it is possible to have other value combinations, such as "1-1-1" or "0-0-?".

Transitive verb-order set

- GB131 Is a pragmatically unmarked constituent order verb-initial for transitive clauses?
- GB132 Is a pragmatically unmarked constituent order verb-medial for transitive clauses?
- GB133 Is a pragmatically unmarked constituent order verb-final for transitive clauses?

Alignment set

- GB408 Is there any accusative alignment of flagging?
- GB409 Is there any ergative alignment of flagging?
- GB410 Is there any neutral alignment of flagging?

Furthermore, besides the strict logical dependencies discussed so far there are other kinds of dependencies that are relevant for understanding languages. There is for example, as one of our anonymous reviewers pointed out, a likely historical connection between different elements all being pre-posed to the noun.

Given our extensive documentation of the features it is possible for users to identify such connections. One manner in which this can be addressed by users is by constructing new meta features that encompass and depend on our original features. For example, the three features below all concern marking of gender in the pronoun system:

- GB030 Is there a gender distinction in independent 3rd person pronouns?
- GB196 Is there a male/female distinction in 2nd person independent pronouns?
- GB197 Is there a male/female distinction in 1st person independent pronouns?

It may be interesting for a user to combine them to derive a feature asking "Is there a gender distinction in pronouns?".

Dependencies arising from language use and history are topics of ongoing research that the Grambank dataset can facilitate.

Example feature documentation

For each feature, we provide documentation that aims to aid the coders in applying the questionnaire consistently over the entire language sample. The features are described by each patron at our shared wiki (https://github.com/grambank/grambank/wiki), and this information is then found in the CLDF dataset in the Parameters table. Below is an example of the documentation provided.

Feature ID: GB028
Name: Is there a distinction between inclusive and exclusive?
Patron: Hannah J. Haynie
Summary:
Is there a pronoun or other marker that explicitly marks the inclusion of an interlocutor? This feature is not restricted to the pronominal system but includes person indexing as well. If inclusive is marked overtly in either the pronominal system or through verbal marking this is sufficient to trigger a 1 for this feature, even if exclusive has no overt morphological marking.

Procedure:
1. Code 1 if there is a pronoun or other marker, such as a person index, that explicitly marks the inclusion of an interlocutor in the first person plural.
2. Code 0 if the sections of the grammar discussing pronoun systems and person indexing on verbs describe no distinctions between inclusive and exclusive persons, and no pronominal forms or indices are found in examples glossed with grammatical information including INCL/EXCL or meanings such as 'you and I' or 'we all (not you)'. Pay close attention to the non-singular forms of first person pronouns and indices.
3. If you are uncertain whether some pronominal or index form(s) mark(s) a clusivity distinction (e.g. a form in a single example glossed 'you and I' that is known to encode dual number but is not clearly described regarding inclusivity, or multiple first person pronouns whose differences are not adequately described), code "?" and provide a brief comment describing the forms or descriptions that were unclear.

Examples

Southern Sierra Miwok (ISO 639-3: skd, Glottolog: sout2985)

Personal Pronominal Suffixes:

	**Series 1**	**Series 2**	**Series 3**	**Series 4**
1DU.INCL			*-ti:*	*-ti:*
1PL	*-tti-/-mahhi:*	*-me-*		
1PL.INCL			*-ticci:*	*-ticci:*

1PL.EXCL			*-mahhi:*	*-mahhi:*

(Broadbent 1964: 43)

Southern Sierra Miwok would be coded as 1. The lack of a first person dual exclusive form does not affect this designation, nor does the fact that the language has first person plural markers in Series 1 and 2 that do not mark clusivity.

Chalcatongo Mixtec (ISO 639-3: mig, Glottolog: sanm1295)

Pronouns

**PERS**	**GENDER**	**FREE**	**CLITIC**
1	Familiar	*rùʔù*	*=rí*
	Polite	*naʔa*	*=na*
	Inclusive (pl)	*žóʔó*	*=žó*

(Macaulay 1996: 81)

Chalcatongo Mixtec would be coded as 1. A plural pronoun that is unmarked for clusivity can be derived from the polite or familiar first person pronouns with a prefix, but the inclusive first person is inherently plural. There is no first person plural pro-form that is marked for exclusivity. The existence of an inclusive form is sufficient to trigger a 1 and the lack of an exclusive form has no impact on this.

Yongbei Zhuang (ISO 639-3: zyb, Glottolog: yong1276)

First person

**Singular**	**Plural (excl.)**	**Plural (incl.)**
ku	*tuo, po tu*	*lau'*

(Luo 2008: 327)

Yongbei Zhuang is coded as 1.

Grambank feature values

Individual structural features were formulated to take mainly binary (yes/no) values. This ensures a simple data structure, maximal clarity and interpretability of each datapoint, and a standard data format for the majority of the data. Six features have multistate values, each of which describes a particular word order or set of word orders that are available in that language. This makes it possible to identify situations where multiple word orders are possible without creating a logical dependency between features. They can be binarised, as seen in Materials and Methods: Data and Table S5.

Grambank departs from the traditions of many typological databases, like many chapters in WALS, in encoding whether a particular strategy for expressing a specific function is possible in a language, rather than stating what the single most common or dominant strategy is for expressing that function. The approach that Grambank uses aims to preserve valuable information about the spectrum of expressive possibilities in a language.

There are two types of missing data represented in Grambank. First, a response marked with a '?' denotes a datapoint where the source materials contain insufficient information for the coder to determine the value. A '?' response is accompanied by a reference to the source(s) consulted by the coder. A missing (empty) value represents a data-point for which no coder has made an attempt to code that particular feature for that language. There is thus a distinction in the data between values that have been checked, but could not be coded definitively at that time ('?') and values that are entirely missing for that feature/language combination. These two types of missing data in Grambank are different still from the 'not applicable' values used in some typological databases which is used to indicate that a particular feature is not relevant to a particular language because of another feature value. The formulation of Grambank questions removes the 'not applicable' distinction and the absence of a phenomenon is simply coded as '0' (absent) in this dataset.

Grambank data collection

The primary sources used in Grambank are published descriptions of grammatical structures. There are over 7,000 languages found around the world, and of these, approximately 60% are described by a grammar or a grammar sketch (*1*). Data for Grambank were also obtained by consulting linguists with expertise on particular languages; see acknowledgements for a list of experts who have shared their knowledge.

The coding workflow and support structures employed by Grambank were designed to minimize any potential data compatibility and consistency issues that may arise from the diversity of source materials considered. The questionnaire is adapted to being answerable to a standard level given a grammar sketch, and coders were provided with continuous support for discussing and evaluating possible interpretations of the data. Differences in the quality of linguistic descriptions across languages and the existence of competing analyses impacts the completeness of data for individual languages, but should have minimal impact on coding decisions.

Data were entered by research assistants and language specialists who filled in the Grambank questionnaire using available grammars and provided references for each datapoint, as well as comments if appropriate. Coders were trained to fill in the questionnaire by local supervisors who were involved in the design and ongoing curation of Grambank features. Training included

coding a previously coded language, detailed supervisor-led discussion of each questionnaire feature, introduction to the project's documentation and discussion forum, and examination of previous discussions and complicated coding decisions. A key feature of the Grambank coding process was that each feature had one or a pair of feature experts – known project-internally as "patrons" – who adjudicated complicated coding situations where agreement cannot be reached in discussions between the local supervisor and individual coders. In cases where there was doubt or disagreement about specific coding decisions, the patron made the final judgment. Documentation of each feature can be found in our GitHub repository's wiki (https://github.com/grambank/grambank/wiki). In this way we ensure consistency across coders and provide a rich documentation of the decisions required to convert the complexity of a grammatical description into a large-scale digital database in a transparent and reproducible manner.

Grammars often do not explicitly state whether a particular phenomenon is absent. Coders therefore have to inspect not only the text, but also the available language examples in order to make informed judgments about the values of features. In some cases it is difficult to judge whether no mention of a feature in the available grammar(s) is evidence that the phenomenon itself is absent in the language, or simply an oversight or omission by the author. The coder judges this by how extensive the description of that grammatical domain is in the grammar (e.g. it can typically be assumed that definite articles are absent if they are not mentioned in a section on the noun phrase). In cases where there was uncertainty and it could not be resolved with more examination of the sources and discussion, the relevant feature was coded as '?' for that language.

SM1:2 Technical validation

An inter-coder reliability study was conducted early on to assess the quality of the curated Grambank data. 20 languages were randomly selected from the set of 4,338 languages with a grammar or grammar sketch. For each of the 20 languages, three out of six members of the Grambank design team were randomly selected to code the language independently of each other. They were each given the same instructions, the same deadline, the same preparatory and auxiliary materials and the same source documents describing the language in question. In this way, a total of 8,311 data-points were collected, which allowed for 7,876 pairwise comparisons.

Coders disagreed most often on the basic issue of whether there is enough information to assign a specific value for a particular feature: in 25% (1996/7876) of the comparisons one of the coder assigned a '?' and the other a specific value. In 20% (1557/7876) of the comparisons both coders agree on a '?', i.e., that there is insufficient information for concluding a specific value. When both coders assigned a specific value for the language, however, they agreed on the value 87% of the time (3753/4323). This number rises to 90% if only datapoints based on the same grammatical description are compared. While pairwise comparisons are simple to interpret, they are not controlled for number of raters and chance agreement. Fleiss' Kappa (*68*) calculates the measure of agreement over chance, which in this study is 0.72. While there are no widely established standards of significance for Fleiss' Kappa, guidelines (*69*) classify this score as "substantial agreement".

As the bulk of the coded data in Grambank has been collected by research assistants and the above inter-rater reliability study involved members of the design team rather than these research assistant coders, one may legitimately ask whether the results generalize from experts to research assistants. While no controlled study was used to answer this question, there were cases of unplanned double-coding. Among these double-coded languages, there were two languages that also featured in the inter-coder reliability study above. These can provide a general measure of how research assistant coding compares to expert coding. The levels of agreement when comparing research assistants with other research assistants (78%, 79%, 87%, 91%, 91%), research assistant vs. expert (87%, 89%, 95%, 96%), and expert vs. expert (87% as above) do not differ appreciably. The reason for this may be that time and devotion to the task makes up for the difference in expertise. Few other figures on reliability of typological databases are available for comparison. However, an accuracy rate of 87% is similar to rates for a select few well known languages in WALS (*70*) and Jazyki Mira (*71*). Hence, this may be the natural margin of error associated with human factors and the level of abstraction of typological features.

SM1:3 Web interface

The latest released version of the Grambank database is available for interactive browsing at https://grambank.clld.org under a Creative Commons 4.0 Attribution license. It is served by a web application built with the toolkit developed for the Cross-Linguistic Linked Data project (*72*). Consequently it inherits the core database schema common to all CLLD applications, which includes standard data types for common entities such as:

- *contribution:* a citable sub-unit of a dataset
- *language:* an instance of the main subject of study
- *parameter:* a measurable factor which can be compared across languages -- a *feature* in Grambank
- *value:* a measurement, i.e. a value determined for a particular language and a given parameter
- *source:* a bibliographical record describing the source of a value

The CLLD framework also provides tools for basic analysis and visualization of underlying data. The Grambank website integrates these tools into interfaces for accessing data by feature or by language, with further pages that summarize data by other fields (e.g. language family, source). The Languages page also presents an interactive mapping tool, as well as a table of coded languages that can be searched by ID, language name, or latitude/longitude. The Features page of the website presents a list of features in tabular form, and can be filtered by ID, name, morphosyntactic unit, form, or grammatical function. Linked pages for individual features provide further information about the feature, data values in tabular format, and an interactive tool that enables map visualization of feature value distributions. Additional filters allow users to sort languages by families and macroareas.

SM1:4 Accessing Data

The Grambank data are archived with Zenodo as a Cross Linguistic Data Format (CLDF) structure dataset (*64*). Because the CLDF format is essentially a set of CSV files, it is simple to access the data from a wide variety of computing environments. Unzipping a download of the whole of Grambank CLDF dataset will result in a directory with the following contents:

- StructureDataset-metadata.json: The machine readable description of the dataset
- values.csv: The main data file, containing all codings
- languages.csv: A CSV file with additional metadata about the coded languages
- parameters.csv: A CSV file with metadata about the coded features.
- sources.bib: A BibTeX file containing bibliographic metadata about the sources used for Grambank coding.

Methods for accessing and using this data in environments such as Python, SQL, R, and with off-the-shelf CSV tools are described in detail at the GitHub repository of the CLDF dataset.

- Zenodo location for Grambank v1.0: https://doi.org/10.5281/zenodo.7740140
- Zenodo location for scripts associated with this paper: https://doi.org/10.5281/zenodo.7740822

SM1:5 License and referencing

Grambank is released under a Creative Commons 4.0 (CC-BY) license. Any user may share and adapt the data, as long as they give appropriate credit by citing this paper and the relevant version of the database. Languages are still being added to Grambank and the project welcomes feedback from experts, which may result in additions or changes in the coding of languages. The web publication of Grambank will be updated regularly with new releases; therefore users should reference the Grambank data they use by its specific release version and download date. The first version is 1.0 and should hence be referenced as "Grambank 1.0", this is the dataset that is presented in this paper and consists of 2,467 languoids (languages, dialects and proto-languages).

SM2 Supplementary figures

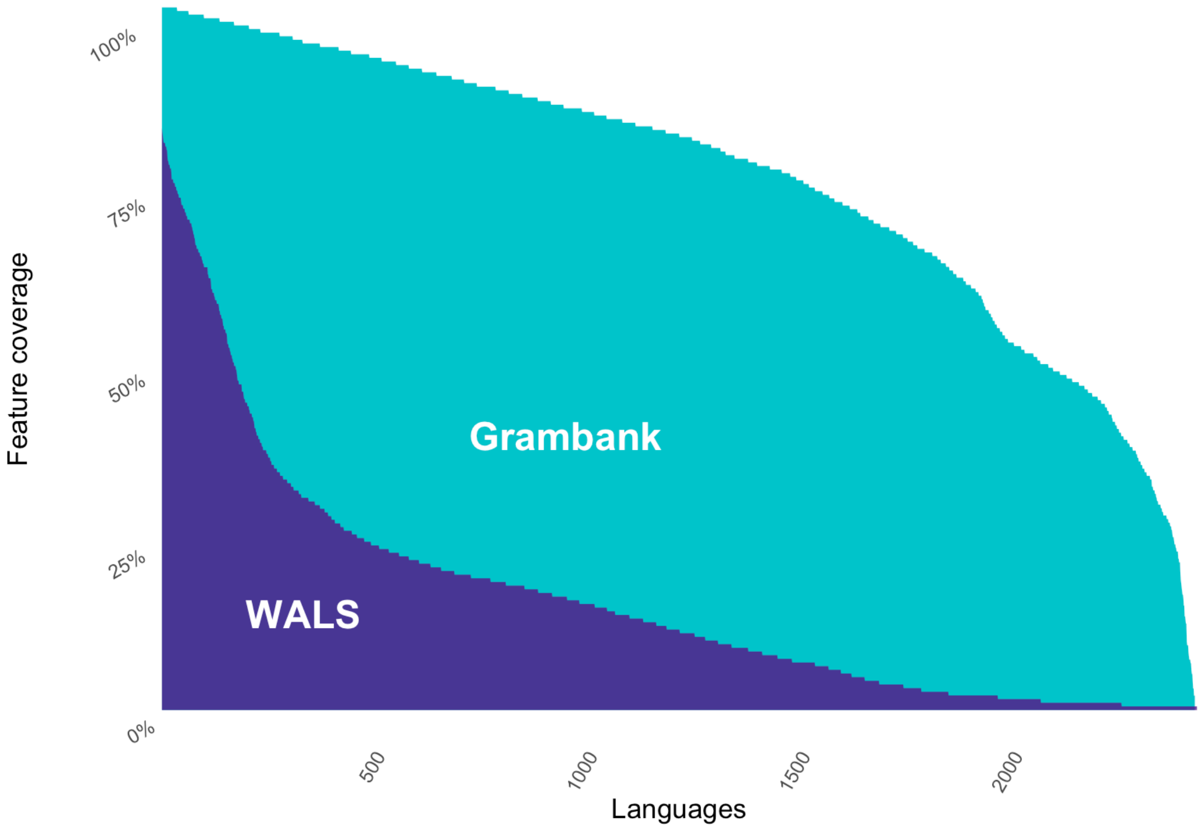

Figure S1. Comparison of coverage per language and feature in WALS and Grambank. This plot shows that the amount of missing data per language is much lower in Grambank compared to WALS. The total number of languages is 2,430 for Grambank and 2,435 for WALS. The numbers are derived on the dialect-aggregated dataset, see Materials and Methods: Data.

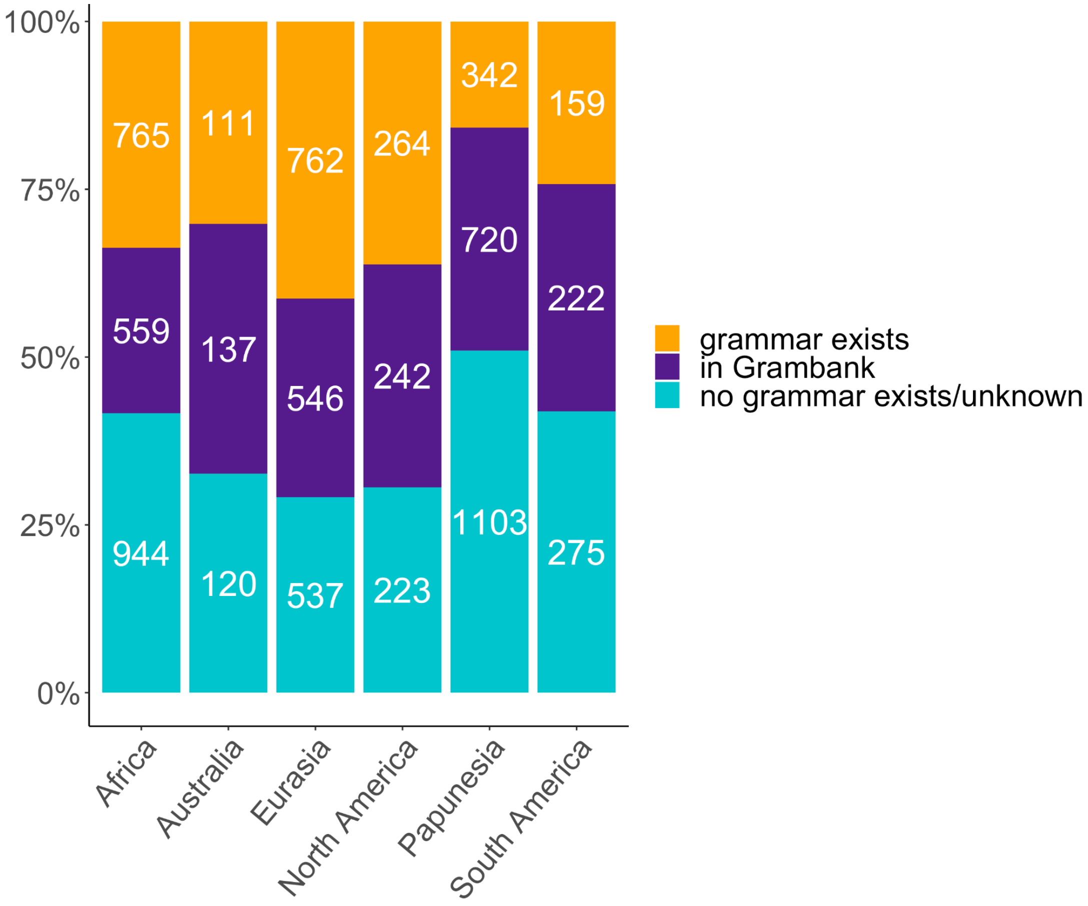

Figure S2. Barplot showing the coverage of languages per Glottolog macroarea. Light blue represents languages which do not yet have a grammar as indexed by Glottolog, dark blue indicates languages that are already in the Grambank database and orange denotes languages which have a grammar indexed in Glottolog but which are not (yet) in the Grambank dataset. Languages in the light blue category are most likely not possible to include in Grambank, whereas the orange category could be included in future. The numbers are derived from the dialect-aggregated dataset, see Materials and Methods: Data.

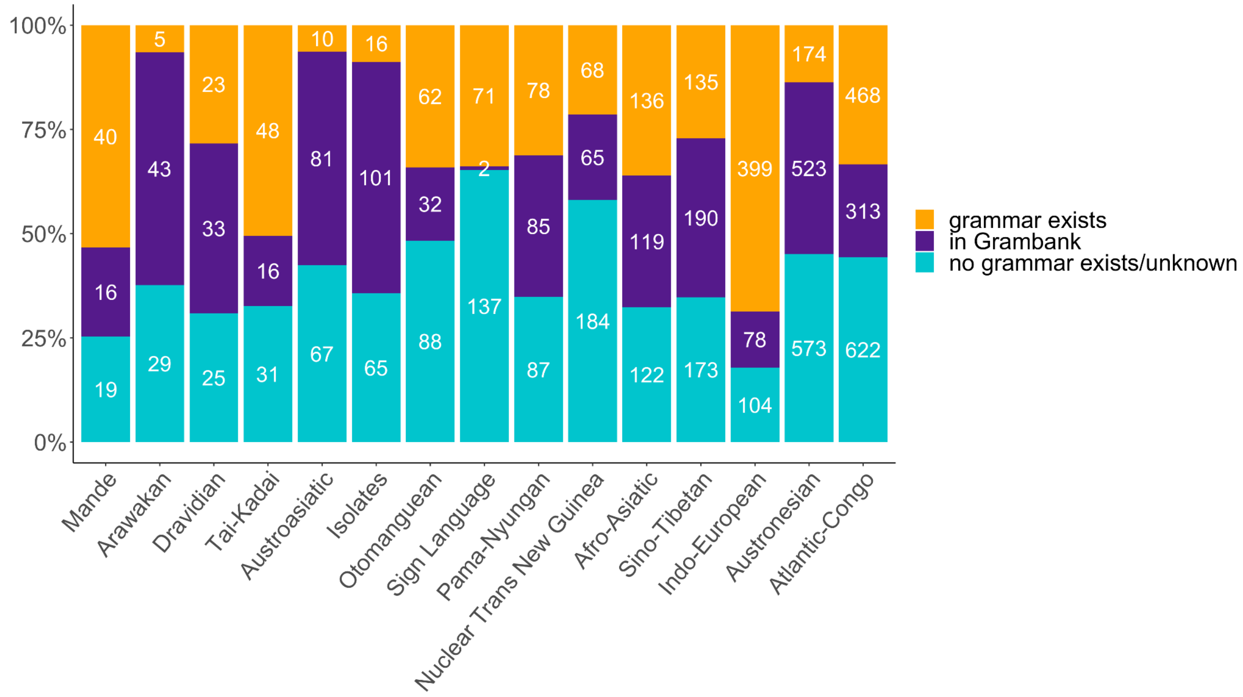

Figure S3. Barplot showing the coverage of languages in the 15 largest language families. Light blue represents languages which do not yet have a grammar as indexed by Glottolog, dark blue indicates languages that are already in the Grambank database and orange denotes languages which have a grammar indexed in Glottolog but which are not (yet) in the Grambank dataset. Languages in the light blue category are most likely not possible to include in Grambank, whereas the orange category could be included in future. The numbers are derived from the dialect-aggregated dataset, see Materials and Methods: Data.

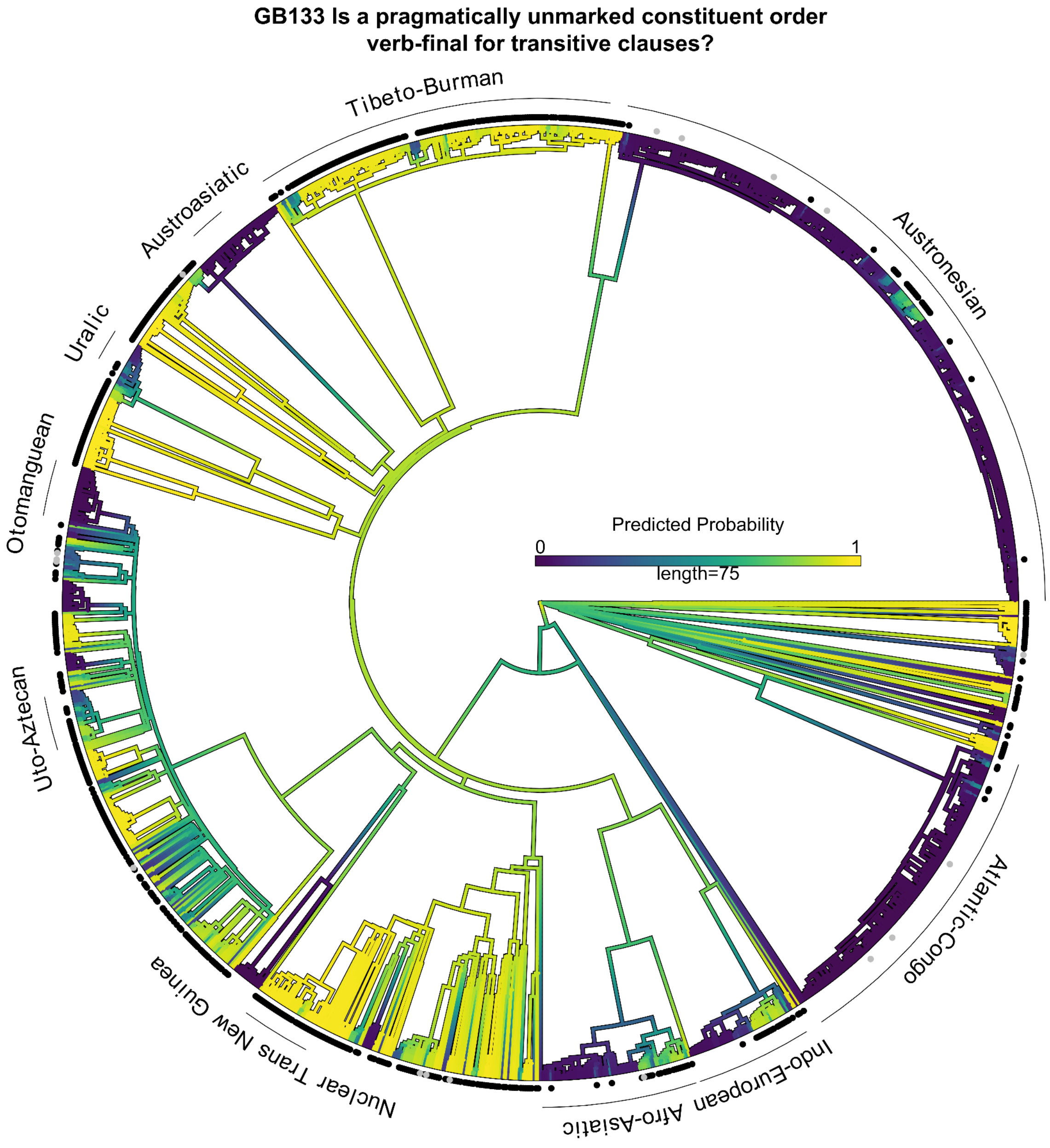

Figure S4. Tree plot of GB133, the Grambank feature with the highest phylogenetic effect in the INLA (dual) model. Tip point colors represent observed values: black = yes (verb-final is a pragmatically unmarked constituent order for transitive clauses), uncolored = no (verb-final is *not* a pragmatically unmarked constituent order for transitive clauses), gray = missing data. Branch colors represent probability estimates: yellow = higher probability that verb-final is a pragmatically unmarked constituent order for transitive clauses, purple = lower probability that verb-final is a pragmatically unmarked constituent order for transitive clauses.

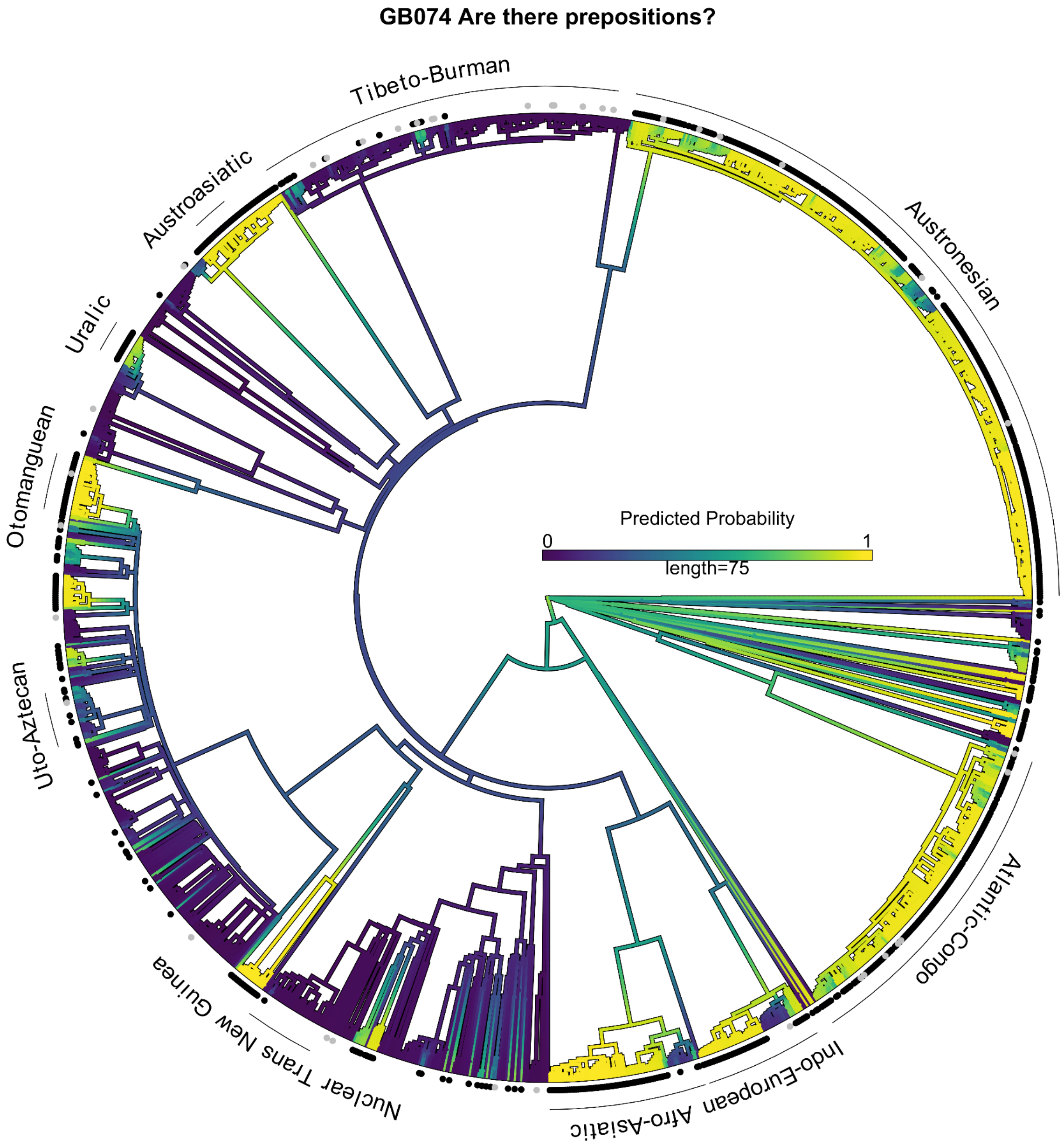

Figure S5. Tree plot of GB074, the Grambank feature with the second highest phylogenetic effect in the INLA (dual) model. Tip point colors represent observed values: black = yes (there are prepositions), uncolored = no (there are *not* prepositions), gray = missing data. Branch colors represent probability estimates: yellow = higher probability that there are prepositions, purple = lower probability that there are prepositions.

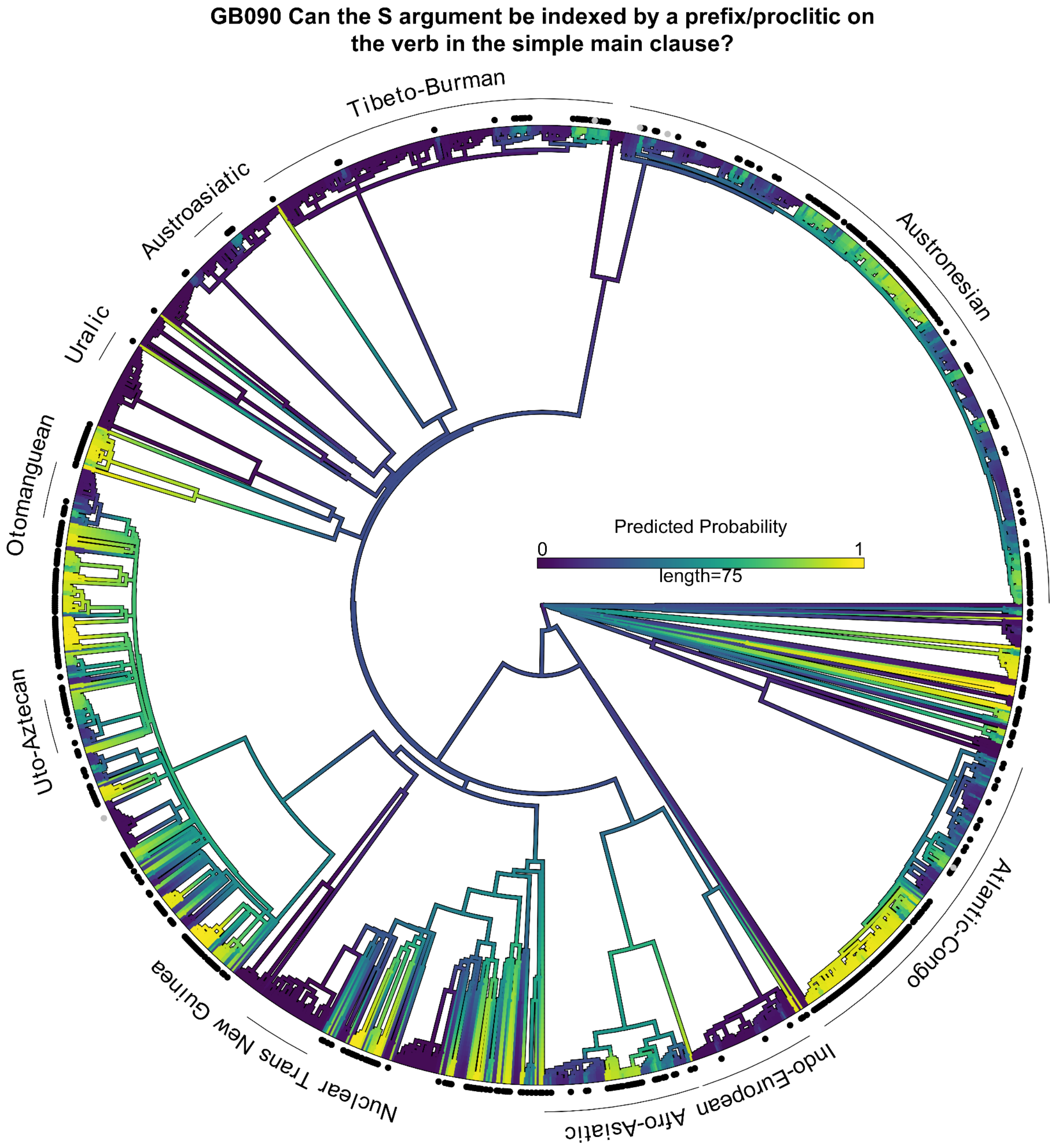

Figure S6. Tree plot of GB090, the Grambank feature with the third highest phylogenetic effect in the INLA (dual) model. Tip point colors represent observed values: black = yes (the S argument can be indexed by a prefix or proclitic on the verb in simple main clauses), uncolored = no (the S argument can *not* be indexed by a prefix or proclitic on the verb in simple main clauses), gray = missing data. Branch colors represent probability estimates: yellow = greater probability that the S argument can be indexed by a prefix or proclitic on the verb in simple main clauses, purple = lower probability that the S argument can be indexed in this way.

GB038 Are there demonstrative classifiers?

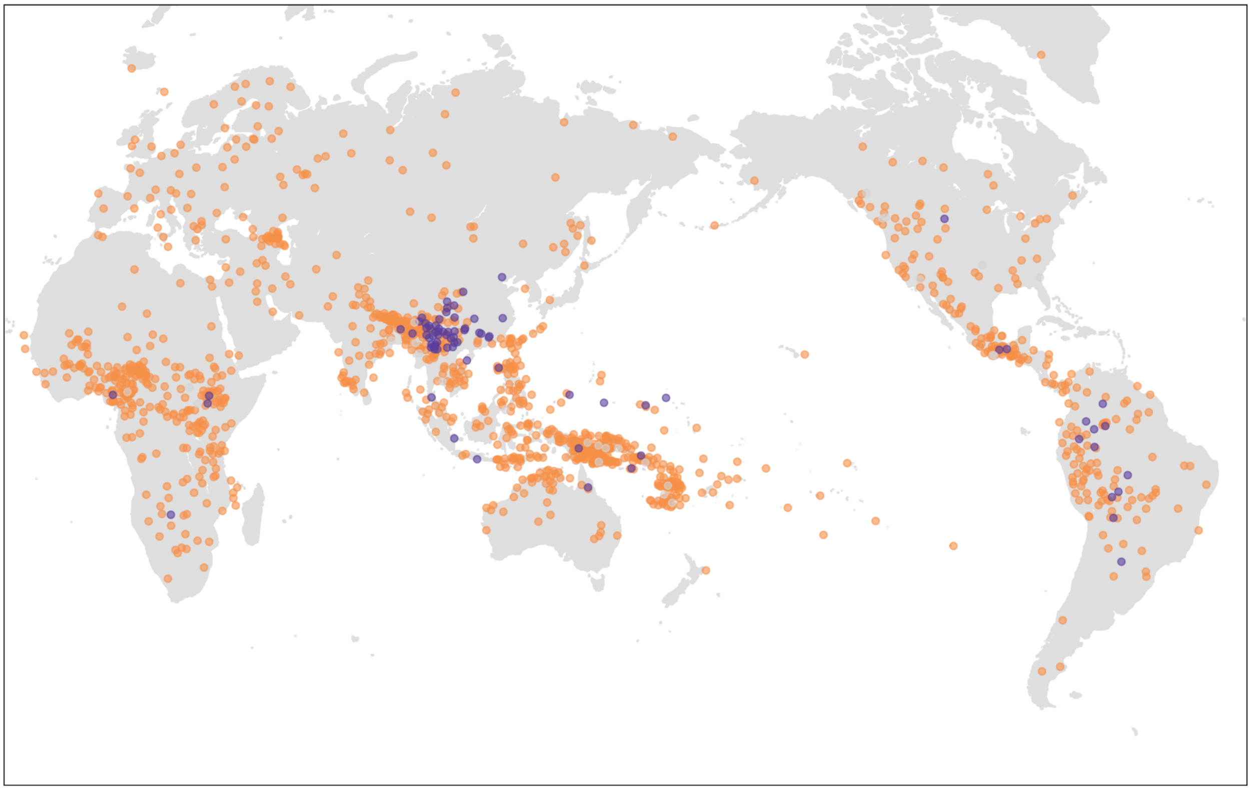

Figure S7. Map of GB038, the Grambank feature with the highest spatial effect in the INLA (dual) model. Purple indicates languages that have demonstrative classifiers; Orange indicates languages that do *not* have demonstrative classifiers.

GB080 Do verbs have suffixes/enclitics, other than those that only mark A, S or P (do include portmanteau: A & S + TAM)?

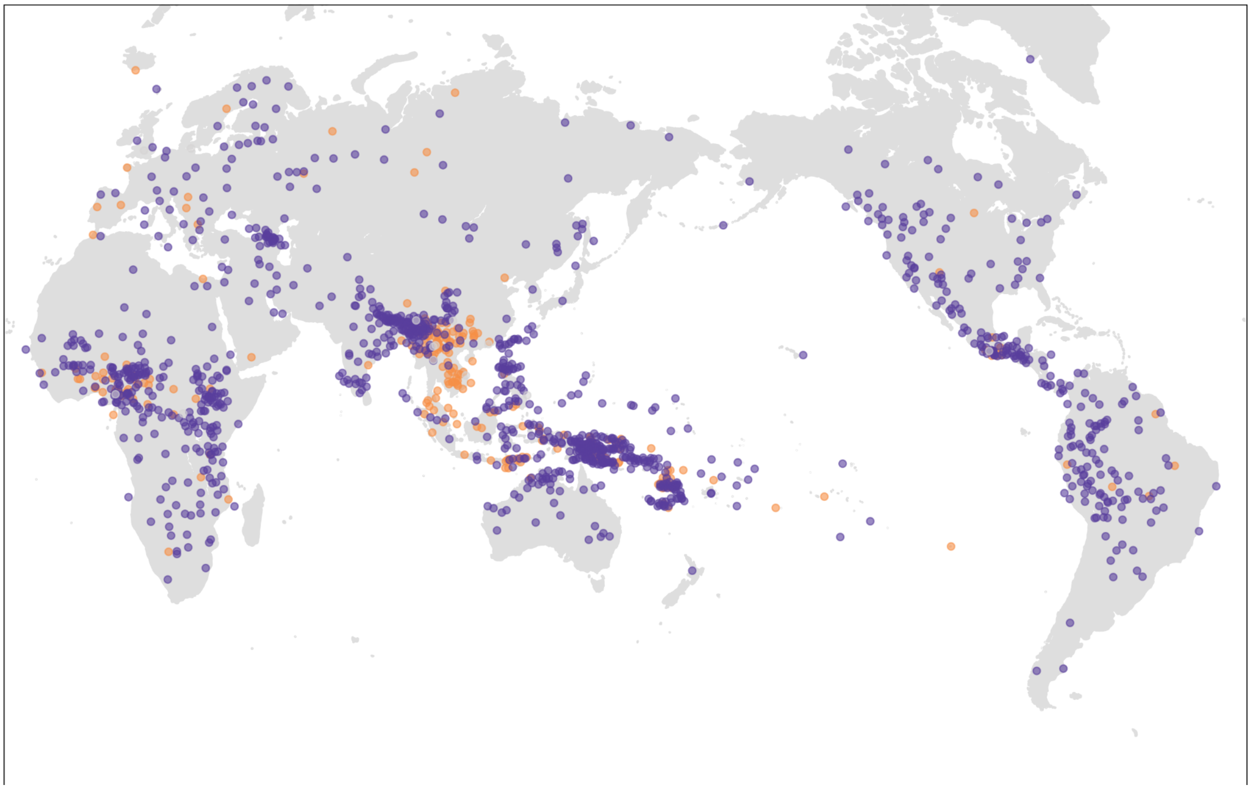

Figure S8. Map of GB080, the Grambank feature with the second highest spatial effect in the INLA (dual) model. Purple indicates languages that have suffixes or enclitics that encode information other than the categories listed in the feature; Orange indicates languages that do *not* have such suffixes or enclitics.

GB136 Is the order of core argument (i.e. S/A/P) constituents fixed?

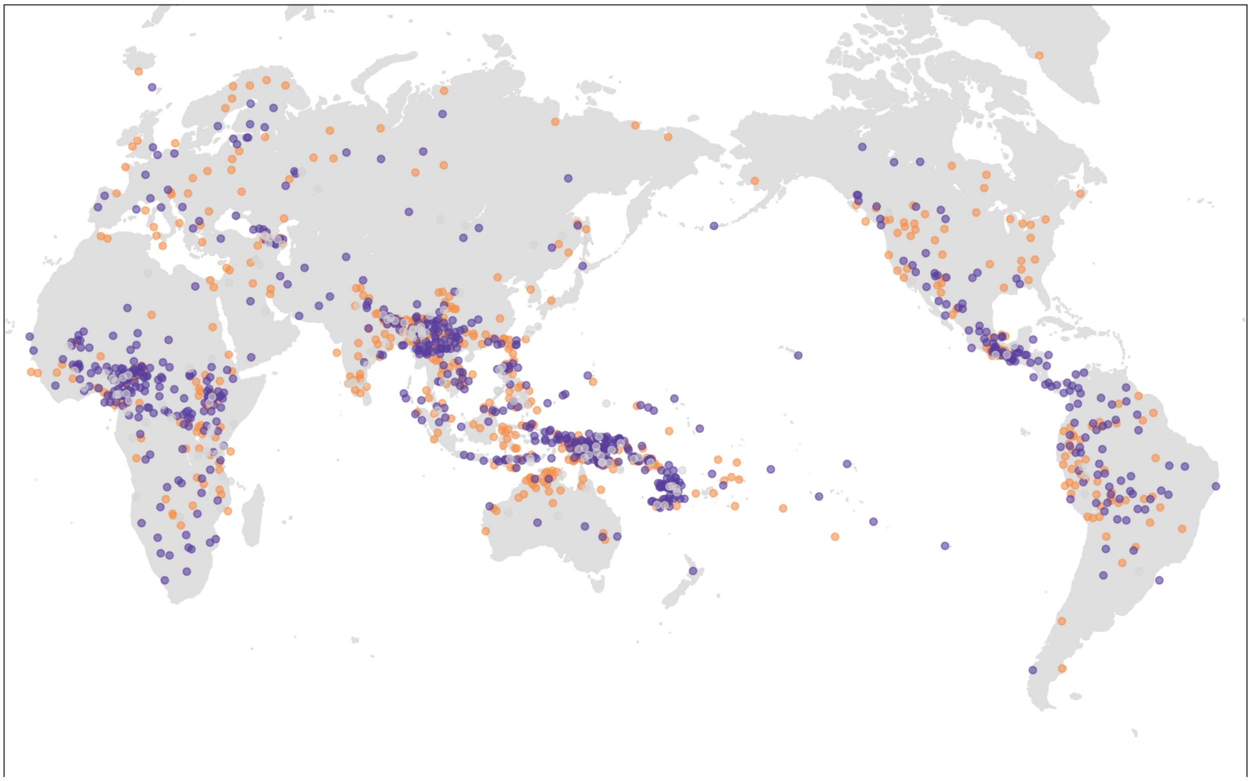

Figure S9. Map of GB136, the Grambank feature with the third highest spatial effect in the INLA (dual) model. Purple indicates that fixed word order occurs in the language; Orange indicates that fixed word order does *not* occur in the language.

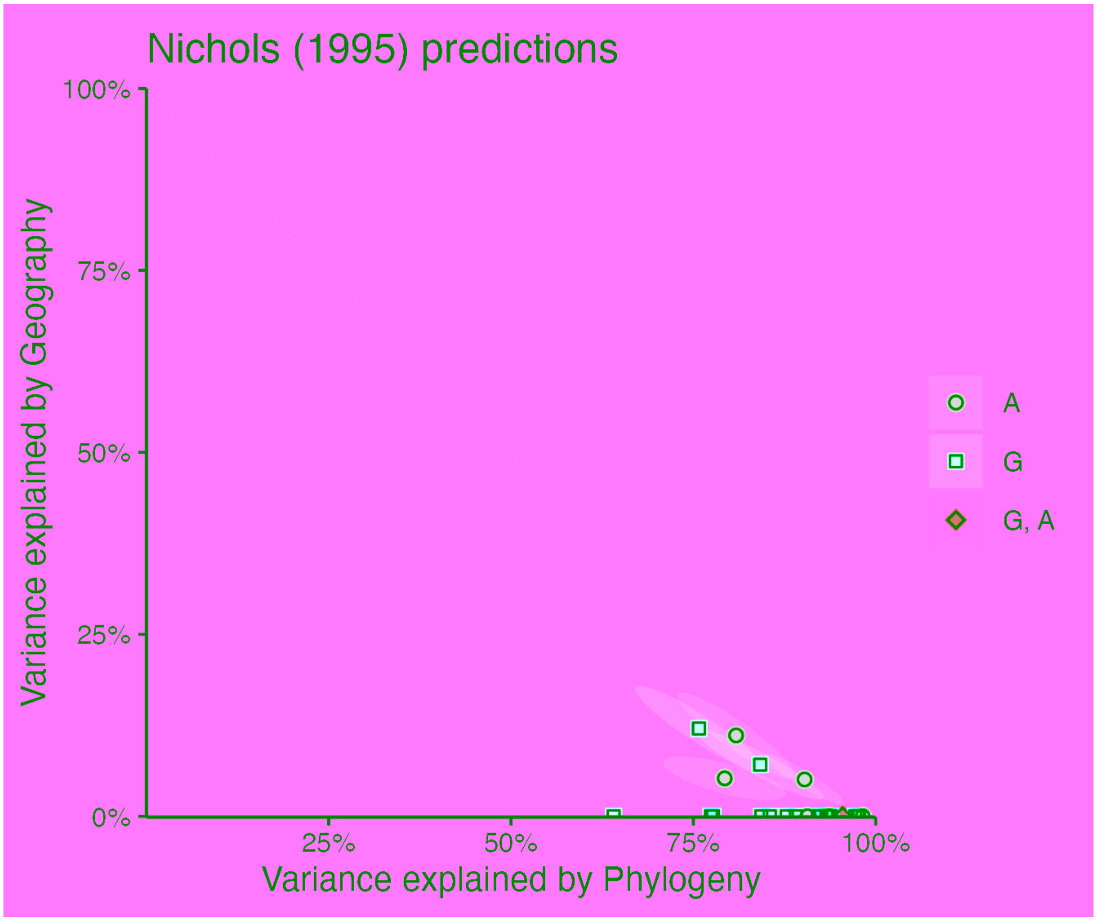

Figure S10. Scatterplot of the phylogenetic (x-axis) and spatial effects (y-axis) for features included in Nichols (1995). The points are colored for the prediction by Nichols: A = Areal, G = Genetic and G, A = Both. The term *genetic* here is used by Nichols **(*20*)** in a similar/identical fashion to how we have used *phylogenetic* in this paper.

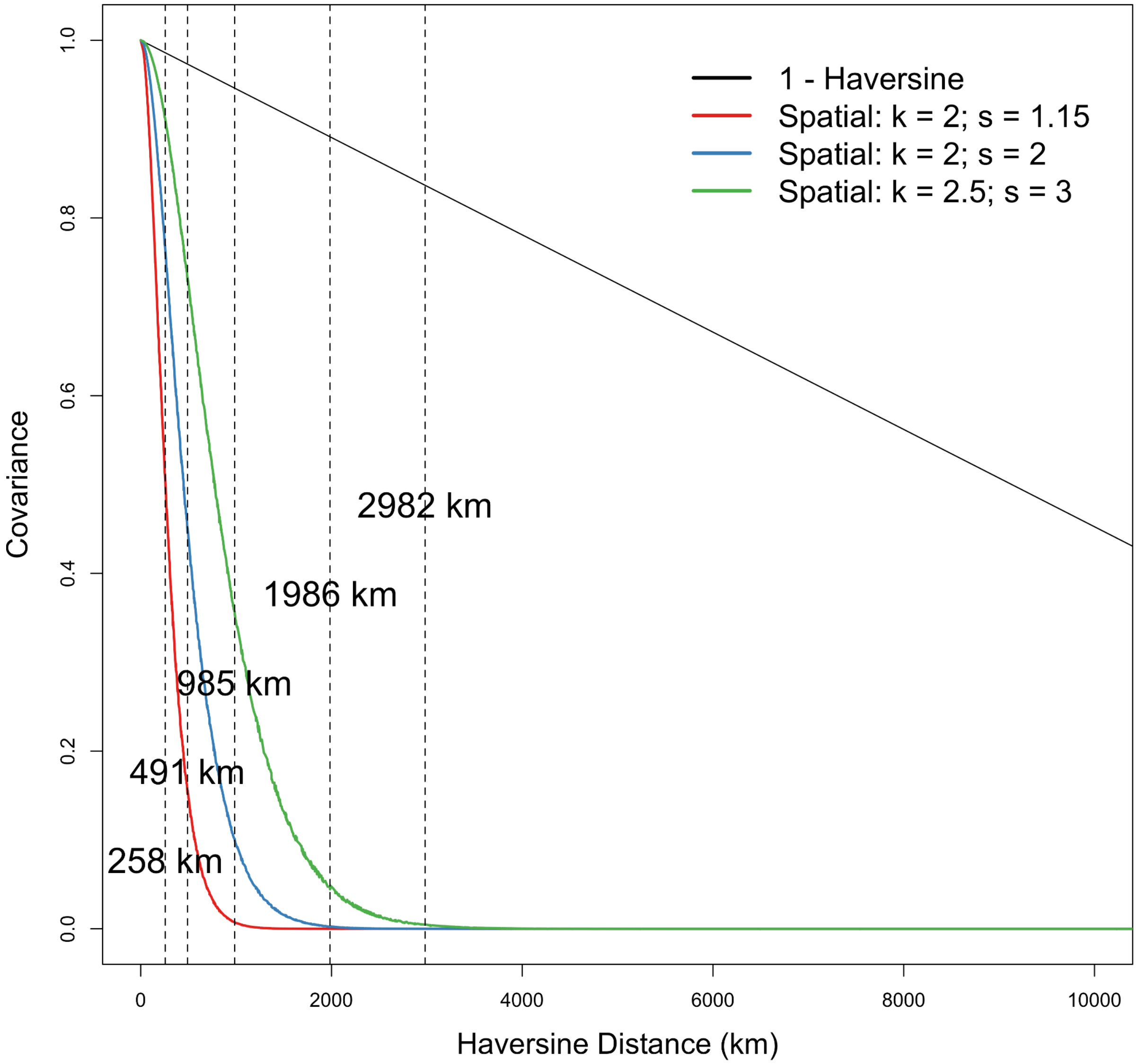

Figure S11. Spatial decay in precision matrices for spatiophylogenetic analysis. This figure shows the relative decay in covariance based on the various parameterisations of the Matérn function. The x-axis shows Haversine distance ("as-the-crow-flies" distances, taking into account the curvature of the earth), and is shown on the y-axis with the black line for reference. The red line indicates the parameterization of spatial covariance used in the main text. Blue and green lines show parameterizations that iteratively increase the relationship of geography between languages in the model. Vertical dotted lines ground the covariance functions in real-world distances to give a sense of at what point geographic relationships are no longer statistically relevant in this model.

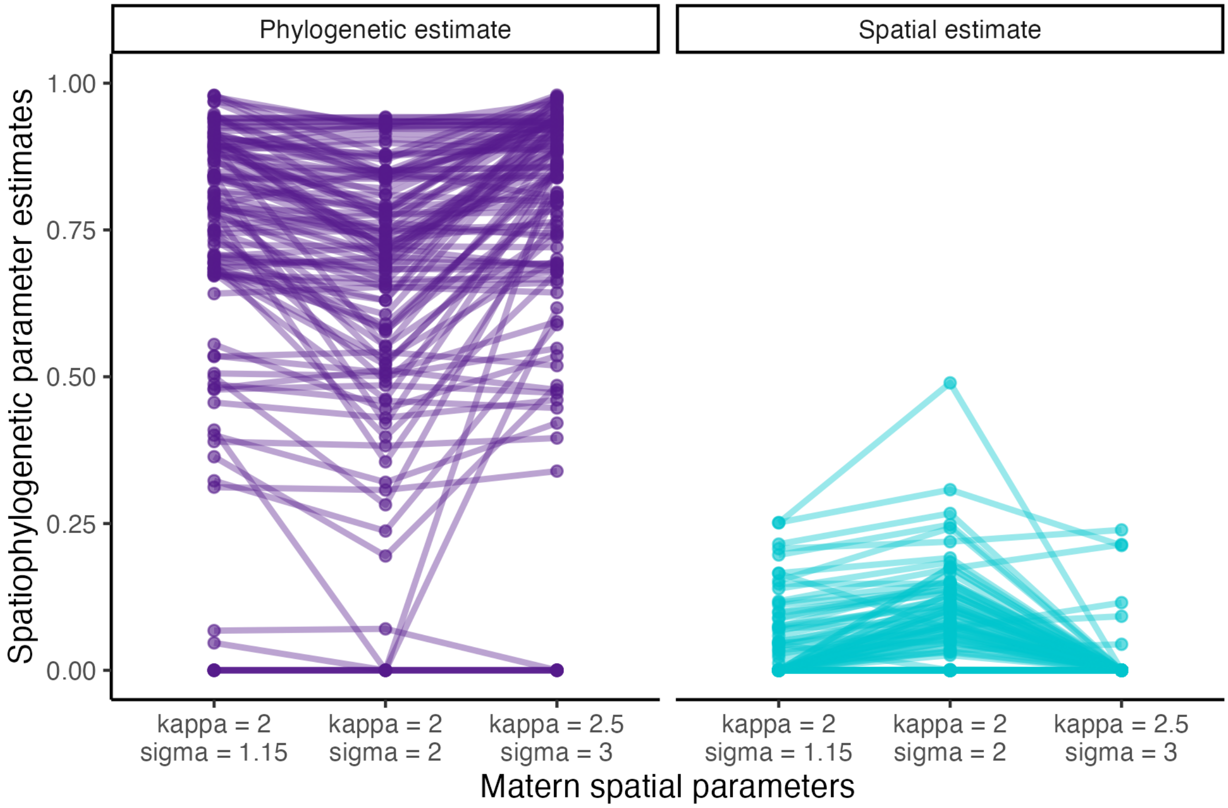

Figure S12. Spatiophylogenetic parameter estimates for the effect of language (left) or geography (right) when varying the Matérn spatial decay parameter. Decay functions cause the spatial influence of languages to be effectively zero at approximately 1000km, 2000km, and 3000km moving from left to right on the x axis. Increasing the influence of spatial effect generally has little influence on the conclusions drawn.

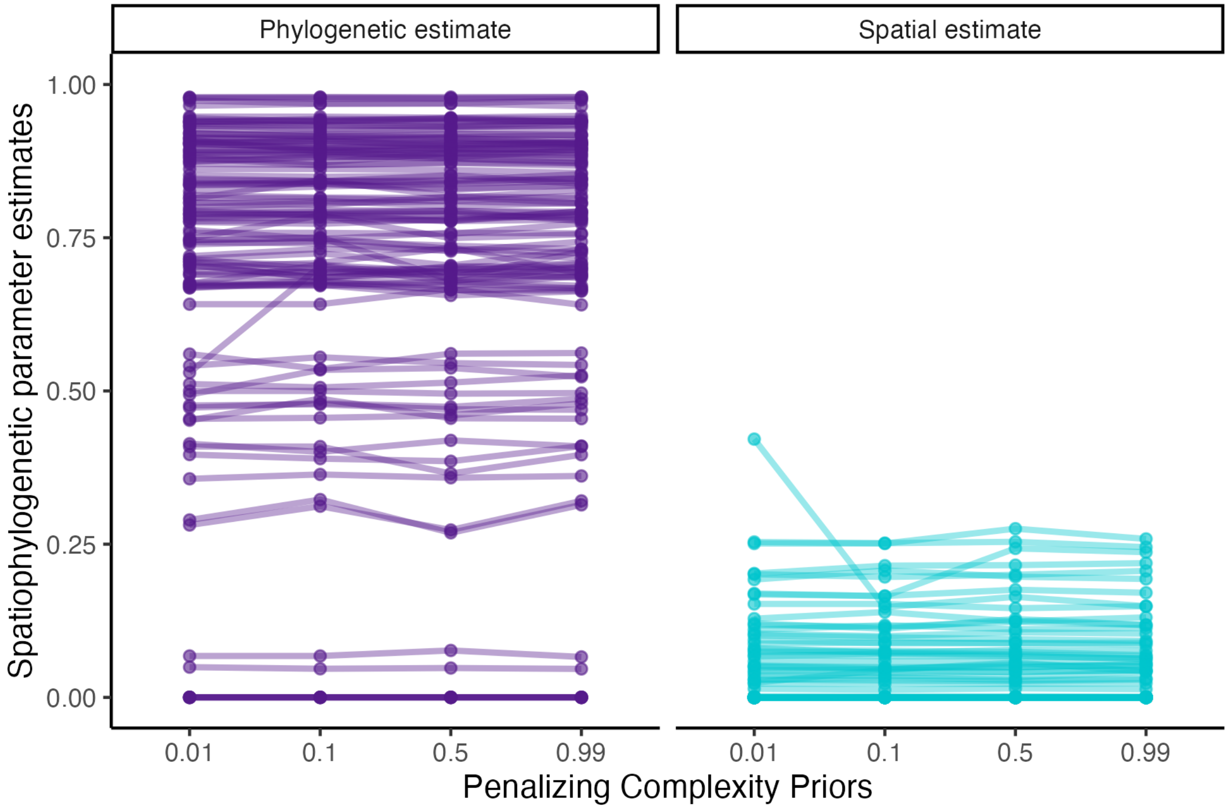

Figure S13: Varying Priors for Penalizing Complexity in the INLA-analysis.

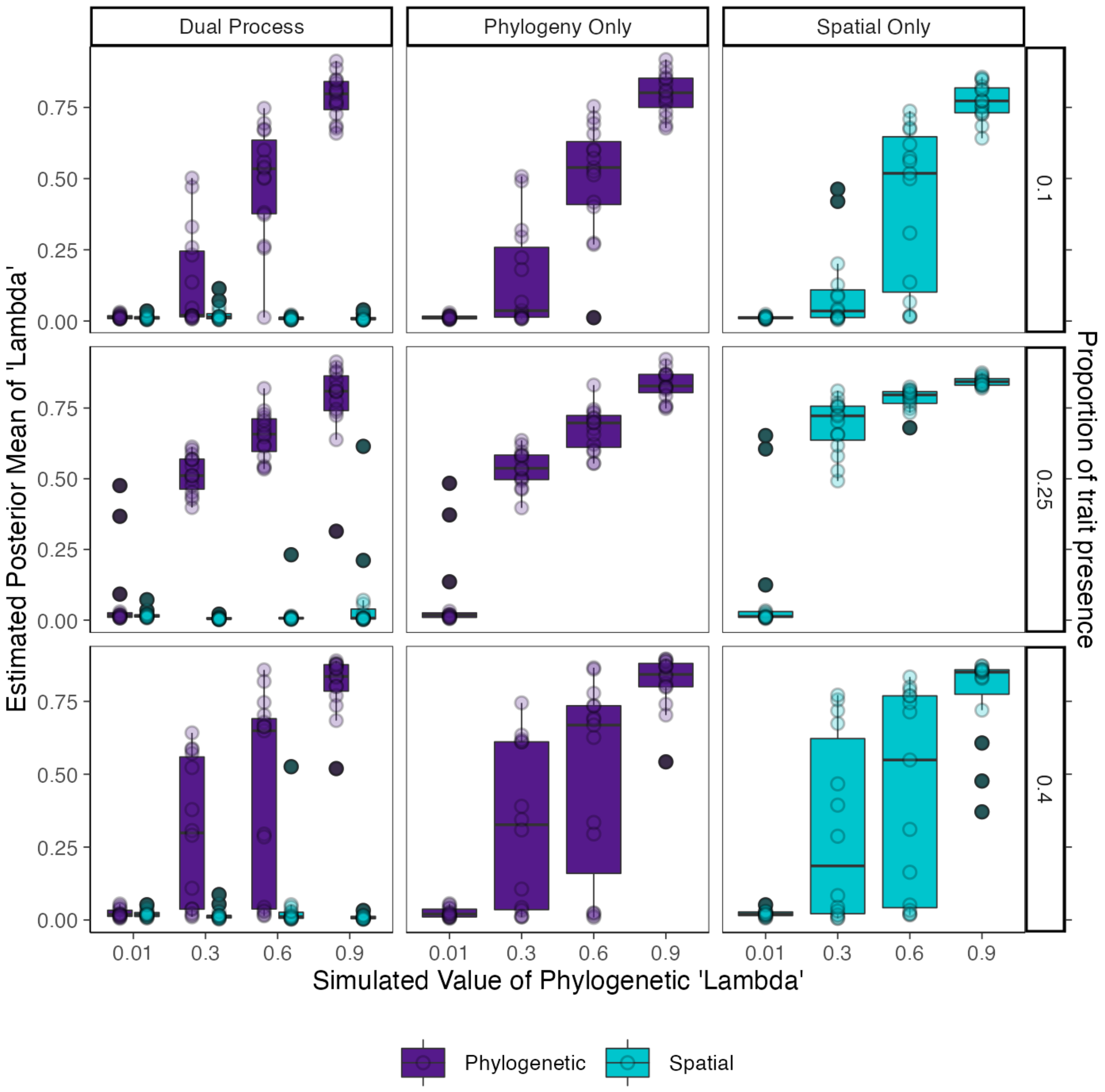

Figure S14. Simulation results for the 12 conditions (four levels of phylogenetic signal, for three different proportions of traits). Each column of graphs contains the results for a particular model structure, and each row of graphs contains the results for a particular proportion of traits. Each graphs shows the results across the four levels of phylogenetic signal The dual process model contains two boxplots per level of phylogenetic signal, one representing the posterior mean for the phylogenetic effect, and one the posterior mean of the spatial effect.

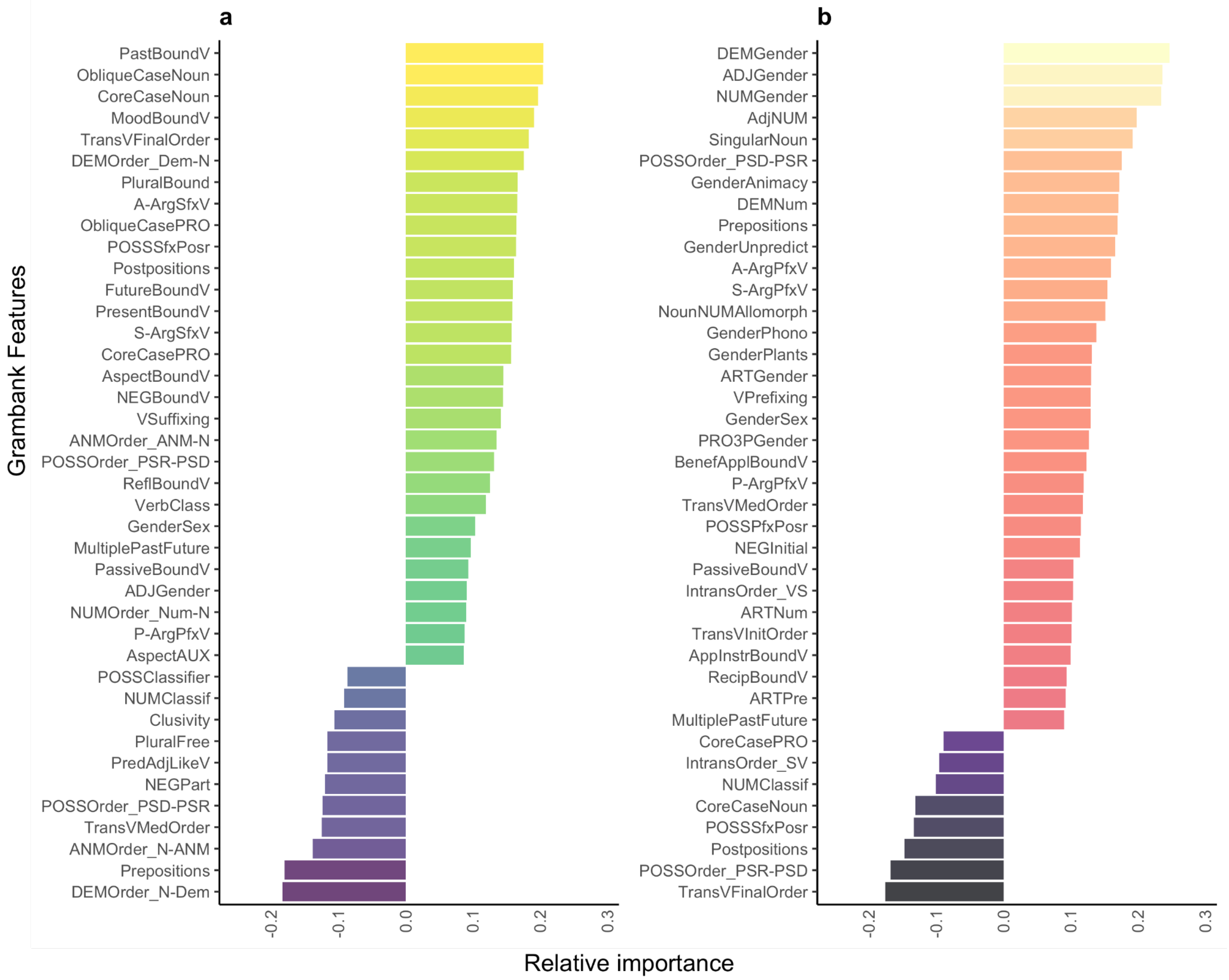

Figure S15. Feature loadings onto PC1 and PC2, including only the top 40 most contributing features.

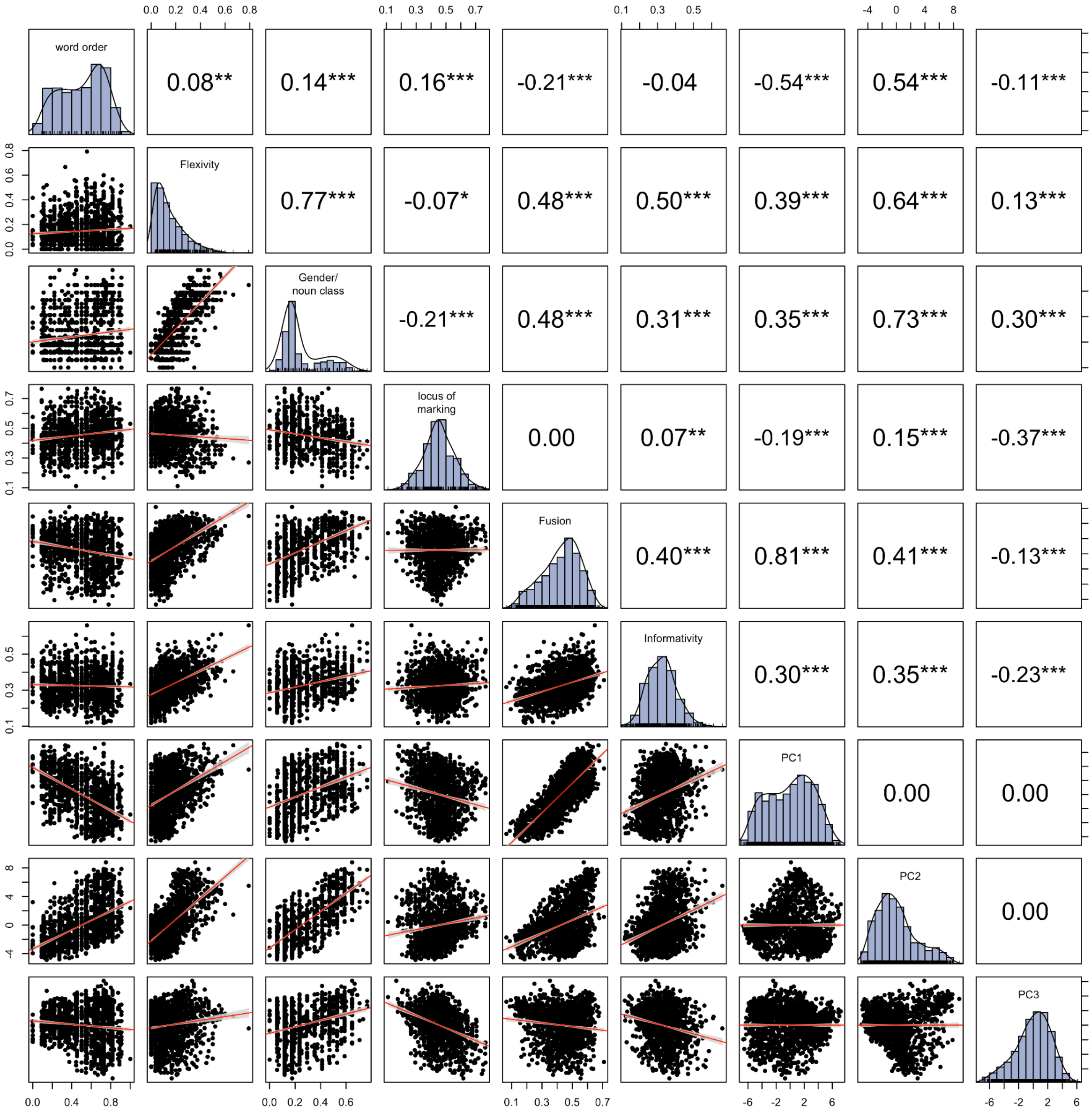

Figure S16. Scatterplot matrix showing the Pearson correlations between the first three principal components of the data and the theoretical metrics.

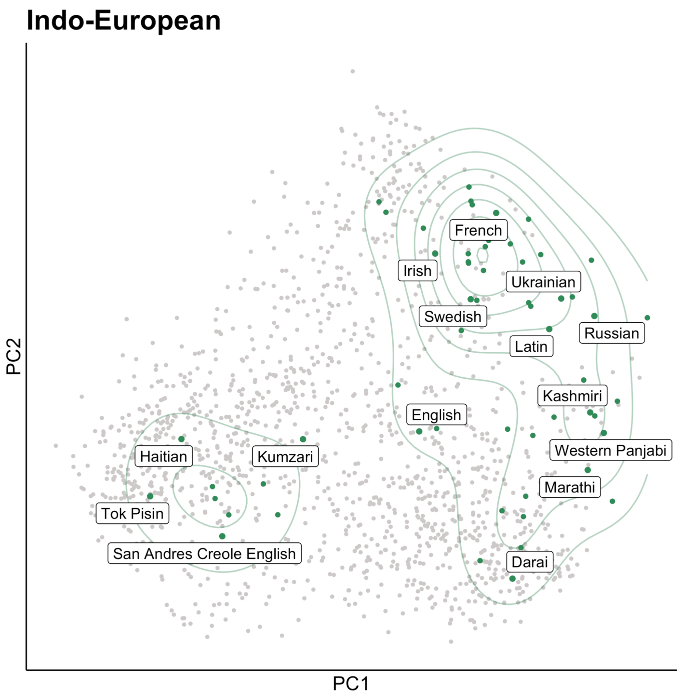

Figure S17. Scatterplot of Indo-European languages (green) among all other languages (grey) and their position given PC1 and PC2 with specific languages highlighted with names.

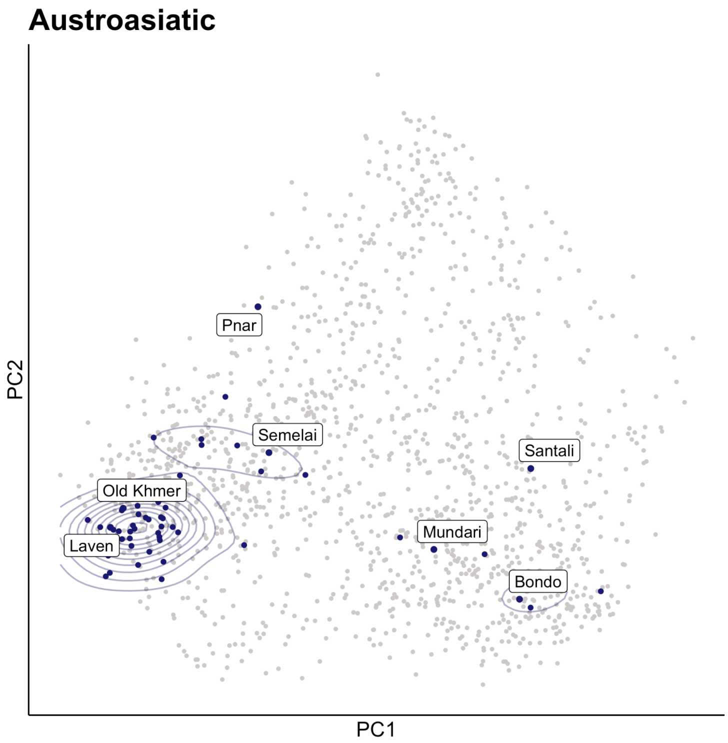

Figure S18. Scatterplot of Austroasiatic languages (blue) among all other languages (gray) and their position given PC1 and PC2 with specific languages highlighted with names. The two major clusters in the Austroasiatic family correspond to languages inside and outside of the Indian subcontinent.

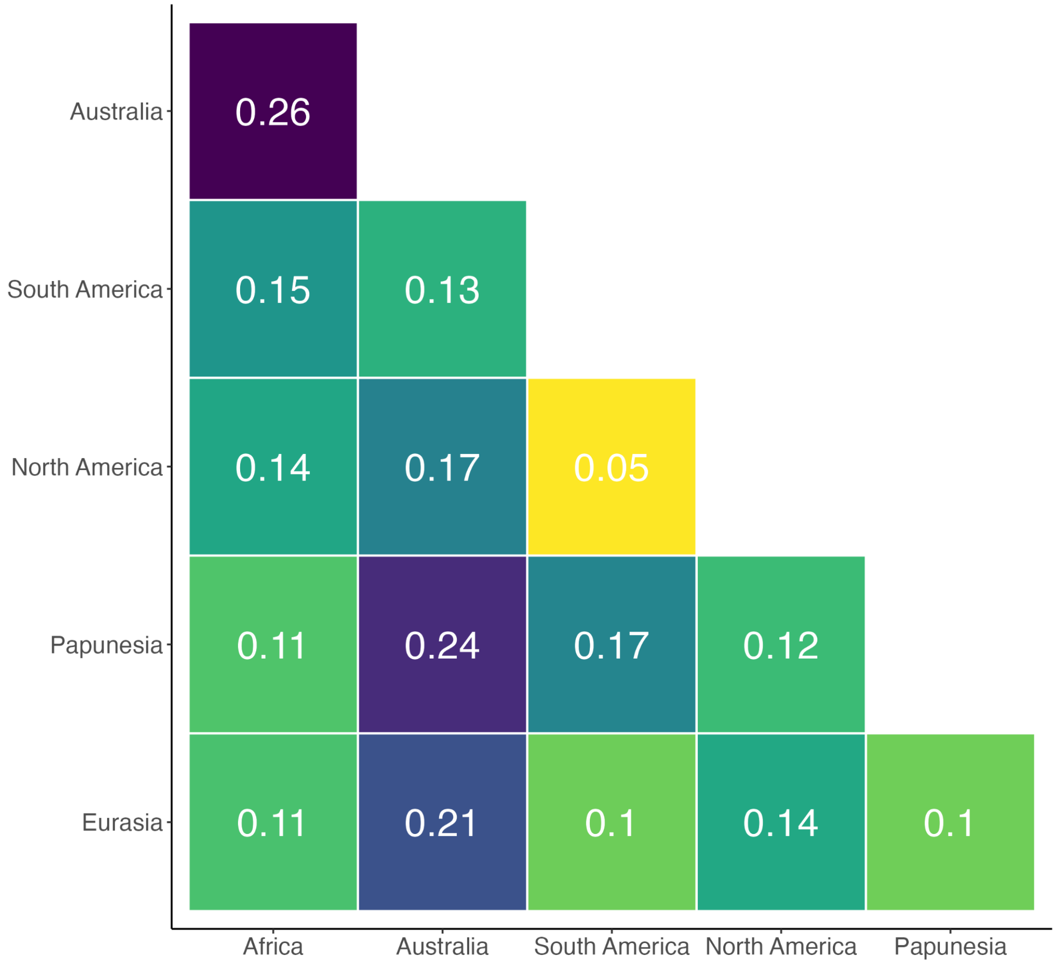

Figure S19. Pairwise Cultural Fixation scores over macroareas in the Grambank dataset. The pair with the lowest score (and therefore most likely to be similar) is North and South America.

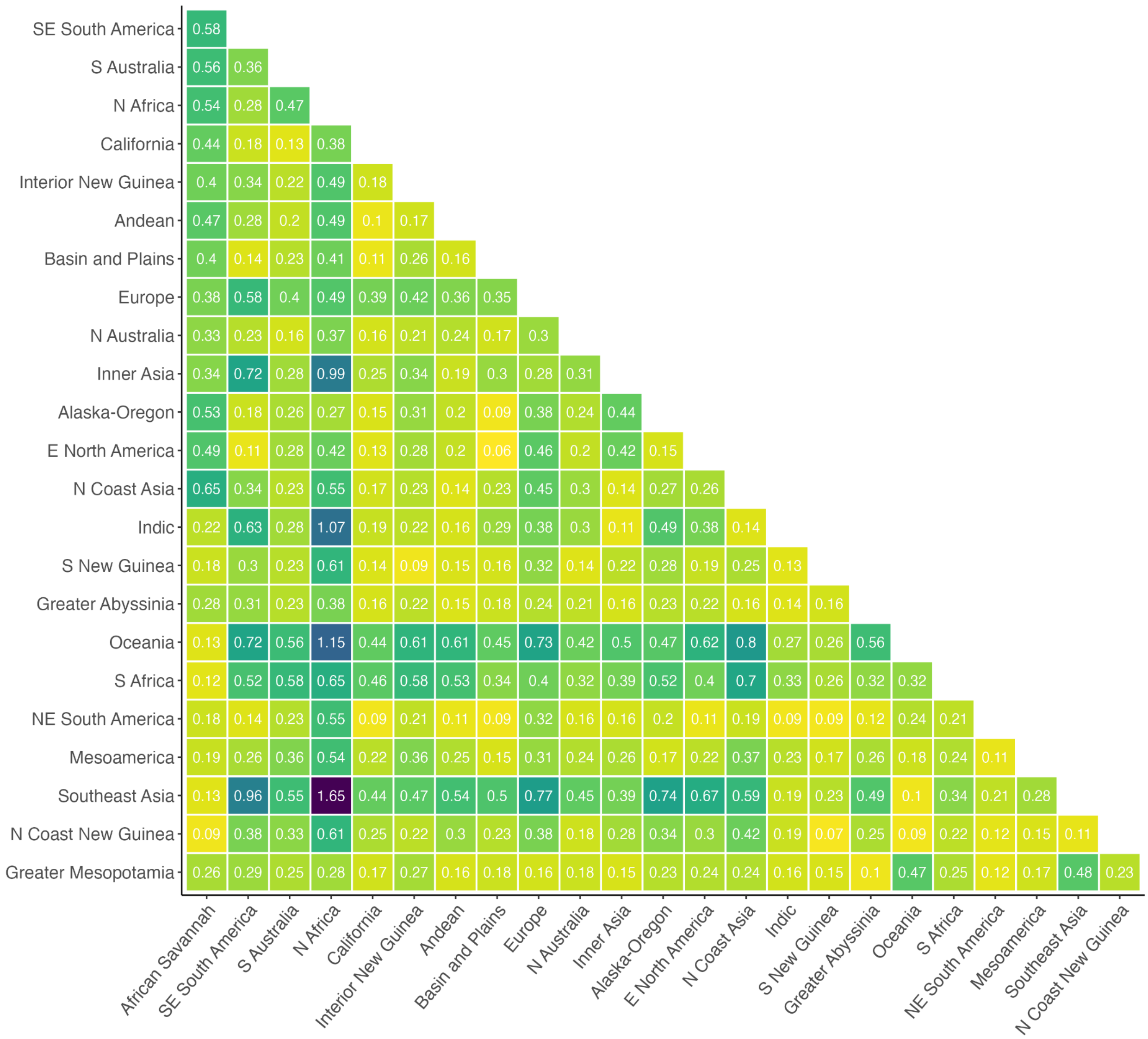

Figure S20. Pairwise Cultural Fixation scores over AUTOTYP-areas in the Grambank dataset.

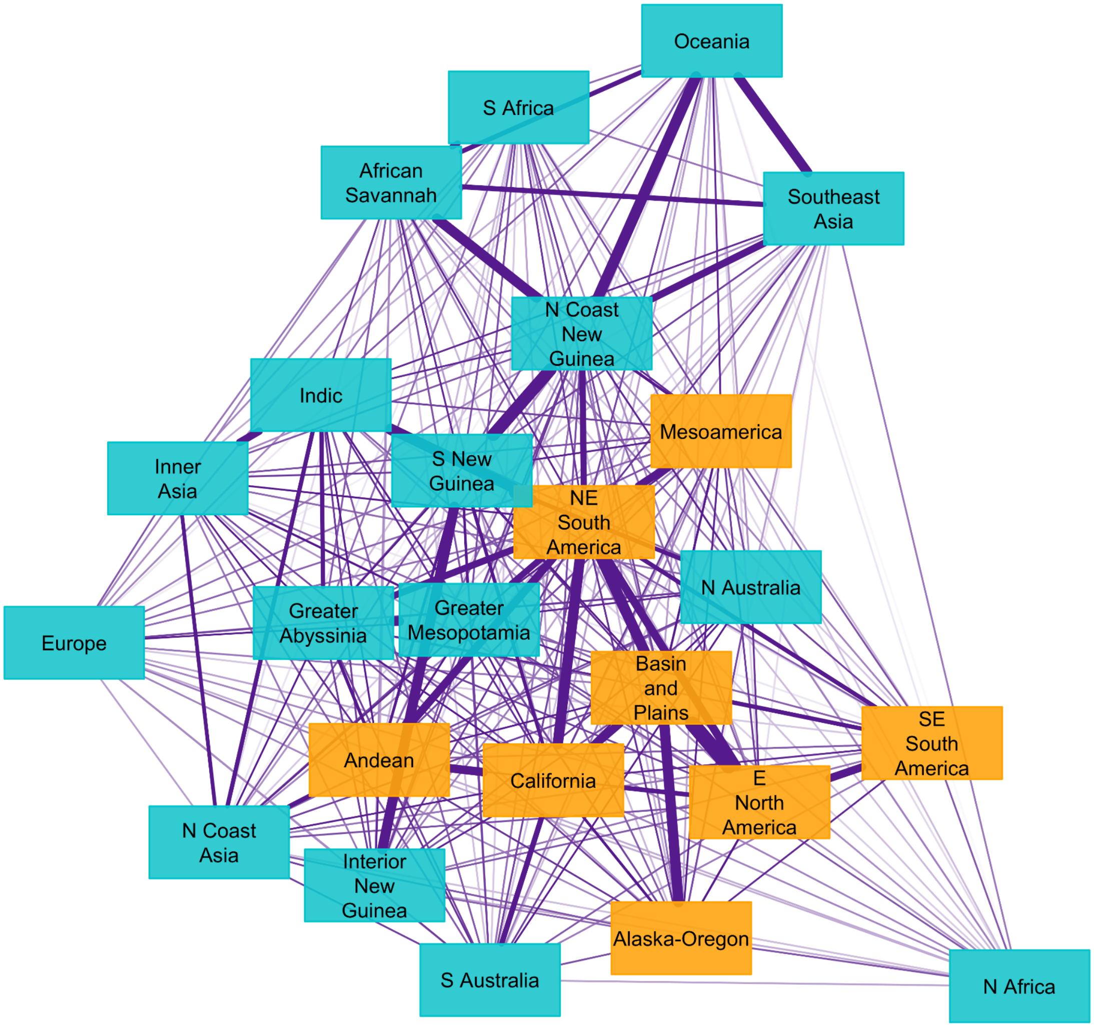

Figure S21. Network visualization of grammatical affinity between linguistic regions of the world. Languages are grouped by AUTOTYP areas, with areas in the Americas (orange) and areas elsewhere in the world (turquoise) represented in boxes. The thickness of lines between nodes indicates the strength of the affinity between areas, i.e. a thicker line indicates a lower Cultural Fixation score.

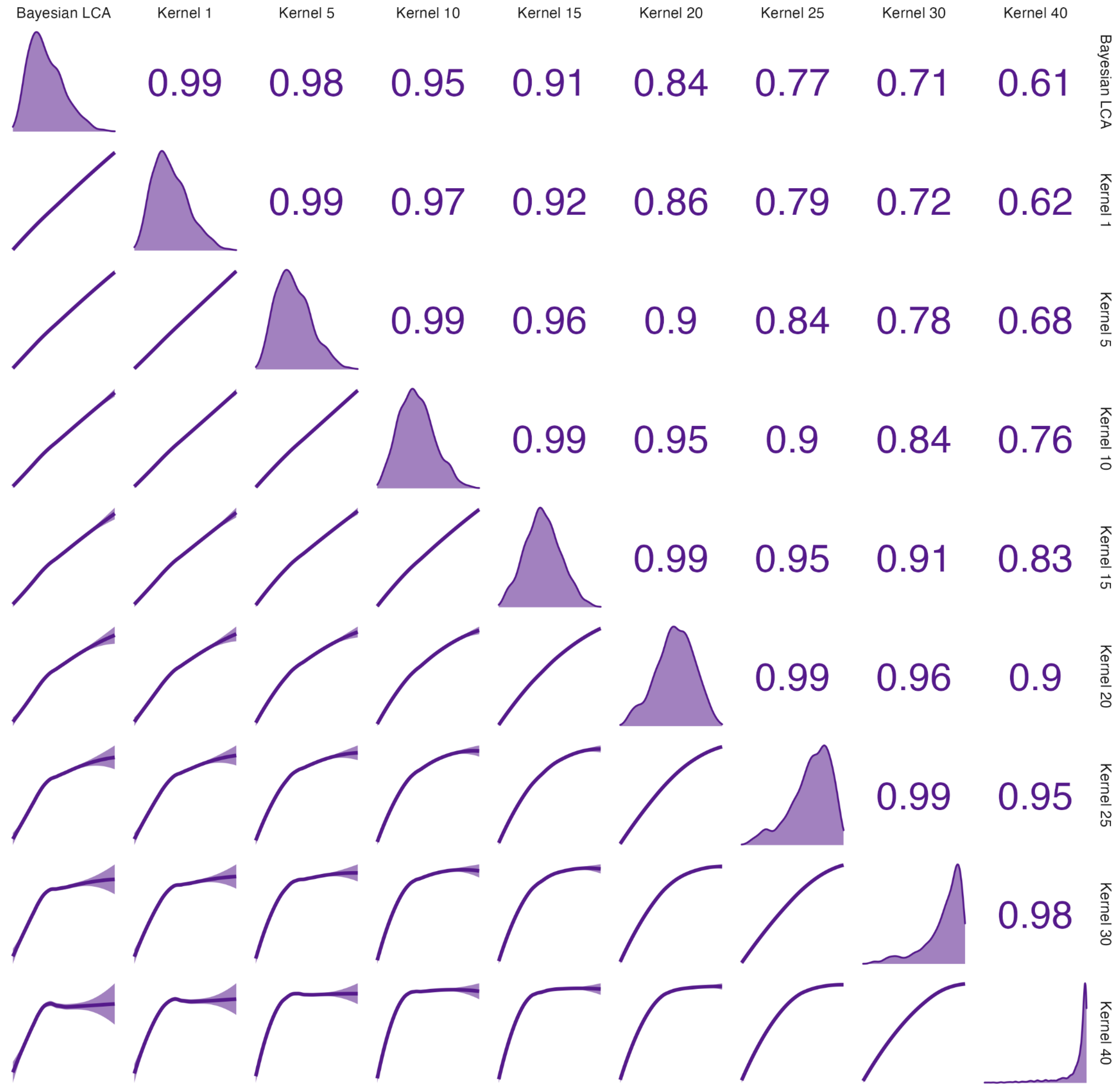

Figure S22. Comparison between different unusualness probability density estimation approaches. Each column/row corresponds to individual estimators. Lower triangle panels show smooth loess curves. Panels on the diagonal show probability densities. Upper triangle panels show Spearman correlation values.

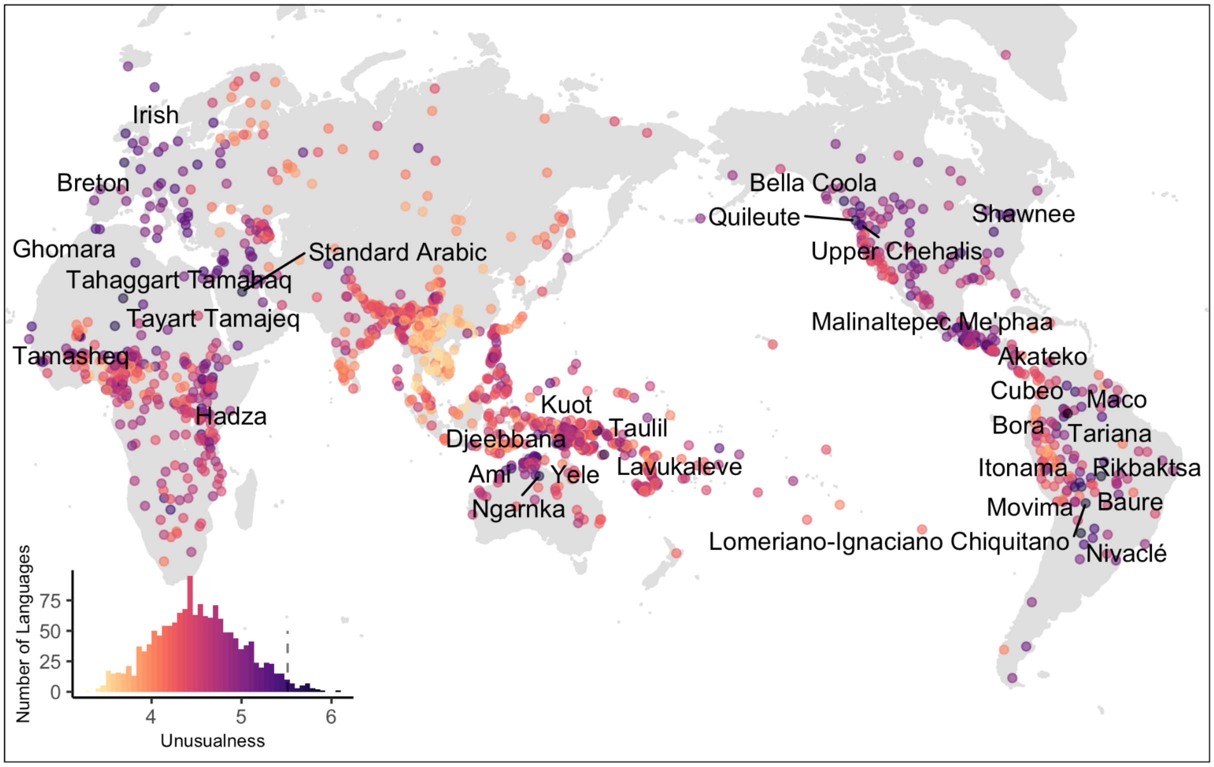

Figure S23. Map displaying the languages with the most unusual feature values. Languages are colored by how unusual their feature values are, and extreme languages are labeled. The inset histogram shows the overall distribution of unusualness scores across all the languages in Grambank, with the dashed line representing the cut-off limit to the top 2% used to identify the most unusual languages (labeled). This analysis uses Kernel 15.

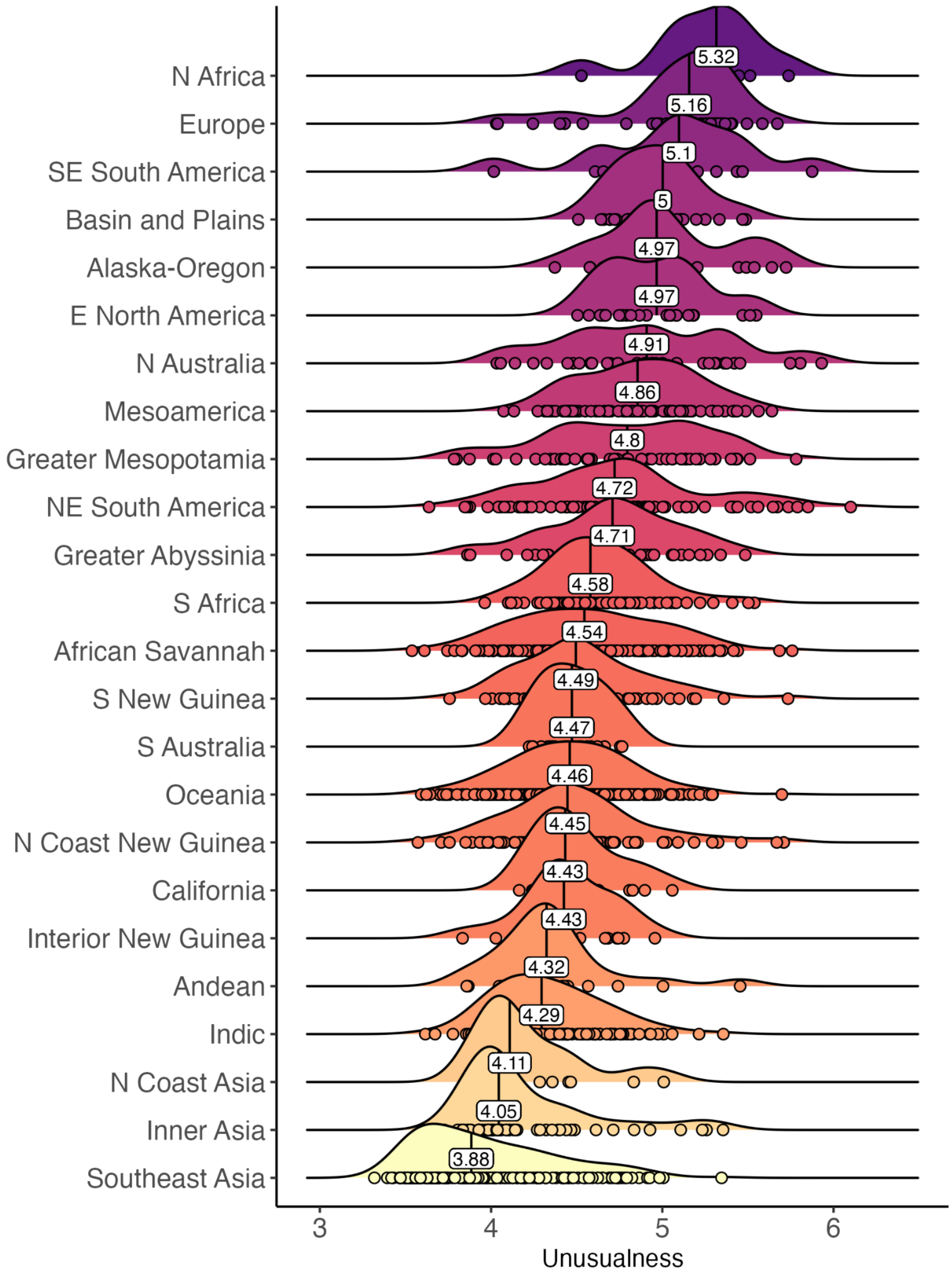

Figure S24. Distributions of unusualness scores (Kernel 15) per language as grouped by AUTOTYP area. The points represent each language and a value far to the right is more unusual. The line in each distribution and the label represents the median value per group.

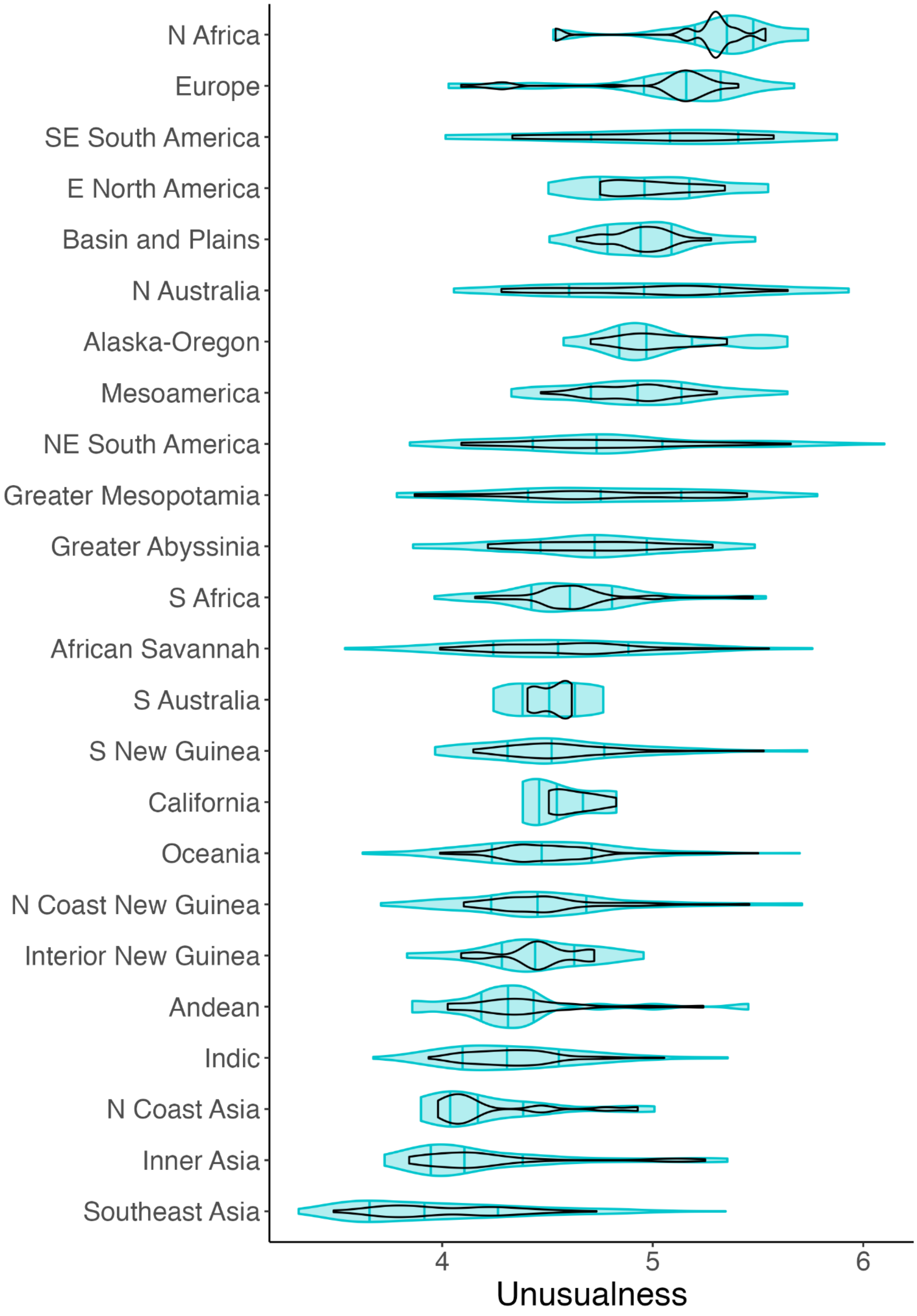

N Africa
Europe
SE South America
E North America
Basin and Plains
N Australia
Alaska-Oregon
Mesoamerica
NE South America
Greater Mesopotamia
Greater Abyssinia
S Africa
African Savannah
S Australia
S New Guinea
California
Oceania
N Coast New Guinea
Interior New Guinea
Andean
Indic
N Coast Asia
Inner Asia
Southeast Asia
4
5
6
Unusualness

Figure S25. Performance of the model for unusualness, displayed over cultural-historical areas. Light blue violin plots correspond to the unusualness score that the model predicts (samples from the posterior predictive distribution of the model), whereas the black-countour violin plots represent the known unusualness scores - the response variable.

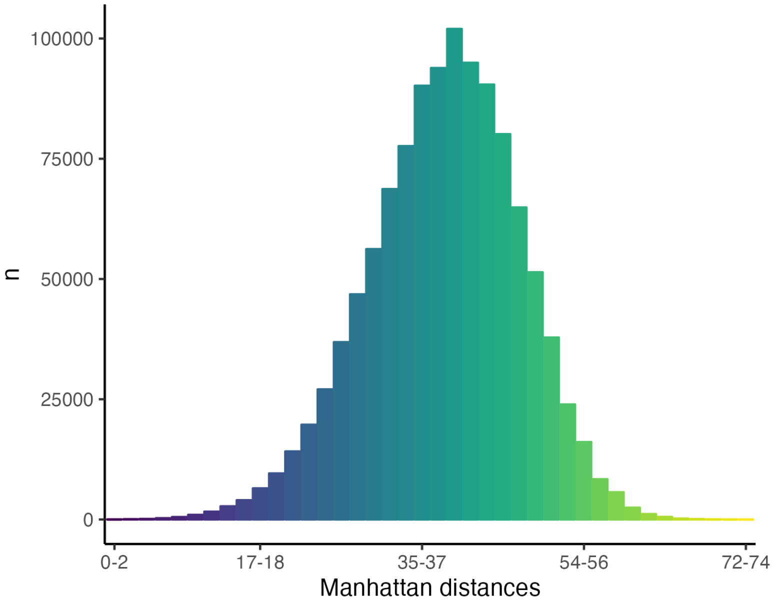

Figure S26. Histogram of Manhattan distances between languages in Grambank. Pairwise Manhattan distances show for each pair of languages in the dataset how many times they had different values, in absolute terms. The height of the bars show how many pairs of languages had that particular Manhattan distance. A Manhattan distance of 0 means that there were no features for which that language pair had different values. The mean Manhattan distance in the entire dataset is 39.

SM3 Supplementary tables

Table S1. Model fit scores (WAIC) of BRMS models with a beta-distribution prediction of the mean spatial and phylogenetic effects of Grambank features.

**Model**	**WAIC**	**SE (WAIC)**
null model (spatial)	-1424.07	85.86
domain model (spatial)	-1424.52	83.23
null model (phylogenetic)	-31.07	8.43
domain model (phylogenetic)	-25.64	8.48

Table S2. Phylogenetic and spatial effect in INLA model per feature.

Feature_ID	Phylogenetic effect (mean)	Phylogenetic effect (Standard Deviation)	Spatial effect (mean)	Spatial effect (Standard Deviation)
GB133	0.982	0.004	0	0
GB074	0.977	0.006	0	0
GB090	0.976	0.005	0	0
GB092	0.972	0.004	0	0
GB065a	0.962	0.008	0	0
GB057	0.955	0.018	0	0
GB031	0.948	0.01	0	0
GB043	0.941	0.024	0	0
GB094	0.941	0.017	0	0
GB075	0.939	0.01	0	0
GB171	0.937	0.02	0	0
GB431	0.936	0.019	0	0
GB089	0.933	0.016	0	0
GB091	0.933	0.015	0	0
GB081	0.93	0.022	0	0

GB058	0.926	0.026	0	0
GB196	0.926	0.039	0	0
GB198	0.925	0.027	0	0.001
GB170	0.921	0.025	0	0
GB025b	0.919	0.024	0	0
GB083	0.917	0.019	0	0
GB079	0.916	0.018	0	0
GB070	0.915	0.02	0	0
GB109	0.915	0.023	0	0
GB104	0.913	0.027	0	0
GB433	0.906	0.022	0	0
GB036	0.904	0.05	0	0
GB093	0.902	0.026	0.015	0.01
GB103	0.902	0.021	0	0
GB131	0.902	0.037	0.051	0.023
GB030	0.9	0.023	0	0
GB072	0.9	0.019	0	0

GB193b	0.893	0.052	0.032	0.027
GB051	0.891	0.039	0	0
GB059	0.89	0.024	0	0
GB022	0.881	0.05	0.028	0.016
GB172	0.881	0.054	0	0
GB108	0.879	0.033	0.001	0.002
GB028	0.878	0.035	0	0
GB114	0.869	0.039	0	0
GB086	0.861	0.029	0	0
GB053	0.855	0.038	0	0
GB193a	0.852	0.048	0.043	0.02
GB042	0.85	0.049	0	0
GB116	0.846	0.06	0	0
GB024b	0.843	0.045	0.095	0.032
GB044	0.843	0.037	0	0
GB155	0.842	0.058	0.071	0.032
GB318	0.838	0.052	0	0

GB130a	0.826	0.067	0.102	0.048
GB111	0.809	0.045	0	0
GB132	0.809	0.053	0.111	0.039
GB082	0.805	0.043	0	0
GB115	0.805	0.042	0.043	0.015
GB107	0.803	0.048	0.04	0.025
GB185	0.803	0.045	0	0
GB110	0.801	0.06	0	0
GB186	0.801	0.075	0	0
GB312	0.796	0.065	0.067	0.03
GB020	0.794	0.052	0.031	0.037
GB113	0.793	0.056	0.052	0.02
GB071	0.786	0.046	0.039	0.016
GB149	0.785	0.075	0	0
GB065b	0.784	0.044	0.093	0.029
GB192	0.777	0.087	0	0
GB054	0.774	0.09	0	0

GB147	0.758	0.058	0.121	0.039
GB096	0.753	0.087	0	0
GB068	0.745	0.056	0	0
GB117	0.743	0.066	0	0
GB309	0.735	0.062	0	0
GB024a	0.729	0.087	0.192	0.07
GB035	0.727	0.07	0	0
GB105	0.726	0.064	0	0
GB299	0.714	0.082	0.054	0.025
GB177	0.701	0.096	0	0
GB317	0.7	0.177	0	0
GB432	0.7	0.071	0.095	0.036
GB120	0.697	0.066	0	0
GB130b	0.696	0.099	0.154	0.061
GB025a	0.69	0.084	0.15	0.057
GB184	0.689	0.069	0	0
GB099	0.682	0.122	0	0

GB021	0.676	0.082	0	0
GB073	0.674	0.075	0.071	0.026
GB039	0.657	0.079	0	0
GB138	0.655	0.098	0.08	0.04
GB321	0.65	0.11	0	0
GB052	0.641	0.134	0	0
GB084	0.62	0.077	0.081	0.027
GB137	0.555	0.093	0.194	0.053
GB298	0.542	0.093	0	0
GB023	0.522	0.145	0.144	0.07
GB158	0.511	0.082	0	0
GB095	0.508	0.115	0	0
GB121	0.475	0.095	0	0
GB119	0.473	0.1	0	0
GB098	0.468	0.16	0	0
GB430	0.458	0.155	0	0
GB313	0.412	0.118	0	0

GB038	0.401	0.177	0.265	0.111
GB069	0.396	0.13	0	0
GB080	0.359	0.108	0.252	0.075
GB139	0.292	0.104	0.13	0.046
GB316	0.282	0.153	0	0
GB037	0.068	0.051	0	0
GB136	0.035	0.046	0.205	0.044
GB129	0	0	0	0
GB165	0	0	0	0
GB166	0	0	0	0
GB197	0	0	0	0
GB319	0	0	0	0
GB320	0	0	0	0

Table S3. Correlation coefficients of association between Principal Components and Theoretical scores, as calculated by PGLS.

**PC**	**Theoretical score**	**coef**	**t-value**	**p-value (of t)**
PC1	Word order	-0.09014	-4.77918	0

PC1	Flexivity	0.14755	9.42063	0
PC1	Noun class/gender	0.16118	8.06301	0
PC1	Locus of marking	-0.02264	-1.78043	0.07522
PC1	Fusion	0.45011	35.77013	0
PC1	Informativity	0.0778	7.02691	0
PC2	Word order	0.09187	-4.77918	0.00002
PC2	Flexivity	0.34598	9.42063	0
PC2	Noun class/gender	0.47968	8.06301	0
PC2	Locus of marking	0.08509	-1.78043	0
PC2	Fusion	0.35256	35.77013	0
PC2	Informativity	0.18245	7.02691	0
PC3	Word order	-0.03864	-4.77918	0.12904
PC3	Flexivity	-0.05418	9.42063	0.01215
PC3	Noun class/gender	0.16187	8.06301	0
PC3	Locus of marking	-0.15352	-1.78043	0
PC3	Fusion	-0.20571	35.77013	0
PC3	Informativity	-0.14179	7.02691	0

Table S4. Table of Grambank features.

ID	Name	Patrons
GB020	Are there definite or specific articles?	JLA JC
GB021	Do indefinite nominals commonly have indefinite articles?	JLA JC
GB022	Are there prenominal articles?	JLA JC
GB023	Are there postnominal articles?	JLA JC
GB024	What is the order of numeral and noun in the NP?	HJH
GB025	What is the order of adnominal demonstrative and noun?	JLA JC
GB026	Can adnominal property words occur discontinuously?	HJH
GB027	Are nominal conjunction and comitative expressed by different elements?	HS
GB028	Is there a distinction between inclusive and exclusive?	HJH
GB030	Is there a gender distinction in independent 3rd person pronouns?	HJH
GB031	Is there a dual or unit augmented form (in addition to plural or augmented) for all person categories in the pronoun system?	HJH
GB035	Are there three or more distance contrasts in demonstratives?	JLA JC
GB036	Do demonstratives show an elevation distinction?	JLA JC
GB037	Do demonstratives show a visible-nonvisible distinction?	JLA JC
GB038	Are there demonstrative classifiers?	JLA JC
GB039	Is there nonphonological allomorphy of noun number markers?	JLA JC
GB041	Are there several nouns (more than three) which are suppletive for number?	HS
GB042	Is there productive overt morphological singular marking on nouns?	HS
GB043	Is there productive morphological dual marking on nouns?	HS
GB044	Is there productive morphological plural marking on nouns?	HS
GB046	Is there an associative plural marker for nouns?	HS
GB047	Is there a productive morphological pattern for deriving an action/state noun from a verb?	HS
GB048	Is there a productive morphological pattern for deriving an agent noun from a verb?	HS
GB049	Is there a productive morphological pattern for deriving an object noun from a verb?	HS
GB051	Is there a gender/noun class system where sex is a factor in class assignment?	HJH
GB052	Is there a gender/noun class system where shape is a factor in class assignment?	HJH
GB053	Is there a gender/noun class system where animacy is a factor in class assignment?	HJH

GB054	Is there a gender/noun class system where plant status is a factor in class assignment?	HJH
GB057	Are there numeral classifiers?	JLA JC
GB058	Are there possessive classifiers?	JLA JC
GB059	Is the adnominal possessive construction different for alienable and inalienable nouns?	HJH
GB065	What is the pragmatically unmarked order of adnominal possessor noun and possessed noun?	HJH
GB068	Do core adjectives (defined semantically as property concepts such as value, shape, age, dimension) act like verbs in predicative position?	JLA JC
GB069	Do core adjectives (defined semantically as property concepts; value, shape, age, dimension) used attributively require the same morphological treatment as verbs?	JLA JC
GB070	Are there morphological cases for non-pronominal core arguments (i.e. S/A/P)?	JLE
GB071	Are there morphological cases for pronominal core arguments (i.e. S/A/P)?	JLE
GB072	Are there morphological cases for oblique non-pronominal NPs (i.e. not S/A/P)?	JLE
GB073	Are there morphological cases for independent oblique personal pronominal arguments (i.e. not S/A/P)?	JLE
GB074	Are there prepositions?	JLE
GB075	Are there postpositions?	JLE
GB079	Do verbs have prefixes/proclitics, other than those that only mark A, S or P (do include portmanteau: A & S + TAM)?	JLE
GB080	Do verbs have suffixes/enclitics, other than those that only mark A, S or P (do include portmanteau: A & S + TAM)?	JLE
GB081	Is there productive infixation in verbs?	HJH
GB082	Is there overt morphological marking of present tense on verbs?	HS
GB083	Is there overt morphological marking on the verb dedicated to past tense?	HS
GB084	Is there overt morphological marking on the verb dedicated to future tense?	HS
GB086	Is a morphological distinction between perfective and imperfective aspect available on verbs?	HS
GB089	Can the S argument be indexed by a suffix/enclitic on the verb in the simple main clause?	AWM
GB090	Can the S argument be indexed by a prefix/proclitic on the verb in the simple main clause?	AWM
GB091	Can the A argument be indexed by a suffix/enclitic on the verb in the simple main clause?	AWM
GB092	Can the A argument be indexed by a prefix/proclitic on the verb in the simple main clause?	AWM
GB093	Can the P argument be indexed by a suffix/enclitic on the verb in the simple main clause?	AWM
GB094	Can the P argument be indexed by a prefix/proclitic on the verb in the simple main clause?	AWM

GB095	Are variations in marking strategies of core participants based on TAM distinctions?	AWM
GB096	Are variations in marking strategies of core participants based on verb classes?	AWM
GB098	Are variations in marking strategies of core participants based on person distinctions?	AWM
GB099	Can verb stems alter according to the person of a core participant?	AWM
GB103	Is there a benefactive applicative marker on the verb (including indexing)?	JLE
GB104	Is there an instrumental applicative marker on the verb (including indexing)?	JLE
GB105	Can the recipient in a ditransitive construction be marked like the monotransitive patient?	AWM
GB107	Can standard negation be marked by an affix, clitic or modification of the verb?	HS
GB108	Is there directional or locative morphological marking on verbs?	JLE
GB109	Is there verb suppletion for participant number?	HS
GB110	Is there verb suppletion for tense or aspect?	HS
GB111	Are there conjugation classes?	JLA JC
GB113	Are there verbal affixes or clitics that turn intransitive verbs into transitive ones?	JLE
GB114	Is there a phonologically bound reflexive marker on the verb?	JLE
GB115	Is there a phonologically bound reciprocal marker on the verb?	JLE
GB116	Do verbs classify the shape, size or consistency of absolutive arguments by means of incorporated nouns, verbal affixes or suppletive verb stems?	JLA JC
GB117	Is there a copula for predicate nominals?	JLA JC
GB118	Are there serial verb constructions?	JLA JC
GB119	Can mood be marked by an inflecting word ('auxiliary verb')?	HS
GB120	Can aspect be marked by an inflecting word ('auxiliary verb')?	HS
GB121	Can tense be marked by an inflecting word ('auxiliary verb')?	HS
GB122	Is verb compounding a regular process?	JLA JC
GB123	Are there verb-adjunct (aka light-verb) constructions?	JLA JC
GB124	Is incorporation of nouns into verbs a productive intransitivizing process?	HJH
GB126	Is there an existential verb?	HS
GB127	Are different posture verbs used obligatorily depending on an inanimate locatum's shape or position (e.g. 'to lie' vs. 'to stand')?	JLE
GB129	Is there a notably small number, i.e. about 100 or less, of verb roots in the language?	HS
GB130	What is the pragmatically unmarked order of S and V in intransitive clauses?	HJH

GB131	Is a pragmatically unmarked constituent order verb-initial for transitive clauses?	HJH
GB132	Is a pragmatically unmarked constituent order verb-medial for transitive clauses?	HJH
GB133	Is a pragmatically unmarked constituent order verb-final for transitive clauses?	HJH
GB134	Is the order of constituents the same in main and subordinate clauses?	HJH
GB135	Do clausal objects usually occur in the same position as nominal objects?	HJH
GB136	Is the order of core argument (i.e. S/A/P) constituents fixed?	HJH
GB137	Can standard negation be marked clause-finally?	HJH
GB138	Can standard negation be marked clause-initially?	HJH
GB139	Is there a difference between imperative (prohibitive) and declarative negation constructions?	HS
GB140	Is verbal predication marked by the same negator as all of the following types of predication: locational, existential and nominal?	HS
GB146	Is there a morpho-syntactic distinction between predicates expressing controlled versus uncontrolled events or states?	JLE
GB147	Is there a morphological passive marked on the lexical verb?	JLE
GB148	Is there a morphological antipassive marked on the lexical verb?	JLE
GB149	Is there a morphologically marked inverse on verbs?	JLE
GB150	Is there clause chaining?	HJH
GB151	Is there an overt verb marker dedicated to signalling coreference or noncoreference between the subject of one clause and an argument of an adjacent clause ('switch reference')?	HJH
GB152	Is there a morphologically marked distinction between simultaneous and sequential clauses?	HJH
GB155	Are causatives formed by affixes or clitics on verbs?	JLE
GB156	Is there a causative construction involving an element that is unmistakably grammaticalized from a verb for 'to say'?	JLE
GB158	Are verbs reduplicated?	JLE
GB159	Are nouns reduplicated?	JLE
GB160	Are elements apart from verbs or nouns reduplicated?	JLE
GB165	Is there productive morphological trial marking on nouns?	HS
GB166	Is there productive morphological paucal marking on nouns?	HS
GB167	Is there a logophoric pronoun?	HJH
GB170	Can an adnominal property word agree with the noun in gender/noun class?	JLA JC
GB171	Can an adnominal demonstrative agree with the noun in gender/noun class?	JLA JC

GB172	Can an article agree with the noun in gender/noun class?	JLA JC
GB177	Can the verb carry a marker of animacy of argument, unrelated to any gender/noun class of the argument visible in the NP domain?	AWM
GB184	Can an adnominal property word agree with the noun in number?	JLA JC
GB185	Can an adnominal demonstrative agree with the noun in number?	JLA JC
GB186	Can an article agree with the noun in number?	JLA JC
GB187	Is there any productive diminutive marking on the noun (exclude marking by system of nominal classification only)?	JLA JC
GB188	Is there any productive augmentative marking on the noun (exclude marking by system of nominal classification only)?	JLA JC
GB192	Is there a gender system where a noun's phonological properties are a factor in class assignment?	HJH
GB193	What is the order of adnominal property word and noun?	JLA JC
GB196	Is there a male/female distinction in 2nd person independent pronouns?	HJH
GB197	Is there a male/female distinction in 1st person independent pronouns?	HJH
GB198	Can an adnominal numeral agree with the noun in gender/noun class?	JLA JC
GB203	What is the order of the adnominal collective universal quantifier ('all') and the noun?	HJH
GB204	Do collective ('all') and distributive ('every') universal quantifiers differ in their forms or their syntactic positions?	HJH
GB250	Can predicative possession be expressed with a transitive 'habeo' verb?	HS
GB252	Can predicative possession be expressed with an S-like possessum and a locative-coded possessor?	HS
GB253	Can predicative possession be expressed with an S-like possessum and a dative-coded possessor?	HS
GB254	Can predicative possession be expressed with an S-like possessum and a possessor that is coded like an adnominal possessor?	HS
GB256	Can predicative possession be expressed with an S-like possessor and a possessum that is coded like a comitative argument?	HS
GB257	Can polar interrogation be marked by intonation only?	JLA JC
GB260	Can polar interrogation be indicated by a special word order?	JLA JC
GB262	Is there a clause-initial polar interrogative particle?	JLA JC
GB263	Is there a clause-final polar interrogative particle?	JLA JC
GB264	Is there a polar interrogative particle that most commonly occurs neither clause-initially nor clause-finally?	JLA JC

GB265	Is there a comparative construction that includes a form that elsewhere means 'surpass, exceed'?	HJH
GB266	Is there a comparative construction that employs a marker of the standard which elsewhere has a locational meaning?	HJH
GB270	Can comparatives be expressed using two conjoined clauses?	HJH
GB273	Is there a comparative construction with a standard marker that elsewhere has neither a locational meaning nor a 'surpass/exceed' meaning?	HJH
GB275	Is there a bound comparative degree marker on the property word in a comparative construction?	HJH
GB276	Is there a non-bound comparative degree marker modifying the property word in a comparative construction?	HJH
GB285	Can polar interrogation be marked by a question particle and verbal morphology?	JLA JC
GB286	Can polar interrogation be indicated by overt verbal morphology only?	JLA JC
GB291	Can polar interrogation be marked by tone?	JLA JC
GB296	Is there a phonologically or morphosyntactically definable class of ideophones that includes ideophones depicting imagery beyond sound?	JLE
GB297	Can polar interrogation be indicated by a V-not-V construction?	JLA JC
GB298	Can standard negation be marked by an inflecting word ('auxiliary verb')?	HS
GB299	Can standard negation be marked by a non-inflecting word ('auxiliary particle')?	HS
GB300	Does the verb for 'give' have suppletive verb forms?	HS
GB301	Is there an inclusory construction?	JLA JC
GB302	Is there a phonologically free passive marker ('particle' or 'auxiliary')?	JLE
GB303	Is there a phonologically free antipassive marker ('particle' or 'auxiliary')?	JLE
GB304	Can the agent be expressed overtly in a passive clause?	JLE
GB305	Is there a phonologically independent reflexive pronoun?	JLE
GB306	Is there a phonologically independent non-bipartite reciprocal pronoun?	JLE
GB309	Are there multiple past or multiple future tenses, distinguishing distance from Time of Reference?	HS
GB312	Is there overt morphological marking on the verb dedicated to mood?	HS
GB313	Are there special adnominal possessive pronouns that are not formed by an otherwise regular process?	HJH
GB314	Can augmentative meaning be expressed productively by a shift of gender/noun class?	JLA JC
GB315	Can diminutive meaning be expressed productively by a shift of gender/noun class?	JLA JC

GB316	Is singular number regularly marked in the noun phrase by a dedicated phonologically free element?	HS
GB317	Is dual number regularly marked in the noun phrase by a dedicated phonologically free element?	HS
GB318	Is plural number regularly marked in the noun phrase by a dedicated phonologically free element?	HS
GB319	Is trial number regularly marked in the noun phrase by a dedicated phonologically free element?	HS
GB320	Is paucal number regularly marked in the noun phrase by a dedicated phonologically free element?	HS
GB321	Is there a large class of nouns whose gender/noun class is not phonologically or semantically predictable?	HJH
GB322	Is there grammatical marking of direct evidence (perceived with the senses)?	HJH
GB323	Is there grammatical marking of indirect evidence (hearsay, inference, etc.)?	HJH
GB324	Is there an interrogative verb for content interrogatives (who?, what?, etc.)?	HJH
GB325	Is there a count/mass distinction in interrogative quantifiers?	HJH
GB326	Do (nominal) content interrogatives normally or frequently occur in situ?	HJH
GB327	Can the relative clause follow the noun?	JLE
GB328	Can the relative clause precede the noun?	JLE
GB329	Are there internally-headed relative clauses?	JLE
GB330	Are there correlative relative clauses?	JLE
GB331	Are there non-adjacent relative clauses?	JLE
GB333	Is there a decimal numeral system?	JLE
GB334	Is there synchronic evidence for any element of a quinary numeral system?	JLE
GB335	Is there synchronic evidence for any element of a vigesimal numeral system?	JLE
GB336	Is there a body-part tallying system?	JLE
GB400	Are all person categories neutralized in some voice, tense, aspect, mood and/or negation?	AWM
GB401	Is there a class of patient-labile verbs?	AWM
GB402	Does the verb for 'see' have suppletive verb forms?	HS
GB403	Does the verb for 'come' have suppletive verb forms?	HS
GB408	Is there any accusative alignment of flagging?	AWM
GB409	Is there any ergative alignment of flagging?	AWM

GB410	Is there any neutral alignment of flagging?	AWM
GB415	Is there a politeness distinction in 2nd person forms?	HJH
GB421	Is there a preposed complementizer in complements of verbs of thinking and/or knowing?	HS
GB422	Is there a postposed complementizer in complements of verbs of thinking and/or knowing?	HS
GB430	Can adnominal possession be marked by a prefix on the possessor?	HJH
GB431	Can adnominal possession be marked by a prefix on the possessed noun?	HJH
GB432	Can adnominal possession be marked by a suffix on the possessor?	HJH
GB433	Can adnominal possession be marked by a suffix on the possessed noun?	HJH
GB519	Can mood be marked by a non-inflecting word ('auxiliary particle')?	HS
GB520	Can aspect be marked by a non-inflecting word ('auxiliary particle')?	HS
GB521	Can tense be marked by a non-inflecting word ('auxiliary particle')?	HS
GB522	Can the S or A argument be omitted from a pragmatically unmarked clause when the referent is inferrable from context ('pro-drop' or 'null anaphora')?	HJH

Table S5. Table of binarised Grambank features.

ID	Abbreviation
GB024a	GB024a NUMOrder_Num-N
GB024b	GB024b NUMOrder_N-Num
GB025a	GB025a DEMOrder_Dem-N
GB025b	GB025b DEMOrder_N-Dem
GB065a	GB065a POSSOrder_PSR-PSD
GB065b	GB065b POSSOrder_PSD-PSR
GB130a	GB130a IntransOrder_SV
GB130b	GB130b IntransOrder_VS
GB193a	GB193a ANMOrder_ANM-N
GB193b	GB193b ANMOrder_N-ANM
GB203a	GB203a UQOrder_UQ-N
GB203b	GB203b UQOrder_N-UQ

Table S6. Grambank features with information on theoretical scores and predictions from Nichols (*20*).

Feature_ID	Fusion	Flexivity	Gender/noun class	locus of marking	word order	informativity	Main_domain	Nichols_1995_label	Nichols_1995_prediction
GB303						antipassive	clause		
GB149	1					inverse	verbal domain		
GB070	1			0			nominal domain		
GB071	0.5			0			pronoun		
GB408				0			nominal domain	Dom alignment	G
GB409				0			nominal domain	Dom alignment	G
GB410				0			nominal domain	Dom alignment	G
GB074					1		nominal domain	Adposition place	G
GB075					0		nominal domain	Adposition place	G
GB080	1						verbal domain		
GB081	1						verbal domain		
GB079	1						verbal domain		
GB092	1			1			verbal domain	1 agreement	G
GB093	1			1			verbal domain	2 agreement	

GB089	1			1			verbal domain	1 agreement	G
GB090	1			1			verbal domain	1 agreement	G
GB091	1			1			verbal domain	1 agreement	G
GB094	1			1			verbal domain	2 agreement	
GB098		1					verbal domain		
GB095		1					verbal domain		
GB096		1					verbal domain		
GB105							clause		
GB072	1			0			nominal domain		
GB073	0.5			0			pronoun		
GB108	1					directional	verbal domain		
GB027						comitative	clause		
GB103	1					benefactive	verbal domain		
GB104	1					instrumental	verbal domain		
GB026							nominal domain		
GB193							nominal domain		

GB069							nominal domain		
GB065							nominal domain		A
GB059						alienabilit y	nominal domain		
GB430	1			0			nominal domain		
GB431	1			1			nominal domain	Noun Poss. Place	A
GB432	1			0			nominal domain		
GB433	1			1			nominal domain	Noun Poss. Place	A
GB313							pronoun		
GB058		1	0			possessive classifiers	nominal domain		
GB155	1						verbal domain	+A	G
GB156							clause		
GB028						clusivity	pronoun	Incl/excl	G
GB301							clause		
GB265							clause		
GB270							clause		
GB273							clause		

GB275	1						clause		
GB276							clause		
GB266							clause		
GB146	0.5					control	nominal domain		
GB020						definitearticles	nominal domain		
GB022					0		nominal domain		
GB021						indef	nominal domain		
GB023					1		nominal domain		
GB035						demonstartivedistance	nominal domain		
GB037						demonstrativevisibility	nominal domain		
GB036						demonstrativeelevation	nominal domain		
GB151	1					switch reference	verbal domain		
GB025							nominal domain		
GB038		1	0			demonstrative classifiers	nominal domain		
GB159							nominal domain		

GB160							nominal domain		
GB158							verbal domain		
GB048	0.5						nominal domain		
GB049	0.5						nominal domain		
GB047	0.5						nominal domain		
GB321		1	1				nominal domain		
GB051		1	1			gendersex	nominal domain	Genders	G
GB052		1	1			gendershape	nominal domain	Genders	G
GB054		1	1			genderplant	nominal domain	Genders	G
GB192		1	1				nominal domain	Genders	G
GB196			1			pronoungender2	pronoun		
GB197			1			pronoungender1	pronoun		
GB053		1	1			genderanimacy	nominal domain	Genders	G
GB170	1	1	1	0			nominal domain	Genders	G
GB171	1	1	1	0			nominal domain	Genders	G

GB172	1	1	1	0			nominal domain		
GB314						augmentative	nominal domain		
GB315						diminutive	nominal domain		
GB296							nominal domain		
GB167						pronounlog	pronoun		
GB257							clause		
GB260							clause		
GB262					1		clause		
GB263							clause		
GB264							clause		
GB285	1						clause		
GB286	1						clause		
GB291							clause		
GB324							clause		
GB326							clause		
GB325						count_mass	nominal domain		

GB116		1				verbclassify	verbal domain		
GB177	1	1	1	1			verbal domain		
GB057		1	0			numera classifers	nominal domain	Numeral Classifier	G, A
GB188	1	1				augmentative	nominal domain		
GB187	1	1				diminutive	nominal domain		
GB046						assocplural	nominal domain		
GB316						singular	nominal domain		
GB317						dual	nominal domain		
GB318						plural	nominal domain		
GB319						trial	nominal domain		
GB320						paucal	nominal domain		
GB039		1					nominal domain		
GB165	1			1		trial	nominal domain		
GB166	1			1		paucal	nominal domain		
GB041		1					nominal domain		

GB043	1			1		dual	nominal domain		
GB109		1		1			verbal domain		
GB184	1			0			nominal domain		
GB185	1			0			nominal domain		
GB186	1			0			nominal domain		
GB044	1			1		plural	nominal domain	Noun Sg/Pl	G
GB042	1			1		singular	nominal domain		
GB302						passive	clause	-A	G
GB304							clause		
GB099		1		1			verbal domain		
GB031						pronoundualaug	pronoun		
GB030		1	1			pronoungender3	pronoun		
GB400							verbal domain		
GB415						politeness	pronoun		
GB132							clause	Word order	A
GB118							verbal domain		

GB131					1		clause	Word order	A
GB136							clause		
GB130							clause	Word order	A
GB522							clause		
GB133					0		clause	Word order	A
GB150							clause		
GB122							verbal domain		
GB123							verbal domain		
GB140						differentneg	clause		
GB256							clause		
GB253							clause		
GB254							clause		
GB252							clause		
GB135							clause		
GB134							clause		
GB068							nominal domain		
GB117						copulaprednom	verbal domain		

GB333							numeral		
GB334							numeral		
GB335							numeral		
GB336							numeral		
GB024							nominal domain		
GB203							nominal domain		
GB204							nominal domain		
GB198	1	1	1	0			nominal domain	Genders	G
GB115	1			1		reciprocity	verbal domain		
GB114	1			1		reflexivity	verbal domain		
GB327					1		nominal domain		
GB328					0		clause		
GB329							clause		
GB330							clause		
GB331							clause		
GB421					1		clause		

GB422					0		clause		
GB086	1					aspect	verbal domain		
GB120	1					aspect	verbal domain		
GB520						aspect	verbal domain		
GB322						evidentiality_direct	verbal domain		
GB323						evidentiality_indirect	verbal domain		
GB139						prohibitive	clause		
GB297							clause		
GB119	1					mood	verbal domain		
GB312	1					mood	verbal domain		
GB519						mood	verbal domain		
GB138							clause		
GB107	1						verbal domain		
GB137							clause		
GB298	1						clause		
GB299							clause		
GB152	1					simultanseq	clause		

GB084	1					tense	verbal domain		
GB309						multiplete nse	verbal domain		
GB521						tense	verbal domain		
GB082	1					tense	verbal domain		
GB083	1					tense	verbal domain		
GB121	1					tense	verbal domain		
GB110		1					verbal domain		
GB111		1					verbal domain		
GB148	1					antipassiv e	verbal domain		
GB113	1						verbal domain	+A	A
GB147	1					passive	verbal domain	-A	G
GB305						reflexivity	pronoun		
GB306						reciprocity	pronoun		
GB124							verbal domain	-A	G
GB401							verbal domain		
GB129							verbal domain		
GB127						posturever bs	verbal domain		

GB126						existential verb	verbal domain		
GB250							nominal domain		
GB402		1					verbal domain		
GB403		1					verbal domain		
GB300		1					verbal domain		
GB024a							nominal domain		
GB024b							nominal domain		
GB025a							nominal domain		
GB025b							nominal domain		
GB065a							nominal domain		
GB065b							nominal domain		
GB130a							clause		
GB130b							clause		
GB193a							nominal domain		
GB193b							nominal domain		
GB203a							nominal domain		

GB203b							nominal domain		

Table S7. Cultural Fixation Scores between AUTOTYP-areas.

Group_Var1	Group_Var2	Cultural Fixation Score	Americas_Var1	Americas_Var2
Basin and Plains	E North America	0.058	americas	americas
S New Guinea	N Coast New Guinea	0.0746	not americas	not americas
S New Guinea	NE South America	0.0851	not americas	americas
Oceania	N Coast New Guinea	0.0863	not americas	not americas
Basin and Plains	NE South America	0.0876	americas	americas
California	NE South America	0.0897	americas	americas
Indic	NE South America	0.0898	not americas	americas
Basin and Plains	Alaska-Oregon	0.0903	americas	americas
Interior New Guinea	S New Guinea	0.092	not americas	not americas
N Coast New Guinea	African Savannah	0.0923	not americas	not americas
Oceania	Southeast Asia	0.0961	not americas	not americas
Greater Abyssinia	Greater Mesopotamia	0.0979	not americas	not americas
California	Andean	0.1044	americas	americas

California	Basin and Plains	0.1068	americas	americas
E North America	NE South America	0.1082	americas	americas
Inner Asia	Indic	0.1086	not americas	not americas
Andean	NE South America	0.109	americas	americas
SE South America	E North America	0.1092	americas	americas
NE South America	Mesoamerica	0.111	americas	americas
Southeast Asia	N Coast New Guinea	0.1148	not americas	not americas
Greater Abyssinia	NE South America	0.1177	not americas	americas
NE South America	N Coast New Guinea	0.1221	americas	not americas
S Africa	African Savannah	0.1241	not americas	not americas
NE South America	Greater Mesopotamia	0.1246	americas	not americas
S Australia	California	0.1284	not americas	americas
Southeast Asia	African Savannah	0.1306	not americas	not americas
Indic	S New Guinea	0.1307	not americas	not americas
Oceania	African Savannah	0.1337	not americas	not americas
California	E North America	0.1343	americas	americas
N Coast Asia	Indic	0.1346	not americas	not americas

Andean	N Coast Asia	0.135	americas	not americas
N Australia	S New Guinea	0.1367	not americas	not americas
SE South America	NE South America	0.1373	americas	americas
SE South America	Basin and Plains	0.1376	americas	americas
Inner Asia	N Coast Asia	0.1378	not americas	not americas
Indic	Greater Abyssinia	0.1381	not americas	not americas
California	S New Guinea	0.1445	americas	not americas
Andean	Greater Abyssinia	0.1463	americas	not americas
Mesoamerica	N Coast New Guinea	0.1489	americas	not americas
Andean	S New Guinea	0.1494	americas	not americas
California	Alaska-Oregon	0.15	americas	americas
Basin and Plains	Mesoamerica	0.1539	americas	americas
S New Guinea	Greater Mesopotamia	0.1542	not americas	not americas
Alaska-Oregon	E North America	0.1547	americas	americas
Andean	Indic	0.1553	americas	not americas
Inner Asia	Greater Abyssinia	0.1562	not americas	not americas
Andean	Greater Mesopotamia	0.157	americas	not americas

California	Greater Abyssinia	0.157	americas	not americas
N Australia	NE South America	0.1572	not americas	americas
Inner Asia	NE South America	0.1573	not americas	americas
N Coast Asia	Greater Abyssinia	0.1573	not americas	not americas
Inner Asia	Greater Mesopotamia	0.1578	not americas	not americas
Europe	Greater Mesopotamia	0.1585	not americas	not americas
California	N Australia	0.1611	americas	not americas
Basin and Plains	S New Guinea	0.1625	americas	not americas
S New Guinea	Greater Abyssinia	0.1631	not americas	not americas
Andean	Basin and Plains	0.1636	americas	americas
S Australia	N Australia	0.1648	not americas	not americas
Alaska-Oregon	Mesoamerica	0.1657	americas	americas
California	N Coast Asia	0.1674	americas	not americas
S New Guinea	Mesoamerica	0.1693	not americas	americas
Basin and Plains	N Australia	0.1696	americas	not americas
Indic	Greater Mesopotamia	0.1704	not americas	not americas
California	Greater Mesopotamia	0.1711	americas	not americas

Interior New Guinea	Andean	0.1729	not americas	americas
N Australia	N Coast New Guinea	0.176	not americas	not americas
California	Interior New Guinea	0.1762	americas	not americas
Oceania	Mesoamerica	0.1779	not americas	americas
NE South America	African Savannah	0.1793	americas	not americas
Mesoamerica	Greater Mesopotamia	0.1803	americas	not americas
N Australia	Greater Mesopotamia	0.1803	not americas	not americas
Basin and Plains	Greater Mesopotamia	0.181	americas	not americas
S New Guinea	African Savannah	0.1819	not americas	not americas
Andean	Inner Asia	0.1821	americas	not americas
Basin and Plains	Greater Abyssinia	0.1824	americas	not americas
SE South America	Alaska-Oregon	0.1825	americas	americas
SE South America	California	0.1837	americas	americas
N Coast Asia	NE South America	0.1856	not americas	americas
Indic	N Coast New Guinea	0.1873	not americas	not americas
Indic	Southeast Asia	0.1892	not americas	not americas
Mesoamerica	African Savannah	0.1898	americas	not americas

California	Indic	0.1919	americas	not americas
E North America	S New Guinea	0.1925	americas	not americas
Alaska-Oregon	NE South America	0.1971	americas	americas
Andean	E North America	0.1999	americas	americas
N Australia	E North America	0.2005	not americas	americas
S Australia	Andean	0.2029	not americas	americas
Andean	Alaska-Oregon	0.2036	americas	americas
N Australia	Greater Abyssinia	0.2098	not americas	not americas
Interior New Guinea	NE South America	0.21	not americas	americas
S Africa	NE South America	0.2109	not americas	americas
NE South America	Southeast Asia	0.2112	americas	not americas
Interior New Guinea	N Australia	0.2123	not americas	not americas
Inner Asia	S New Guinea	0.215	not americas	not americas
S Africa	N Coast New Guinea	0.217	not americas	not americas
Indic	African Savannah	0.2171	not americas	not americas
Interior New Guinea	N Coast New Guinea	0.2181	not americas	not americas
E North America	Mesoamerica	0.2181	americas	americas

Interior New Guinea	Greater Abyssinia	0.2189	not americas	not americas
Interior New Guinea	Indic	0.2192	not americas	not americas
California	Mesoamerica	0.2196	americas	americas
S Australia	Interior New Guinea	0.2207	not americas	not americas
E North America	Greater Abyssinia	0.2238	americas	not americas
S Australia	N Coast Asia	0.2283	not americas	not americas
Alaska-Oregon	Greater Abyssinia	0.2289	americas	not americas
S Australia	S New Guinea	0.2292	not americas	not americas
Indic	Mesoamerica	0.2296	not americas	americas
Europe	Greater Abyssinia	0.2296	not americas	not americas
S Australia	Basin and Plains	0.2301	not americas	americas
N Coast New Guinea	Greater Mesopotamia	0.2311	not americas	not americas
S Australia	Greater Abyssinia	0.2311	not americas	not americas
S New Guinea	Southeast Asia	0.2313	not americas	not americas
SE South America	N Australia	0.2313	americas	not americas
Alaska-Oregon	Greater Mesopotamia	0.232	americas	not americas
Interior New Guinea	N Coast Asia	0.2328	not americas	not americas

Basin and Plains	N Coast Asia	0.233	americas	not americas
Basin and Plains	N Coast New Guinea	0.2336	americas	not americas
S Australia	NE South America	0.2346	not americas	americas
Andean	N Australia	0.2357	americas	not americas
E North America	Greater Mesopotamia	0.236	americas	not americas
Oceania	NE South America	0.2361	not americas	americas
N Australia	Mesoamerica	0.238	not americas	americas
N Coast Asia	Greater Mesopotamia	0.2423	not americas	not americas
N Australia	Alaska-Oregon	0.2427	not americas	americas
California	Inner Asia	0.2443	americas	not americas
S Africa	Mesoamerica	0.2446	not americas	americas
Andean	Mesoamerica	0.2461	americas	americas
N Africa	Alaska-Oregon	0.2479	not americas	americas
S Australia	Greater Mesopotamia	0.2517	not americas	not americas
California	N Coast New Guinea	0.2544	americas	not americas
Greater Abyssinia	N Coast New Guinea	0.2546	not americas	not americas
N Coast Asia	S New Guinea	0.255	not americas	not americas

Inner Asia	Mesoamerica	0.2561	not americas	americas
S Africa	Greater Mesopotamia	0.2571	not americas	not americas
S Australia	Alaska-Oregon	0.2587	not americas	americas
Greater Abyssinia	Mesoamerica	0.2589	not americas	americas
Interior New Guinea	Basin and Plains	0.2607	not americas	americas
E North America	N Coast Asia	0.2612	americas	not americas
S New Guinea	S Africa	0.2617	not americas	not americas
SE South America	Mesoamerica	0.2625	americas	americas
S New Guinea	Oceania	0.2627	not americas	not americas
Europe	Inner Asia	0.2628	not americas	not americas
N Africa	Greater Mesopotamia	0.2648	not americas	not americas
Greater Mesopotamia	African Savannah	0.2665	not americas	not americas
Interior New Guinea	Greater Mesopotamia	0.2688	not americas	not americas
SE South America	N Africa	0.271	americas	not americas
Indic	Oceania	0.2726	not americas	not americas
Alaska-Oregon	N Coast Asia	0.2745	americas	not americas
Interior New Guinea	E North America	0.2756	not americas	americas

Inner Asia	N Coast New Guinea	0.2773	not americas	not americas
S Australia	Inner Asia	0.2775	not americas	not americas
S Australia	Indic	0.2797	not americas	not americas
Greater Abyssinia	African Savannah	0.2803	not americas	not americas
Mesoamerica	Southeast Asia	0.2804	americas	not americas
S Australia	E North America	0.2804	not americas	americas
Alaska-Oregon	S New Guinea	0.2843	americas	not americas
SE South America	Andean	0.2847	americas	americas
SE South America	Greater Mesopotamia	0.2882	americas	not americas
Europe	N Australia	0.2944	not americas	not americas
Basin and Plains	Indic	0.2962	americas	not americas
N Australia	N Coast Asia	0.2968	not americas	not americas
Europe	Mesoamerica	0.2974	not americas	americas
SE South America	S New Guinea	0.2987	americas	not americas
Basin and Plains	Inner Asia	0.2988	americas	not americas
E North America	N Coast New Guinea	0.3002	americas	not americas
N Australia	Indic	0.3039	not americas	not americas

Andean	N Coast New Guinea	0.304	americas	not americas
Interior New Guinea	Alaska-Oregon	0.3062	not americas	americas
SE South America	Greater Abyssinia	0.3069	americas	not americas
N Australia	Inner Asia	0.3094	not americas	not americas
Europe	NE South America	0.312	not americas	americas
Oceania	S Africa	0.3155	not americas	not americas
Europe	S New Guinea	0.317	not americas	not americas
Greater Abyssinia	S Africa	0.3204	not americas	not americas
N Australia	S Africa	0.3214	not americas	not americas
N Australia	African Savannah	0.3227	not americas	not americas
Indic	S Africa	0.328	not americas	not americas
S Australia	N Coast New Guinea	0.3323	not americas	not americas
Interior New Guinea	Inner Asia	0.3356	not americas	not americas
Basin and Plains	S Africa	0.3394	americas	not americas
S Africa	Southeast Asia	0.3398	not americas	not americas
SE South America	Interior New Guinea	0.3428	americas	not americas
SE South America	N Coast Asia	0.3439	americas	not americas

Alaska-Oregon	N Coast New Guinea	0.3445	americas	not americas
Basin and Plains	Europe	0.3457	americas	not americas
Inner Asia	African Savannah	0.3464	not americas	not americas
Andean	Europe	0.3507	americas	not americas
S Australia	Mesoamerica	0.3566	not americas	americas
SE South America	S Australia	0.3583	americas	not americas
N Africa	N Australia	0.3605	not americas	not americas
Interior New Guinea	Mesoamerica	0.3616	not americas	americas
N Africa	Greater Abyssinia	0.3633	not americas	not americas
Europe	African Savannah	0.3634	not americas	not americas
Europe	N Coast New Guinea	0.3689	not americas	not americas
N Africa	California	0.3695	not americas	americas
Europe	Indic	0.3739	not americas	not americas
N Coast Asia	Mesoamerica	0.3742	not americas	americas
SE South America	N Coast New Guinea	0.3778	americas	not americas
Europe	Alaska-Oregon	0.3779	not americas	americas
N Africa	Basin and Plains	0.3814	not americas	americas

California	Europe	0.3835	americas	not americas
E North America	Indic	0.3872	americas	not americas
Inner Asia	S Africa	0.3907	not americas	not americas
S Australia	Europe	0.3946	not americas	not americas
N Africa	E North America	0.3956	not americas	americas
Europe	S Africa	0.3965	not americas	not americas
Basin and Plains	African Savannah	0.3972	americas	not americas
Inner Asia	Southeast Asia	0.3975	not americas	not americas
E North America	S Africa	0.3998	americas	not americas
Interior New Guinea	African Savannah	0.4038	not americas	not americas
Interior New Guinea	Europe	0.4124	not americas	not americas
Inner Asia	E North America	0.4128	not americas	americas
N Coast Asia	N Coast New Guinea	0.4161	not americas	not americas
N Australia	Oceania	0.4183	not americas	not americas
Inner Asia	Alaska-Oregon	0.4324	not americas	americas
California	African Savannah	0.4363	americas	not americas
California	Southeast Asia	0.4401	americas	not americas

Europe	N Coast Asia	0.4411	not americas	not americas
California	Oceania	0.4449	americas	not americas
Basin and Plains	Oceania	0.4477	americas	not americas
N Australia	Southeast Asia	0.4478	not americas	not americas
S Australia	N Africa	0.4504	not americas	not americas
Europe	E North America	0.4604	not americas	americas
California	S Africa	0.4635	americas	not americas
Andean	African Savannah	0.4684	americas	not americas
N Africa	Europe	0.4703	not americas	not americas
Interior New Guinea	Southeast Asia	0.4707	not americas	not americas
Alaska-Oregon	Oceania	0.4728	americas	not americas
N Africa	Andean	0.4772	not americas	americas
Oceania	Greater Mesopotamia	0.4873	not americas	not americas
N Africa	Interior New Guinea	0.4901	not americas	not americas
Southeast Asia	Greater Mesopotamia	0.4909	not americas	not americas
E North America	African Savannah	0.4926	americas	not americas
Alaska-Oregon	Indic	0.4938	americas	not americas

Greater Abyssinia	Southeast Asia	0.4941	not americas	not americas
N Africa	Mesoamerica	0.4988	not americas	americas
Inner Asia	Oceania	0.4991	not americas	not americas
N Africa	African Savannah	0.5031	not americas	not americas
Basin and Plains	Southeast Asia	0.5034	americas	not americas
Alaska-Oregon	S Africa	0.5158	americas	not americas
N Africa	NE South America	0.516	not americas	americas
Alaska-Oregon	African Savannah	0.5221	americas	not americas
SE South America	S Africa	0.5229	americas	not americas
N Africa	N Coast Asia	0.5243	not americas	not americas
Andean	S Africa	0.5312	americas	not americas
Andean	Southeast Asia	0.5445	americas	not americas
S Australia	African Savannah	0.5522	not americas	not americas
Greater Abyssinia	Oceania	0.5553	not americas	not americas
S Australia	Southeast Asia	0.5574	not americas	not americas
S Australia	Oceania	0.5617	not americas	not americas
N Africa	N Coast New Guinea	0.5711	not americas	not americas

N Africa	S New Guinea	0.5728	not americas	not americas
SE South America	African Savannah	0.5771	americas	not americas
Interior New Guinea	S Africa	0.5828	not americas	not americas
S Australia	S Africa	0.585	not americas	not americas
SE South America	Europe	0.585	americas	not americas
N Coast Asia	Southeast Asia	0.5952	not americas	not americas
Andean	Oceania	0.6095	americas	not americas
Interior New Guinea	Oceania	0.6145	not americas	not americas
E North America	Oceania	0.6242	americas	not americas
SE South America	Indic	0.6359	americas	not americas
N Africa	S Africa	0.6406	not americas	not americas
N Coast Asia	African Savannah	0.6509	not americas	not americas
E North America	Southeast Asia	0.6699	americas	not americas
N Coast Asia	S Africa	0.7047	not americas	not americas
SE South America	Inner Asia	0.706	americas	not americas
Europe	Oceania	0.7061	not americas	not americas
SE South America	Oceania	0.7213	americas	not americas

Alaska-Oregon	Southeast Asia	0.7451	americas	not americas
Europe	Southeast Asia	0.747	not americas	not americas
N Coast Asia	Oceania	0.7992	not americas	not americas
N Africa	Inner Asia	0.9289	not americas	not americas
SE South America	Southeast Asia	0.9627	americas	not americas
N Africa	Indic	1.0179	not americas	not americas
N Africa	Oceania	1.0647	not americas	not americas
N Africa	Southeast Asia	1.5549	not americas	not americas

Table S8. Coefficients and associated error estimates for the spatiophylogenetic Bayesian regression model predicting Unusualness scores.

Coefficient	Estimate	Estimated error
Intercept	4.73	0.21
SD	0.24	0.01
SD (phylogeny)	0.08	0.01
SD (spatial)	0.15	0.02

Table S9. Example of theoretical metric calculation.

Feature	word-	poko1263	hind1269	khak1248

	order-point	Language-value	word-order-value	Language-value	word-order-value	Language-value	word-order-value
**GB022 Are there prenominal articles**	**0**	0	1	0	1	1	0
**GB133 Is a pragmatically unmarked constituent order verb-final for transitive clauses?**	**0**	0	1	1	0	1	0
**GB023Are there postnominal articles**	**1**	1	1	0	0	0	0
**GB262 Is there a clause-initial polar interrogative particle?**	**1**	1	1	1	1	0	0
**mean word order score**			**1**		**0.5**		**0**

Table S10. Language pairs with a Manhattan distance of 0.

Glottocodes	Names	Family name
pahn1237-biao1256	Pa-Hng-Biao Mon	Hmong-Mien
xish1235-cosa1234	Xishanba Lalo-Cosao	Sino-Tibetan
kusa1251-hoav1238	Kusaghe-Njela-Hoava	Austronesian
kare1335-ingr1248	Karelian-Ingrian	Uralic
sout2959-nort2942	South Slavey-North Slavey	Athabaskan-Eyak-Tlingit
puni1241-phoe1239	Punic-Phoenician	Afro-Asiatic

REFERENCES AND NOTES

1. H. Hammarström, R. Forkel, M. Haspelmath, S. Bank, glottolog/glottolog: Glottolog Database 4.5 as CLDF (v4.5) [Data set] (Zenodo, 2021); https://doi.org/10.5281/zenodo.5772649.

2. M. H. Christiansen, N. Chater, Language as shaped by the brain. *Behav. Brain Sci.* **31**, 489–509 (2008).

3. B. Bickel, A. Witzlack-Makarevich, K. K. Choudhary, M. Schlesewsky, I. Bornkessel-Schlesewsky, The neurophysiology of language processing shapes the evolution of grammar: Evidence from case marking. *PLOS ONE* **10**, e0132819 (2015).

4. J. Nichols, *Linguistic Diversity in Space and Time* (University of Chicago Press, 1992).

5. P. Muysken, *From Linguistic Areas to Areal Linguistics* (John Benjamins, 2008).

6. D. Dediu, S. C. Levinson, Abstract profiles of structural stability point to universal tendencies, family-specific factors, and ancient connections between languages. *PLOS ONE* **7**, e45198 (2012).

7. D. Dediu, M. Cysouw, Some structural aspects of language are more stable than others: A comparison of seven methods. *PLOS ONE* **8**, e55009 (2013).

8. M. S. Dryer, M. Haspelmath, Eds., The World Atlas of Language Structures Online (v2020.1) [Data set] (Zenodo, 2021); https://doi.org/10.5281/zenodo.4683137.

9. H. Hammarström, Sampling and genealogical coverage in *WALS*. *Linguist. Typology* **13**, 105–119 (2009).

10. M. D. Auger, Cultural continuity as a determinant of Indigenous Peoples' health: A metasynthesis of qualitative research in Canada and the United States. *Int. Indig. Policy J.* **7**, 3 (2016).

11. M. Durie, H. Milroy, E. Hunter, Mental health and the indigenous peoples of Australia and New Zealand, in *Healing Traditions: The Mental Health of Aboriginal Peoples in Canada*, L. J. Kirmayer, G. G. Valaskakis, Eds. (UBC Press, 2009), pp. 36–55.

12. N. Evans, *Words of Wonder: Endangered Languages and What They Tell Us* (Wiley-Blackwell, ed. 2, 2022).

13. W. J. Sutherland, Parallel extinction risk and global distribution of languages and species. *Nature* **423**, 276–279 (2003).

14. L. Campbell, A. Belew, Eds., *Cataloging the World's Endangered Languages* (Routledge, 2018).

15. L. Bromham, R. Dinnage, H. Skirgård, A. Ritchie, M. Cardillo, F. Meakins, S. Greenhill, X. Hua, Global predictors of language endangerment and the future of linguistic diversity. *Nat. Ecol. Evol.* **6**, 163–173 (2022).

16. UNESCO, Global action plan of the International Decade of Indigenous Languages (IDIL 2022-2032) (2021); https://unesdoc.unesco.org/ark:/48223/pf0000379851.

17. Q. D. Atkinson, R. D. Gray, Curious parallels and curious connections—Phylogenetic thinking in biology and historical linguistics. *Syst. Biol.* **54**, 513–526 (2005).

18. J. Schmidt, *Die Verwandtschaftsverhältnisse der indogermanischen Sprachen* (H. Böhlau, 1872).

19. A. François, Trees, waves and linkages: Models of language diversification, in *The Routledge Handbook of Historical Linguistics*, C. Bowern, B. Evans, Eds. (Routledge, 2015), pp. 161–189.

20. J. Nichols, Diachronically stable structural features, in *Historical Linguistics, 1993: Selected Papers from the 11th International Conference on Historical Linguistics*, H. Andersen, Ed. (John Benjamins, 1995), pp. 337–356.

21. L. Campbell, *Historical Linguistics: An Introduction* (MIT Press, ed. 3, 2013).

22. M. Dunn, A. Terrill, G. Reesink, R. A. Foley, S. C. Levinson, Structural phylogenetics and the reconstruction of ancient language history. *Science* **309**, 2072–2075 (2005).

23. H. Matsumae, P. Ranacher, P. E. Savage, D. E. Blasi, T. E. Currie, K. Koganebuchi, N. Nishida, T. Sato, H. Tanabe, A. Tajima, S. Brown, M. Stoneking, K. K. Shimizu, H. Oota, B. Bickel, Exploring

correlations in genetic and cultural variation across language families in northeast Asia. *Sci. Adv.* **7**, eabd9223 (2021).

24. S. J. Greenhill, C.-H. Wu, X. Hua, M. Dunn, S. C. Levinson, R. D. Gray, Evolutionary dynamics of language systems. *Proc. Natl. Acad. Sci. U.S.A.* **114**, E8822–E8829 (2017).

25. R. Dinnage, A. Skeels, M. Cardillo, Spatiophylogenetic modelling of extinction risk reveals evolutionary distinctiveness and brief flowering period as threats in a hotspot plant genus. *Proc. R. Soc. B* **287**, 20192817 (2020).

26. R. Bouckaert, D. Redding, O. Sheehan, T. Kyritsis, R. Gray, K. E. Jones, Q. Atkinson, Global language diversification is linked to socio-ecology and threat status (2022); doi:10.31235/osf.io/f8tr6.

27. P. Muysken, Three processes of borrowing: Borrowability revisited, in *Bilingualism and Migration*, G. Extra, L. Verhoeven, Eds. (De Gruyter Inc., 1999), pp. 229–246.

28. F. Meakins, J. Stewart, Mixed languages, in *The Cambridge Handbook of Language Contact: Volume 2: Multilingualism in Population Structure*, A. M. Escobar, S. Mufwene, Eds. (Cambridge Univ. Press, 2022), pp. 310–343.

29. D. C. Dennett, *Darwin's Dangerous Idea: Evolution and the Meanings of Life* (Simon & Schuster, 1995).

30. A. Meillet, *Introduction à L'étude Comparative des Langues Indo-européennes* (Hachette, 1903).

31. C.-T. J. Huang, I. Roberts, Principles and parameters of universal grammar, in *Oxford Handbook of Universal Grammar*, I. Roberts, Ed. (Oxford Univ. Press, 2016), pp. 306–354.

32. M. C. Baker, *The Atoms of Language: The Mind's Hidden Rules of Grammar* (Oxford Univ. Press, 2001).

33. J.-L. Mendívil-Giró, Why don't languages adapt to their environment? *Front. Commun.* **3**, 24 (2018).

34. N. J. Enfield, *Natural Causes of Language* (Language Science Press, 2014).

35. G. Raîche, T. A. Walls, D. Magis, M. Riopel, J.-G. Blais, Non-graphical solutions for Cattell's scree test. *Methodology* **9**, 23–29 (2013).

36. S. A. Mehr, M. Singh, D. Knox, D. M. Ketter, D. Pickens-Jones, S. Atwood, C. Lucas, N. Jacoby, A. A. Egner, E. J. Hopkins, R. M. Howard, J. K. Hartshorne, M. V. Jennings, J. Simson, C. M. Bainbridge, S. Pinker, T. J. O'Donnell, M. M. Krasnow, L. Glowacki, Universality and diversity in human song. *Science* **366**, eaax0868 (2019).

37. J. Novembre, T. Johnson, K. Bryc, Z. Kutalik, A. R. Boyko, A. Auton, A. Indap, K. S. King, S. Bergmann, M. R. Nelson, M. Stephens, C. D. Bustamante, Genes mirror geography within Europe. *Nature* **456**, 98–101 (2008).

38. J. H. Greenberg, Some universals of grammar with particular reference to the order of meaningful elements, in *Universals of Language*, J. H. Greenberg, Ed. (MIT Press, 1963), pp. 73–113.

39. M. S. Dryer, The Greenbergian word order correlations. *Language* **68**, 81–138 (1992).

40. J. Nichols, Head-marking and dependent-marking grammar. *Language* **62**, 56–119 (1986).

41. B. Bickel, J. Nichols, Inflectional morphology, in *Language Typology and Syntactic Description: Volume 3: Grammatical Categories and the Lexicon*, T. Shopen, Ed. (Cambridge Univ. Press, 2007), pp. 169–240.

42. E. Sapir, *Language: An Introduction to the Study of Speech* (Harcourt, Brace and Co., 1921).

43. S. J. Gould, *Wonderful Life: The Burgess Shale and the Nature of History* (W.W. Norton & Co., 1990).

44. M. Muthukrishna, A. V. Bell, J. Henrich, C. M. Curtin, A. Gedranovich, J. McInerney, B. Thue, Beyond western, educated, industrial, rich, and democratic (WEIRD) psychology: Measuring and mapping scales of cultural and psychological distance. *Psychol. Sci.* **31**, 678–701 (2020).

45. B. Bickel, J. Nichols, T. Zakharko, A. Witzlack-Makarevich, K. Hildebrandt, M. Reißler, L. Bierkandt, F. Zúñiga, J. B. Lowe, The AUTOTYP Typological Database (v1.0.1) [Data set] (Zenodo, 2022); http://doi.org/10.5281/zenodo.6255206.

46. N. W. H. Mason, D. Mouillot, W. G. Lee, J. B. Wilson, Functional richness, functional evenness and functional divergence: The primary components of functional diversity. *Oikos* **111**, 112–118 (2005).

47. S. Villéger, N. W. H. Mason, D. Mouillot, New multidimensional functional diversity indices for a multifaceted framework in functional ecology. *Ecology* **89**, 2290–2301 (2008).

48. C. Pimiento, F. Leprieur, D. Silvestro, J. S. Lefcheck, C. Albouy, D. B. Rasher, M. Davis, J.-C. Svenning, J. N. Griffin, Functional diversity of marine megafauna in the Anthropocene. *Sci. Adv.* **6**, eaay7650 (2020).

49. D. J. Stekhoven, missForest: Nonparametric missing value imputation using random forest (2013); https://cran.r-project.org/web/packages/missForest/index.html.

50. D. J. Stekhoven, P. Bühlmann, MissForest—Non-parametric missing value imputation for mixed-type data. *Bioinformatics* **28**, 112–118 (2012).

51. T. G. Martins, D. Simpson, F. Lindgren, H. Rue, Bayesian computing with INLA: New features. *Comput. Stat. Data Anal.* **67**, 68–83 (2013).

52. E. Paradis, K. Schliep, ape 5.0: An environment for modern phylogenetics and evolutionary analyses in R. *Bioinformatics* **35**, 526–528 (2019).

53. P. J. Ribeiro, P. J. Diggle, O. Christensen, M. Schlather, R. Bivand, B. Ripley, geoR: Analysis of geostatistical data (2020); https://cran.r-project.org/web/packages/geoR/index.html.

54. M. W. Pennell, J. M. Eastman, G. J. Slater, J. W. Brown, J. C. Uyeda, R. G. FitzJohn, M. E. Alfaro, L. J. Harmon, geiger v2.0: An expanded suite of methods for fitting macroevolutionary models to phylogenetic trees. *Bioinformatics* **30**, 2216–2218 (2014).

55. S. A. Fritz, A. Purvis, Selectivity in mammalian extinction risk and threat types: A new measure of phylogenetic signal strength in binary traits. *Conserv. Biol.* **24**, 1042–1051 (2010).

56. R Core Team, R: A language and environment for statistical computing (2021); www.R-project.org/.

57. R. P. Freckleton, P. H. Harvey, M. Pagel, Phylogenetic analysis and comparative data: A test and review of evidence. *Am. Nat.* **160**, 712–726 (2002).

58. A. V. Bell, P. J. Richerson, R. McElreath, Culture rather than genes provides greater scope for the evolution of large-scale human prosociality. *Proc. Natl. Acad. Sci. U.S.A.* **106**, 17671–17674 (2009).

59. G. Csardi, T. Nepusz, The igraph software package for complex network research. *InterJ. Complex Syst.* **1695**, 1–9 (2006).

60. P.-C. Bürkner, brms: An R package for Bayesian multilevel models using Stan. *J. Stat. Softw.* **80**, 1–28 (2017).

61. A. Gelman, B. Goodrich, J. Gabry, A. Vehtari, R-squared for Bayesian regression models. *Am. Stat.* **73**, 307–309 (2019).

62. M. Grenié, H. Gruson, fundiversity: A modular R package to compute functional diversity indices (2022); doi:10.32942/osf.io/dg7hw.

63. H. Hammarström, T. Castermans, R. Forkel, K. Verbeek, B. Speckmann, Simultaneous visualization of language endangerment and language description. *Lang. Doc. Conserv.* **12**, 359–392 (2018).

64. R. Forkel, J.-M. List, S. J. Greenhill, C. Rzymski, S. Bank, M. Cysouw, H. Hammarström, M. Haspelmath, G. A. Kaiping, R. D. Gray, Cross-Linguistic Data Formats, advancing data sharing and re-use in comparative linguistics. *Sci. Data* **5**, 180205 (2018).

65. K. R. Kirby, R. D. Gray, S. J. Greenhill, F. M. Jordan, S. Gomes-Ng, H.-J. Bibiko, D. E. Blasi, C. A. Botero, C. Bowern, C. R. Ember, D. Leehr, B. S. Low, J. McCarter, W. Divale, M. C. Gavin, D-PLACE: A global database of cultural, linguistic and environmental diversity. *PLOS ONE* **11**, e0158391 (2016).

66. S. Danielsen, M. Dunn, P. Muysken, The spread of the Arawakan languages: A view from structural phylogenetics, in *Ethnicity in Ancient Amazonia: Reconstructing Past Identities from Archaeology, Linguistics, and Ethnohistory*, A. Hornborg, J. D. Hill, Eds. (University Press of Colorado, 2011), pp. 173–196.

67. H. Hammarström, G. Reesink, M. Dunn, H. Skirgård, S. van der Meer, J. Lesage, J. Peacock, R. Singer, H. de Vos, *Nijmegen Typological Survey* (Max Planck Institute for Psycholinguistics, 2017); https://hdl.handle.net/1839/935A5B75-9624-4C5E-AEB7-AB28C2D8C209.

68. J. L. Fleiss, Measuring nominal scale agreement among many raters. *Psychol. Bull.* **76**, 378–382 (1971).

69. J. R. Landis, G. G. Koch, The measurement of observer agreement for categorical data. *Biometrics* **33**, 159–174 (1977).

70. F. Plank, WALS values evaluated. *Linguist. Typology* **13**, 41–75 (2009).

71. V. N. Polyakov, V. D. Solovyev, S. Wichmann, O. Belyaev, Using WALS and Jazyki Mira. *Linguist. Typology* **13**, 137–167 (2009).

72. R. Forkel, S. Bank, C. Rzymski, H.-J. Bibiko, clld/clld: clld—A toolkit for cross-linguistic databases (2020); doi:10.5281/zenodo.3968247.
